# Supplementary material for: Physiological regulation of neuronal Wnt activity is essential for TDP-43 localization and function
Source: EMBO J. 2024 Jun 25;43(16):6. doi: 10.1038/s44318-024-00156-8 (PMC11329687; doi:10.1038/s44318-024-00156-8)
Supplement: Supplementary file 1 — Appendix [file 44318_2024_156_MOESM1_ESM.pdf]

Physiological regulation of neuronal Wnt activity is essential for TDP-43 localization and function

Manuscript: EMBOJ-2024-116687

Appendix

Table of Contents

Appendix Figure S1 2

Appendix Figure S2 3

Appendix Figure S3 5

Appendix Figure S4 7

Appendix Table S1.. 8

Appendix Table S2.. 9

Appendix Table S3.. 11

Appendix Table S4.. 12

Appendix References..14

Appendix Figures

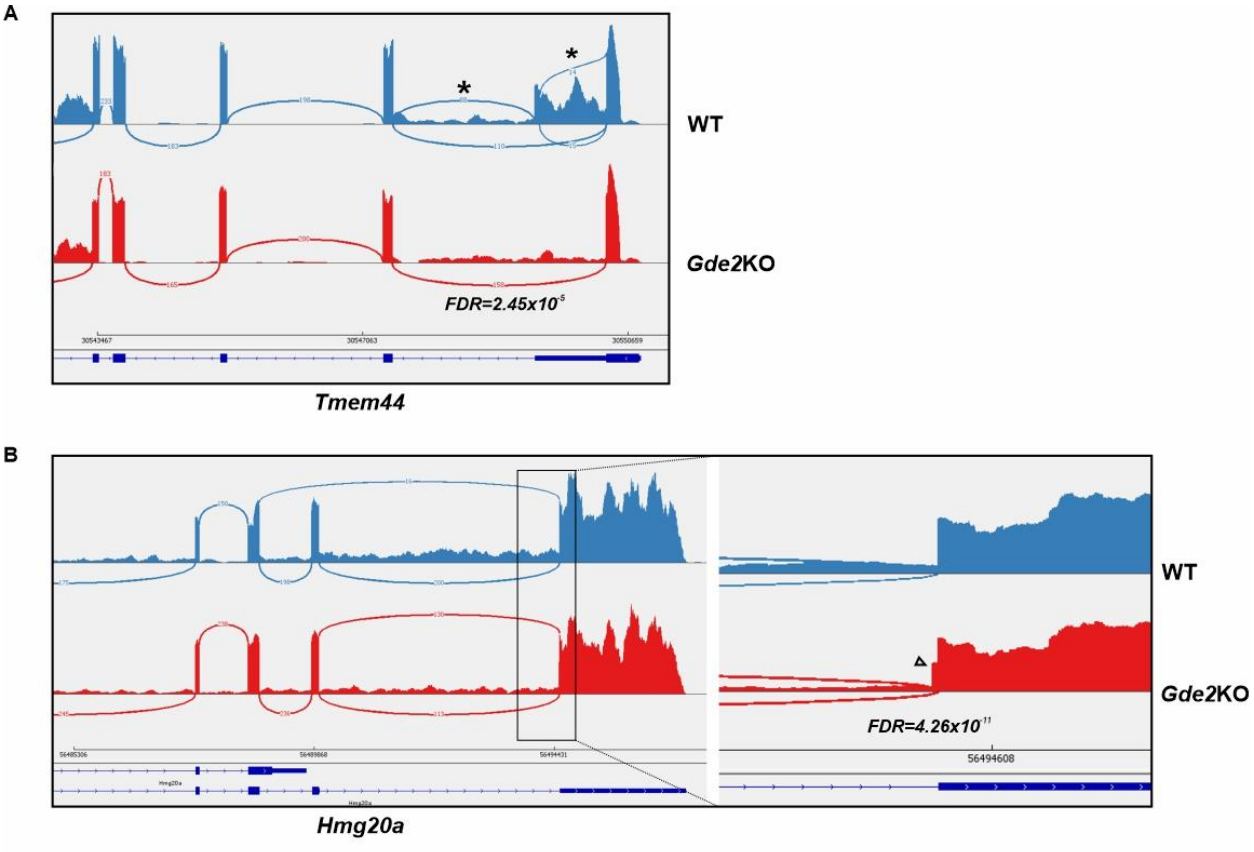

Appendix Fig. S1. Splicing diagrams of exemplar mis-spliced transcripts in *Gde2*KO animals.

Examples of two genes with alternatively spliced introns that contain the TG-repeat motif from RNAseq data derived from 19-month WT and *Gde2*KO cortices. Sashimi plots created from IGV showing the genome locus, isoforms, and exons of listed genes, RNAseq read densities along the exons and splice junctions between the exons. Numbers on the arcs represent the junction read counts. **A-B**. panels showing the splicing diagrams of *Tmem44* with alternatively spliced 5' UTR (**A**), and *Hmg20a* with mis-splicing at its 3' UTR (**B**). Asterisks: splice junctions present in the WTs but not in *Gde2*KOs; arrowhead: mis-splicing in *Gde2*KOs.

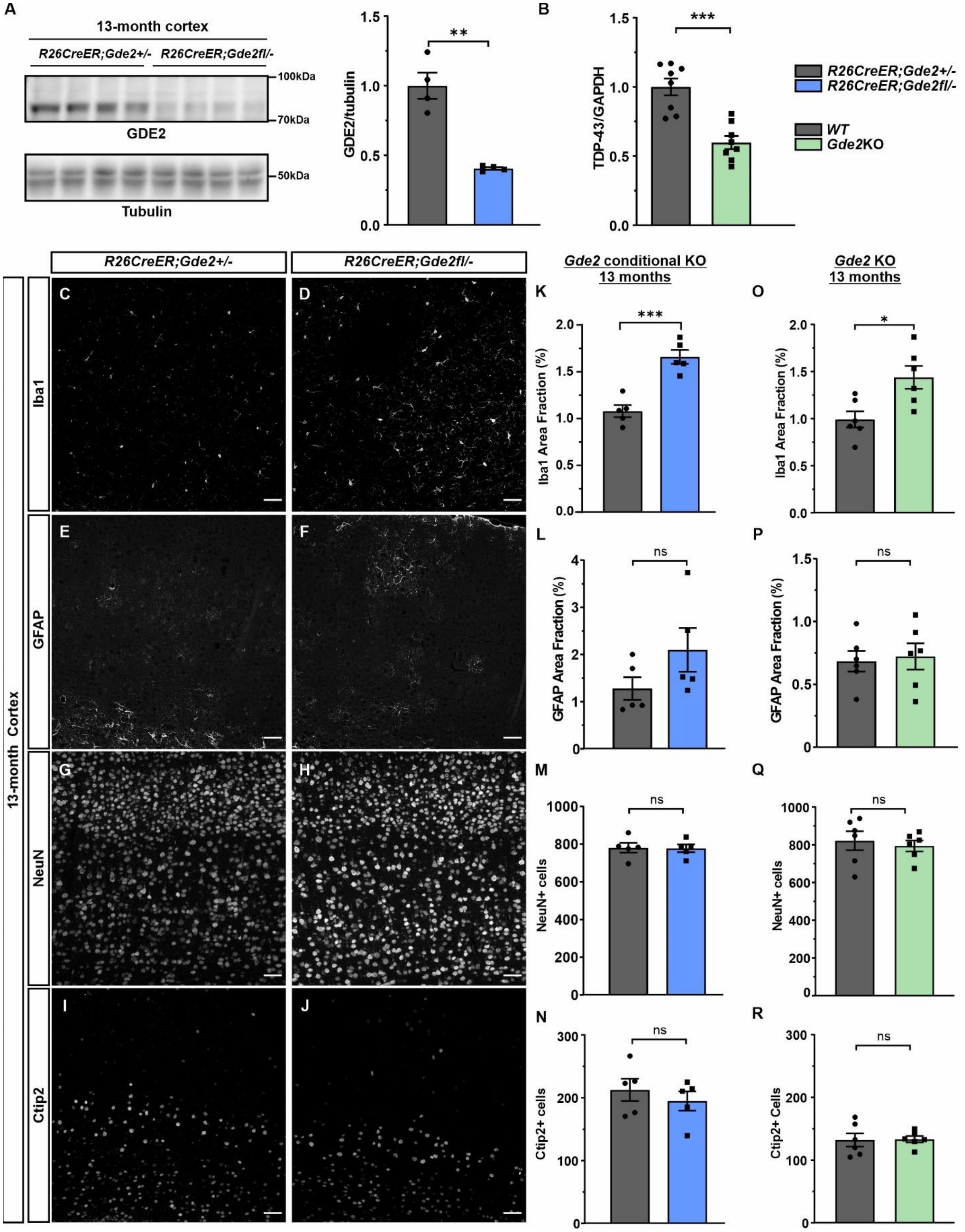

Appendix Fig. S2. GDE2 expression in the adult is required to prevent neurodegenerative changes.

A. Western blot of cortical extracts from 13-month conditional *Gde2*KO animals (*R26CreER;Gde2fl/-*) where GDE2 function is genetically ablated at 2 months by tamoxifen delivery. *R26CreER;Gde2+/-* animals injected with tamoxifen at 2 months are controls. Graph quantifying GDE2 amounts normalized to Tubulin. **p = 0.0077 n = 4/genotype. **B.** Graph quantifying TDP-43 protein normalized to GAPDH from western blot from global 13-month-old *Gde2*KO animals, ***p = 0.0001, n = 8. **C-J**. Representative images of immunohistochemical staining of cortical sections of 13-month conditional *Gde2*KO animals and controls. **K-N.** Graphs quantifying microglial activation (**K**, Iba1, ***p = 0.0004), astrogliosis (**L**, GFAP ns p = 0.1542), neuronal numbers (**M**, NeuN, ns p = 0.9259) and deep layer neurons (**N**, Ctip2, ns p = 0.4720) n = 5/genotype. **O-R.** Graphs of 13-month WT and global *Gde2*KOs, quantifying microglial activation (**O**, Iba1, *p = 0.0131), astrogliosis (**P**, GFAP ns p = 0.7778), neuronal numbers (**Q**, NeuN, ns p = 0.6482) and deep layer neurons (**R**, Ctip2, ns p = 0.9102) n = 5-6/genotype. All graphs: mean $\pm$ sem, **B, K-N**, **O-R**: Unpaired t-test; **A**: Welch's t-test. Scale bar: **C-J** = 50μm**.**

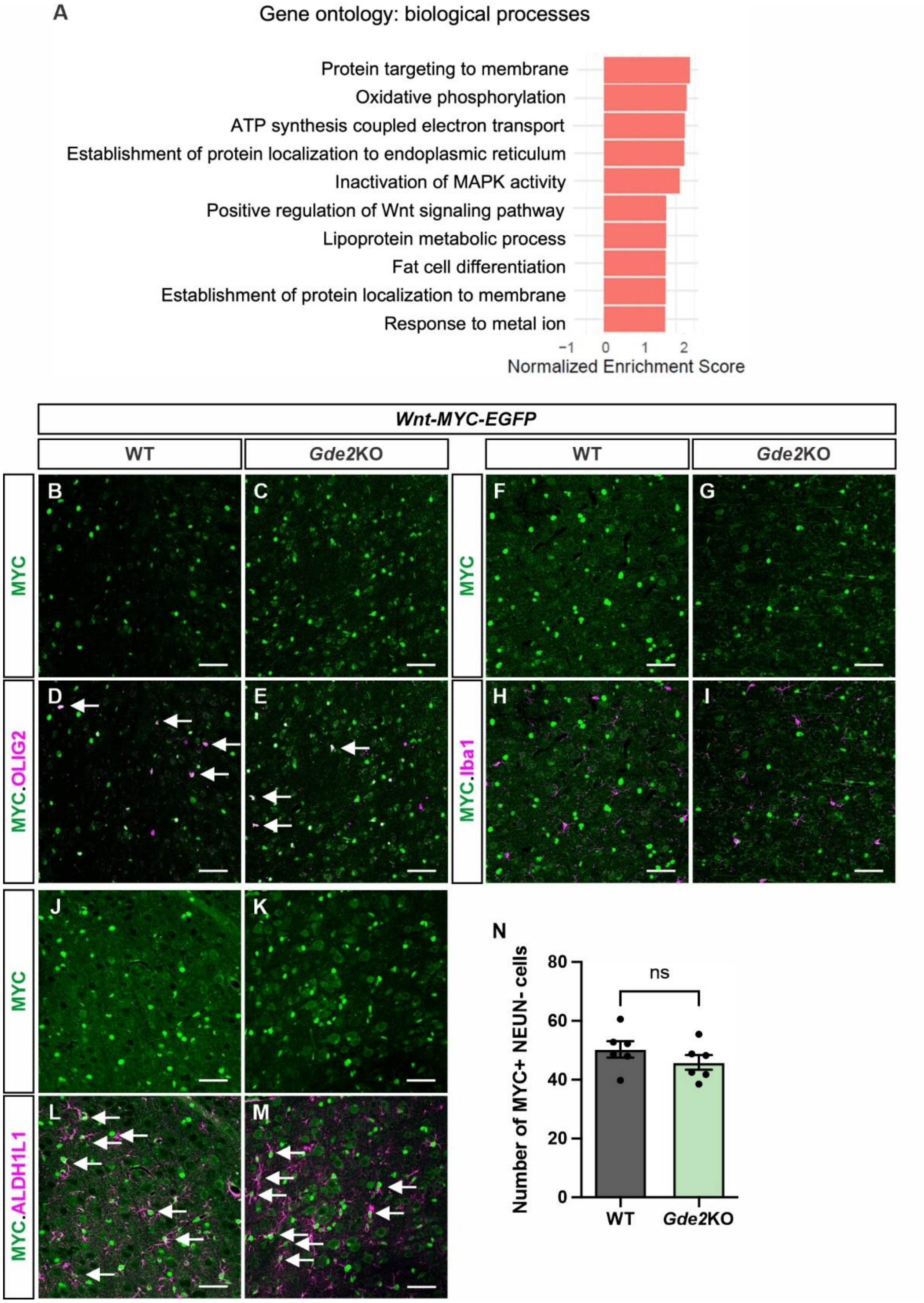

Appendix Fig. S3. GDE2 loss leads to aberrant activation of canonical Wnt signaling in neurons, with no apparent changes in non-neuronal cells.

A. Gene set enrichment analysis (GSEA) of RNAseq data from 4-month-old WT and *Gde2*KO cortex. n = 3. **B-M**. Representative images of immunohistochemical staining of cortical sections

of 4-month WT and *Gde2*KO animals harboring the Wnt reporter *Wnt-MYC-EGFP*. Arrows highlight Olig2+ oligodendroglia (**D, E**) and ALDH1L1 astrocytes (**L, M**) with reporter gene expression (MYC). No Iba1+ microglia show reporter gene expression (**H, I**). Scale bar: **B-M** = 50μm: **N.** Graph quantifying the number of non-neuronal cells with reporter gene expression. ns p = 0.2609. mean ± sem, Unpaired t-test. n = 6 per genotype.

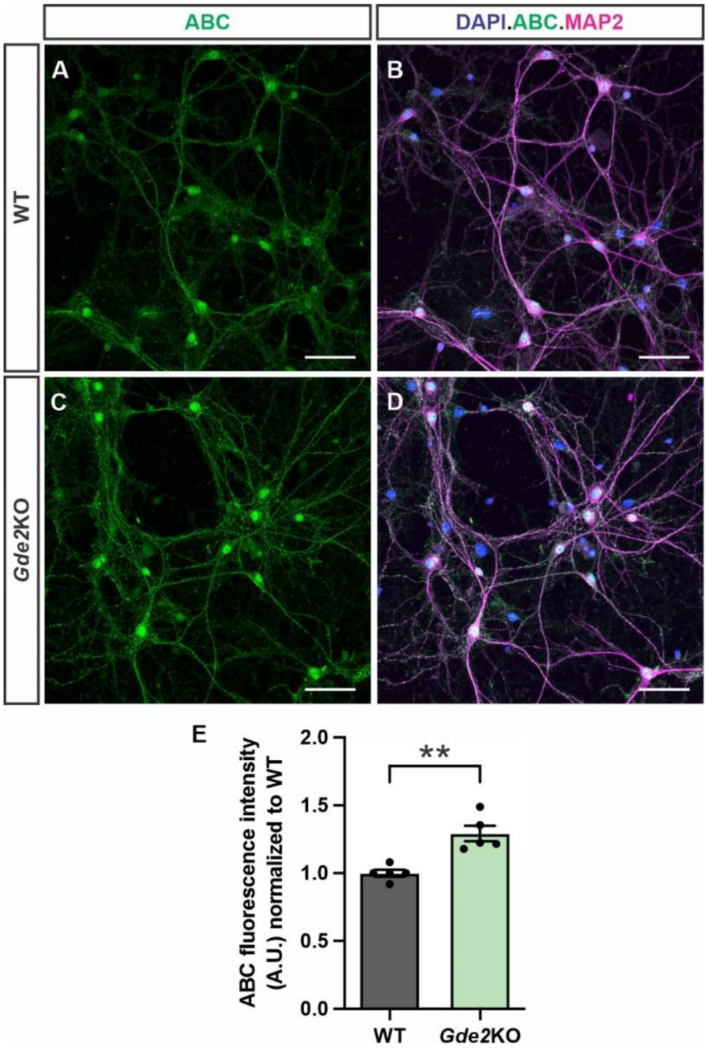

Appendix Fig. S4. Cultured *Gde2*KO cortical neurons show increased expression of activated β-catenin.

A-D. Representative images of immunocytochemical staining of primary cultures of WT and *Gde2*KO cortical neurons at 21 days in vitro (DIV). ABC: Activated β-catenin; Scale bar = 50μm**.**

E. Graph quantifying the nuclear fluorescence intensity of activated β-catenin (ABC). A.U. = arbitrary units **p = 0.0043. mean ± sem, Welch's t-test. n = 5 independent cultures with ≥50 cells per genotype per culture.

Appendix Tables

Appendix Table S1: Patient demographic information

Subject ID	Sample type	Group	Gender	Race	Age
CTL1	Paraffin section	control	Male	White	77
CTL2	Paraffin section	control	Female	White	76
CTL3	Paraffin section	control	Female	White	80
CTL4	Paraffin section	control	Female	White	59
CTL5	Paraffin section	control	Male	White	74
ALS1	Paraffin section	ALS c9	Female	White	68
ALS2	Paraffin section	sALS	Female	White	70
ALS3	Paraffin section	ALS c9	Male	White	59
ALS4	Paraffin section	ALS c9	Female	White	61
ALS5	Paraffin section	sALS	Female	Black/African American	72
ALS6	Paraffin section	sALS	Female	White	68

Appendix Table S2. Antibody Information

Primary Antibodies			
**Antibody**	**Source**	**Catalog number**	**Application and concentration**
TDP-43	Proteintech	10782-2-AP	Immunoblotting: 1:5000<br>IHC: 1:500
Phospho-TDP-43 (Ser409/410)	BioLegend	829901	IHC: 1:200
TDP-43 (C-terminal)	Proteintech	12892-1-AP	IHC: 1:500
Nup98	Abcam	ab50610	Immunoblotting: 1:2500<br>IHC: 1:500
Total β-catenin	Cell Signaling Technologies	8480	Immunoblotting: 1:5000
non-phospho-β-catenin	Cell Signaling Technologies	8814	Immunoblotting: 1:1000
Active-β-Catenin	Millipore Sigma	05-665	ICC: 1:200
LRP6	Cell Signaling Technologies	2560	Immunoblotting: 1:5000
phospho-LRP6	Cell Signaling Technologies	2568	Immunoblotting: 1:1000
ISLET1	R&D Systems	AF1837	Immunoblotting: 1:2500<br>ICC: 1:250
NKX6.1	DSHB	F55A10	Immunoblotting: 1:5000<br>ICC: 1:1000
Mouse GDE2	Covance		Immunoblotting: 1:1000
GAPDH	Cell Signaling Technologies	8884	Immunoblotting: 1:5000
α-Tubulin	Millipore Sigma	T9026	Immunoblotting: 1:5000
hFAB Rhodamine anti-Actin	BioRad	12004163	Immunoblotting: 1:2500
hFAB Rhodamine anti-Tubulin	BioRad	12004166	Immunoblotting: 1:5000
Human GDE2	Covance	cSS1	IHC: 1:300
GFAP	BD Pharmigen	556328	IHC: 1:500
Iba1	Wako	019-19741	IHC: 1:1000
NeuN	Synaptic Systems	266 004	IHC: 1:1000
Ctip2	abcam	ab18465	IHC: 1:1000
Ran	BD Transduction	610341	IHC: 1:500
DsRed	Clontech	632496	IHC: 1:1000
MYC	DSHB	9E10	IHC: 1:1000
GFP	Invitrogen	A11120 or A11122	IHC: 1:500
MAP2	Synaptic Systems	188 004	ICC: 1:5000
Neurofilament H (previously SMI-32)	Biolegend	801701	ICC: 1:1000
βIII-Tubulin	Millipore Sigma	AB9354	ICC: 1:1000
P62	MBL Life Science	PM045	ICC: 1:500
G3BP	BD Biosciences	611126	ICC: 1:500

eIF3η	Santa Cruz Biotechnology	sc-137214	ICC: 1:200

Appendix Table S3: iPSC line information

	iPSC line name	Clinical Diagnosis	Age at sampling	Gender	Source
Ctrl-1	CS8PAAiCTR	non-neurologic control	58	female	Cedars-Sinai iPSC Core
Ctrl-2	CS2AE8iCTR	non-neurologic control	50	female	Cedars-Sinai iPSC Core
Ctrl-3	CS1ATZiCTR	non-neurologic control	60	male	Cedars-Sinai iPSC Core
Ctrl-4	CS9XH7iCTR	non-neurologic control	53	male	Cedars-Sinai iPSC Core
C9-1	CS6CLWiALS	C9orf72 repeat expansion	52	male	Cedars-Sinai iPSC Core
C9-2	CS0NKCiALS	C9orf72 repeat expansion	52	female	Cedars-Sinai iPSC Core
C9-3	CS2YNLiALS	C9orf72 repeat expansion	60	male	Cedars-Sinai iPSC Core
C9-4	CS8KT3iALS	C9orf72 repeat expansion	60	male	Cedars-Sinai iPSC Core

Appendix Table S4: qRT-PCR and RT-PCR primer sequences

	F/R	Sequence (5'->3')	Citations
GAPDH	F	GAAGGTGAAGGTCGGAGTC	
	R	GAAGATGGTGATGGGATTTC	
TUBB3	F	GGCCAAGGGTCACTACACG	Yang et al., 2019 (https://stemcellres.biomedcentral.com/articles/10.1186/s13287-019-1294-x) (Yang *et al*, 2019)
	R	GCAGTCGCAGTTTTCACACTC	
ISLET1	F	CAGGTTGTACGGGATCAAATGC	Patani et al., 2011 (https://www.nature.com/articles/ncomms1216#MOESM151) (Patani *et al*, 2011)
	R	CACACAGCGGAAACACTCGAT	
STMN2	F	AGCTGTCCATGCTGTCACTG	Melamed et al., 2019 (https://www.nature.com/articles/s41593-018-0293-z)
	R	GGTGGCTTCAAGATCAGCTC	
ELAVL3	F	GCATTGGCGACATCGAGTCC	
	R	GGCTTTGTCTGCATCATTGGG	
UNC13A	F	GGACGTGTGGTACAACCTGG	
	R	GTGTACTGGACATGGTACGGG	
SELPLG	F	CCCACGAGGCAGCTGTCC	
	R	CCCAGTAGGATCAGCAACAGGAG	
RCAN1	F	GGCTCCAGCTGCATAAGACTGA	
	R	GGAGATCAGAAACTGCTTGTCTGGA	
PFKP	F	GCGGGGATGCTCAAGGT	
	R	CGTCCACCATGCCCTGGTAG	
SDHA	F	GAGATGTGGTGTCTCGGTCCAT	
	R	GCTGTCTCTGAAATGCCAGGCA	
TUBB2A	F	CTGGCACCATGGACTCTG	
	R	TCGGCTCCCTCTGTGTAG	
RPLP0	F	TCTACAACCCTGAAGTGCTTGAT	Prudencio et al., 2020 (https://www.jci.org/articles/view/139741)
	R	CAATCTGCAGACAGACACTGG	
Camk1g	F	CTGGCCAAGATCACAGACTGG	Jeong et al., 2017 (https://molecularneurodegeneration.biomedcentral.com/articles/10.1186/s13024-016-0144-x)
	R	CTGTGTAGACACCACGCTCT	

Tecpr1	F	AGTCAGACTGGTACGTGGATGAG	Sinha et al., 2024 (https://www.biorxiv.org/content/10.1101/2024.03.27.587011v1)
	R	GTGGCTGACATCCTCTCGG	
Synj2bp CE	F	CTCCAACGACAGTGGCATCT	Sinha et al., 2024 (https://www.biorxiv.org/content/10.1101/2024.03.27.587011v1)
	R	TCTTCCTGAGGACCTCCGTT	

Appendix References

Patani R, Hollins AJ, Wishart TM, Puddifoot CA, Álvarez S, de Lera AR, Wyllie DJA, Compston DAS, Pedersen RA, Gillingwater TH *et al* (2011) Retinoid-independent motor neurogenesis from human embryonic stem cells reveals a medial columnar ground state. *Nature Communications* 2: 214

Yang H, Hao D, Liu C, Huang D, Chen B, Fan H, Liu C, Zhang L, Zhang Q, An J *et al* (2019) Generation of functional dopaminergic neurons from human spermatogonial stem cells to rescue parkinsonian phenotypes. *Stem Cell Research & Therapy* 10: 195
