# Supplementary figures and images for: Physiological regulation of neuronal Wnt activity is essential for TDP-43 localization and function (part 1 of 2)
Source: EMBO J. 2024 Jun 25;43(16):6. doi: 10.1038/s44318-024-00156-8 (PMC11329687; doi:10.1038/s44318-024-00156-8)

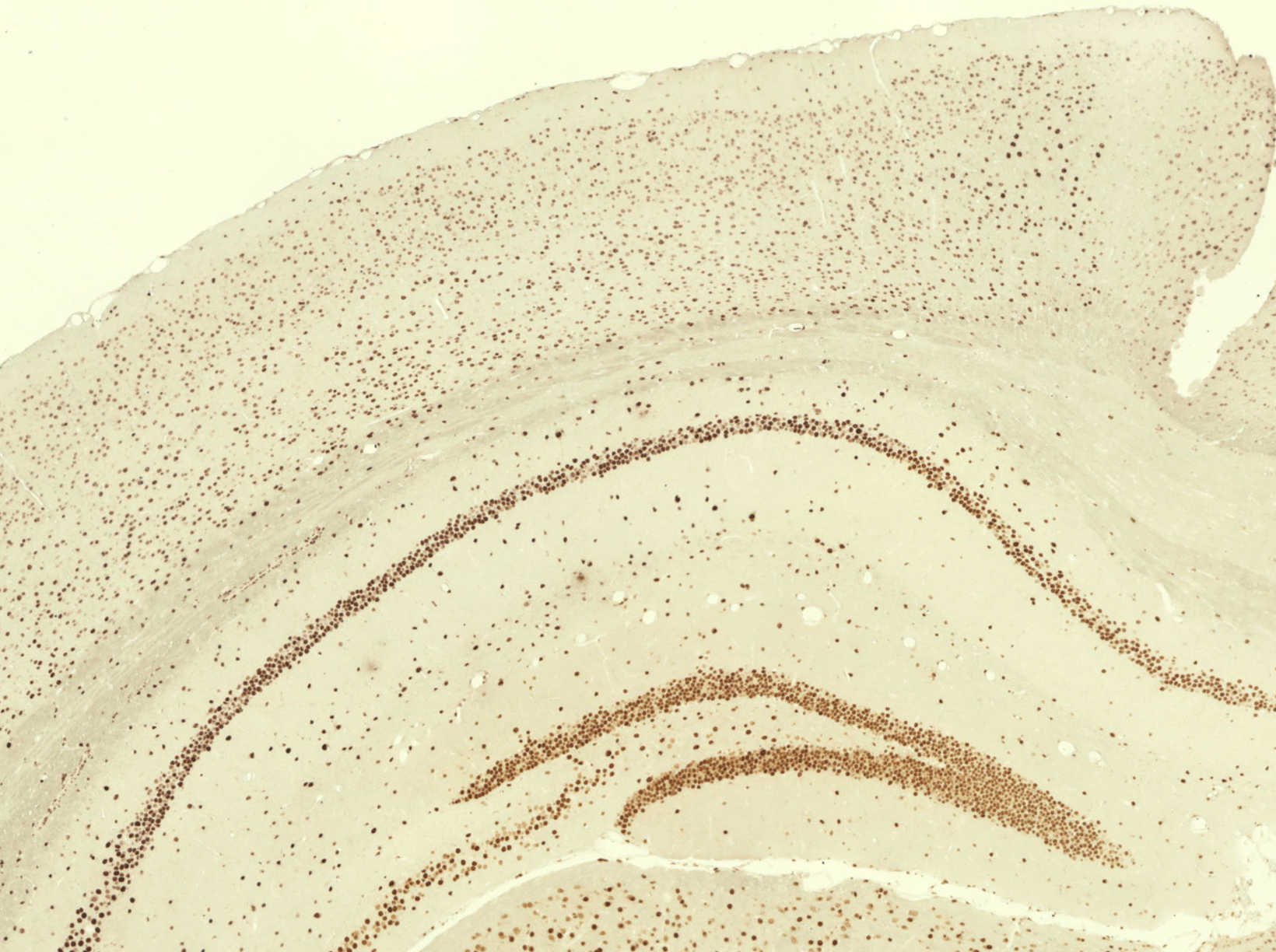

Supplement: Supplementary file 5 — Source data Fig. 1 [file 44318_2024_156_MOESM5_ESM.zip › Figure 1/1A/19moGde2WT_CC379-2_TDP43_par-stitch-whole cortex-crop2.tif]

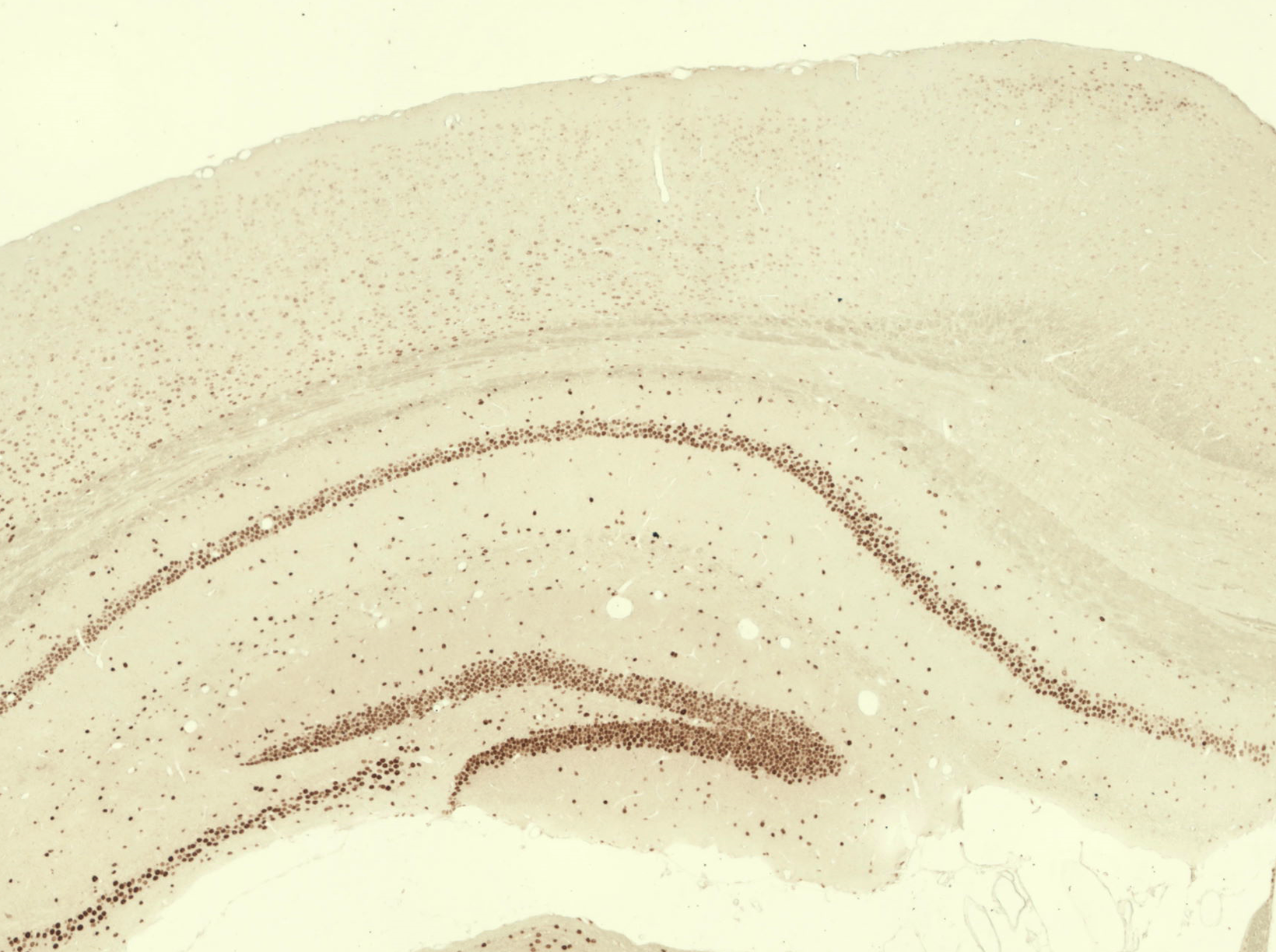

Supplement: Supplementary file 5 — Source data Fig. 1 [file 44318_2024_156_MOESM5_ESM.zip › Figure 1/1B/19moGde2KO_CC380-2_TDP43_par-stitch-whole cortex-crop2.tif]

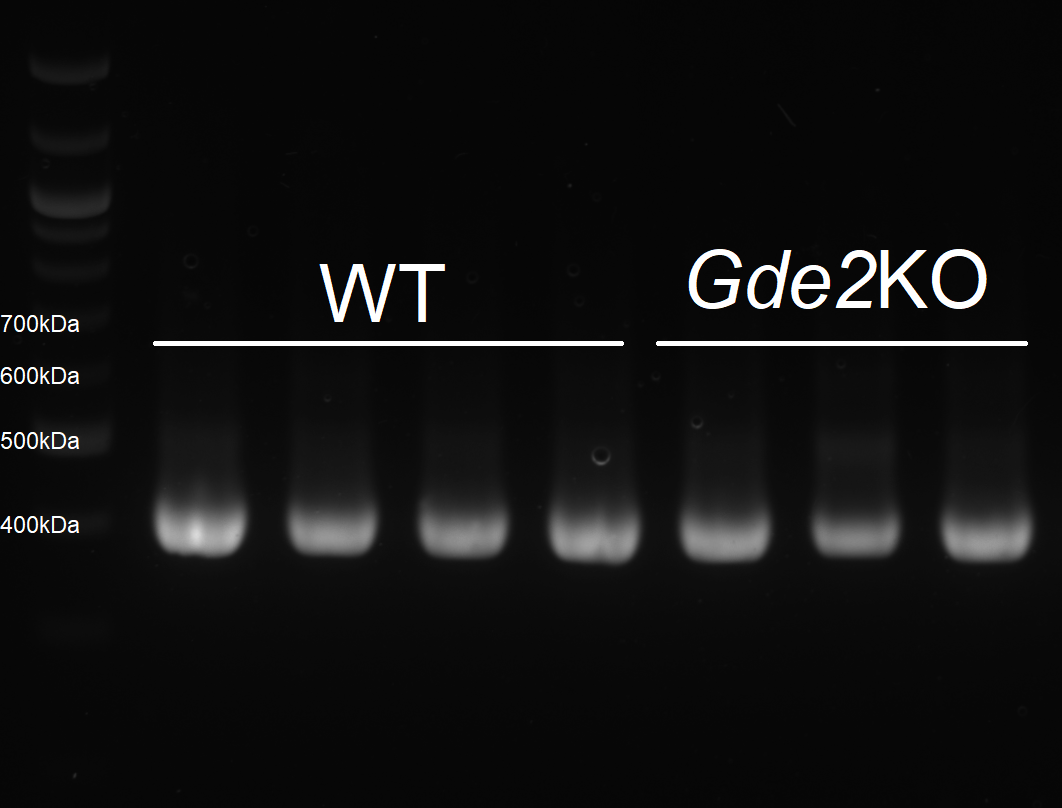

Supplement: Supplementary file 5 — Source data Fig. 1 [file 44318_2024_156_MOESM5_ESM.zip › Figure 1/1G/RT-PCR Camk1g uncropped.tif]

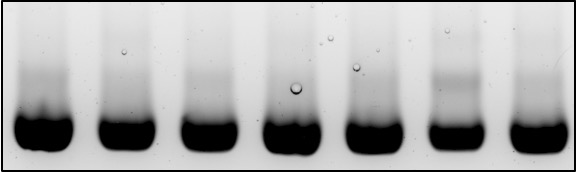

Supplement: Supplementary file 5 — Source data Fig. 1 [file 44318_2024_156_MOESM5_ESM.zip › Figure 1/1G/RT-PCR Camk1g.jpg]

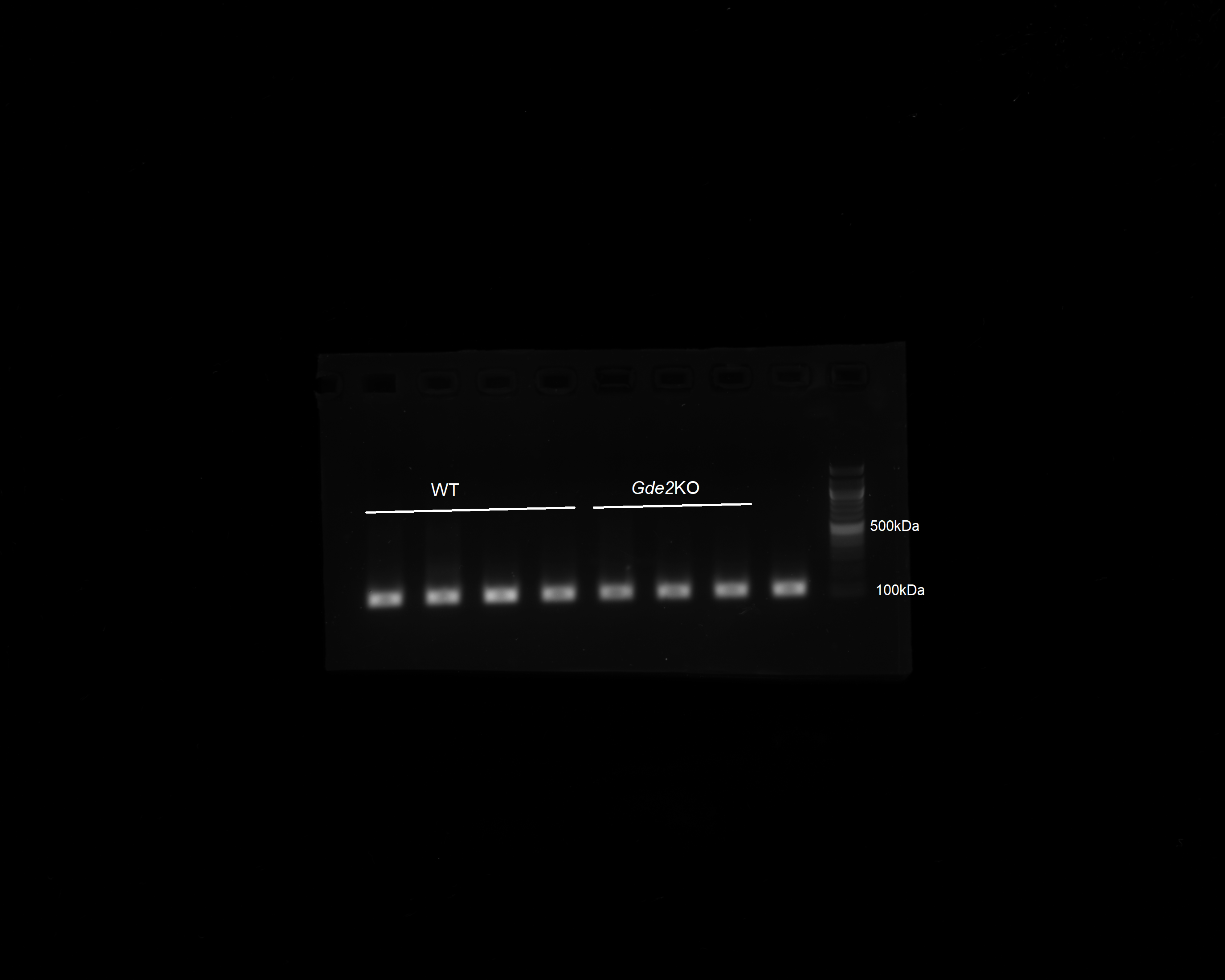

Supplement: Supplementary file 5 — Source data Fig. 1 [file 44318_2024_156_MOESM5_ESM.zip › Figure 1/1G/RT-PCR Gapdh uncropped.tif]

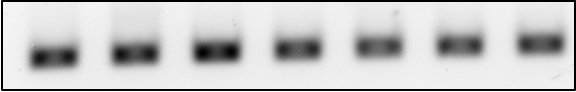

Supplement: Supplementary file 5 — Source data Fig. 1 [file 44318_2024_156_MOESM5_ESM.zip › Figure 1/1G/RT-PCR Gapdh.jpg]

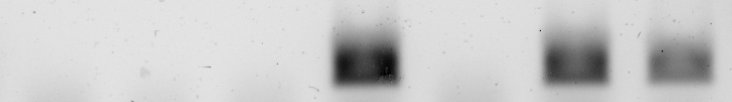

Supplement: Supplementary file 5 — Source data Fig. 1 [file 44318_2024_156_MOESM5_ESM.zip › Figure 1/1I/RT-PCR Synj2bp.jpg]

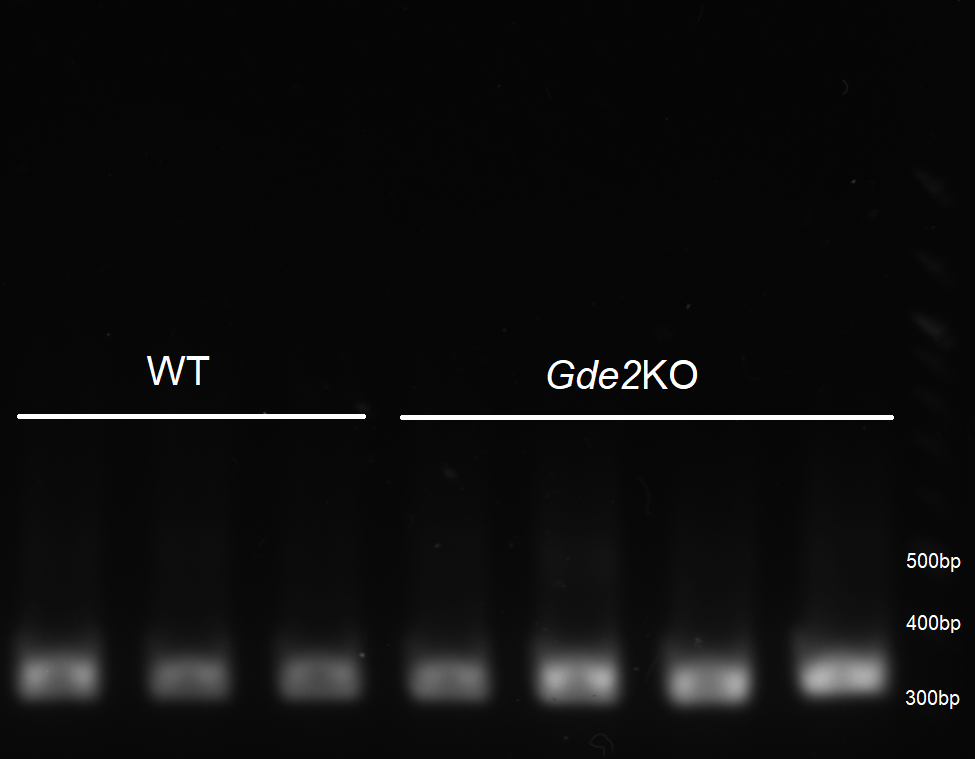

Supplement: Supplementary file 5 — Source data Fig. 1 [file 44318_2024_156_MOESM5_ESM.zip › Figure 1/1I/RT-PCR Tecpr1 uncropped.tif]

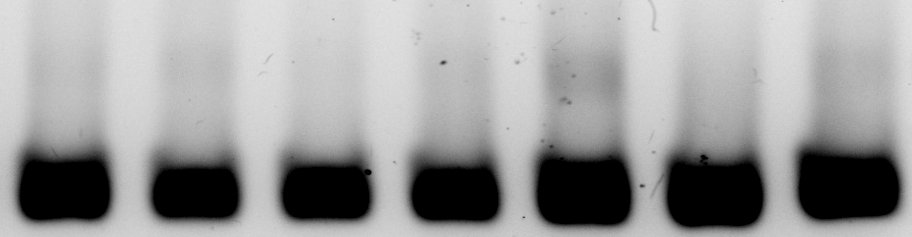

Supplement: Supplementary file 5 — Source data Fig. 1 [file 44318_2024_156_MOESM5_ESM.zip › Figure 1/1I/RT-PCR Tecpr1.jpg]

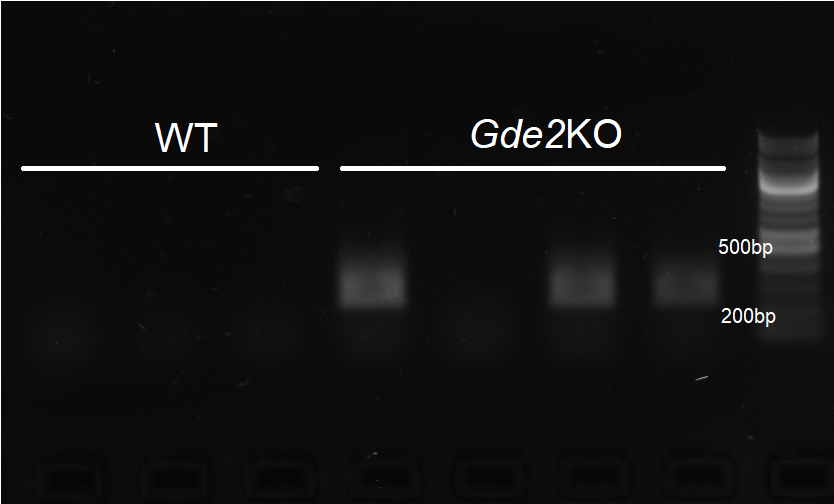

Supplement: Supplementary file 5 — Source data Fig. 1 [file 44318_2024_156_MOESM5_ESM.zip › Figure 1/1I/RT-PCR uncropped Synj2bp CE.tif]

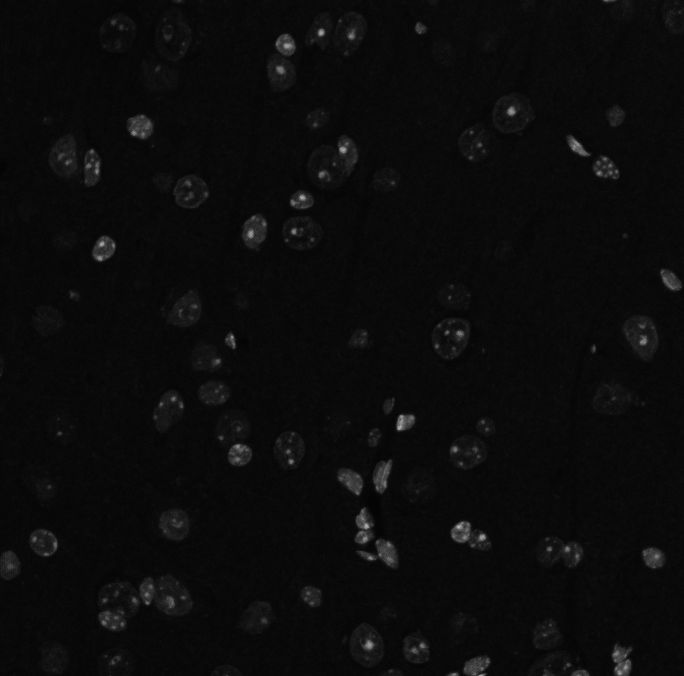

Supplement: Supplementary file 6 — Source data Fig. 2 [file 44318_2024_156_MOESM6_ESM.zip › Figure 2/2I/SS019-3-5_Gde2WT.tif]

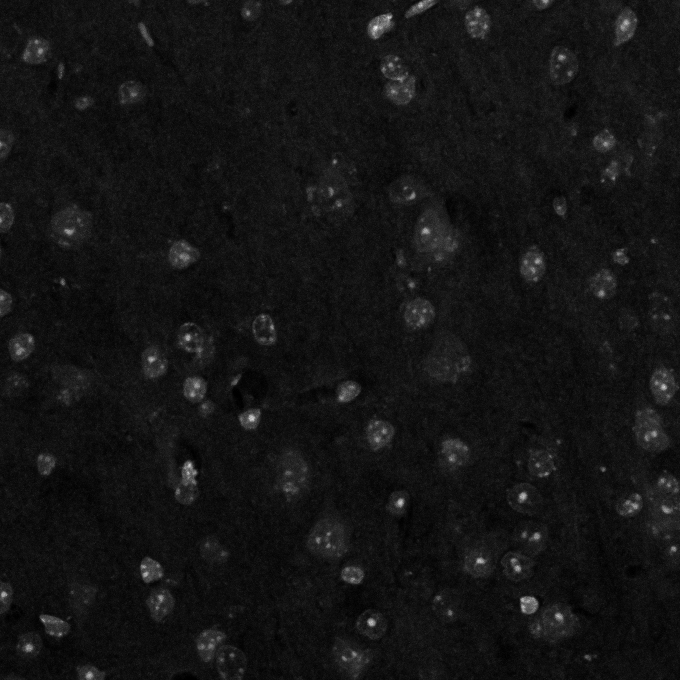

Supplement: Supplementary file 6 — Source data Fig. 2 [file 44318_2024_156_MOESM6_ESM.zip › Figure 2/2J/SS009-1-5_Gde2KO.tif]

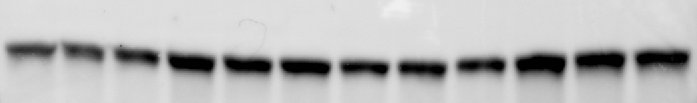

Supplement: Supplementary file 6 — Source data Fig. 2 [file 44318_2024_156_MOESM6_ESM.zip › Figure 2/2P/actin-gray crop.jpg]

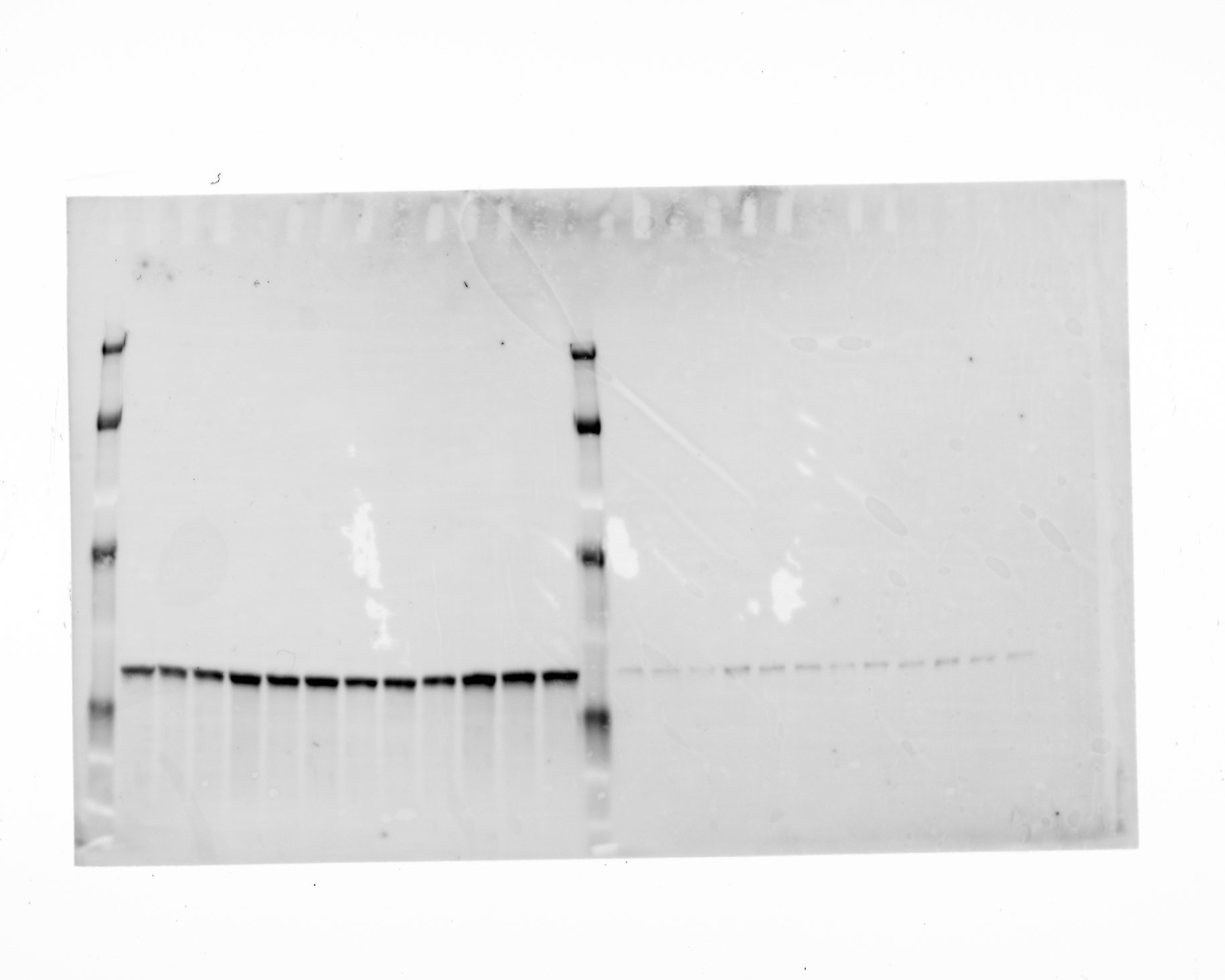

Supplement: Supplementary file 6 — Source data Fig. 2 [file 44318_2024_156_MOESM6_ESM.zip › Figure 2/2P/actin-gray.jpg]

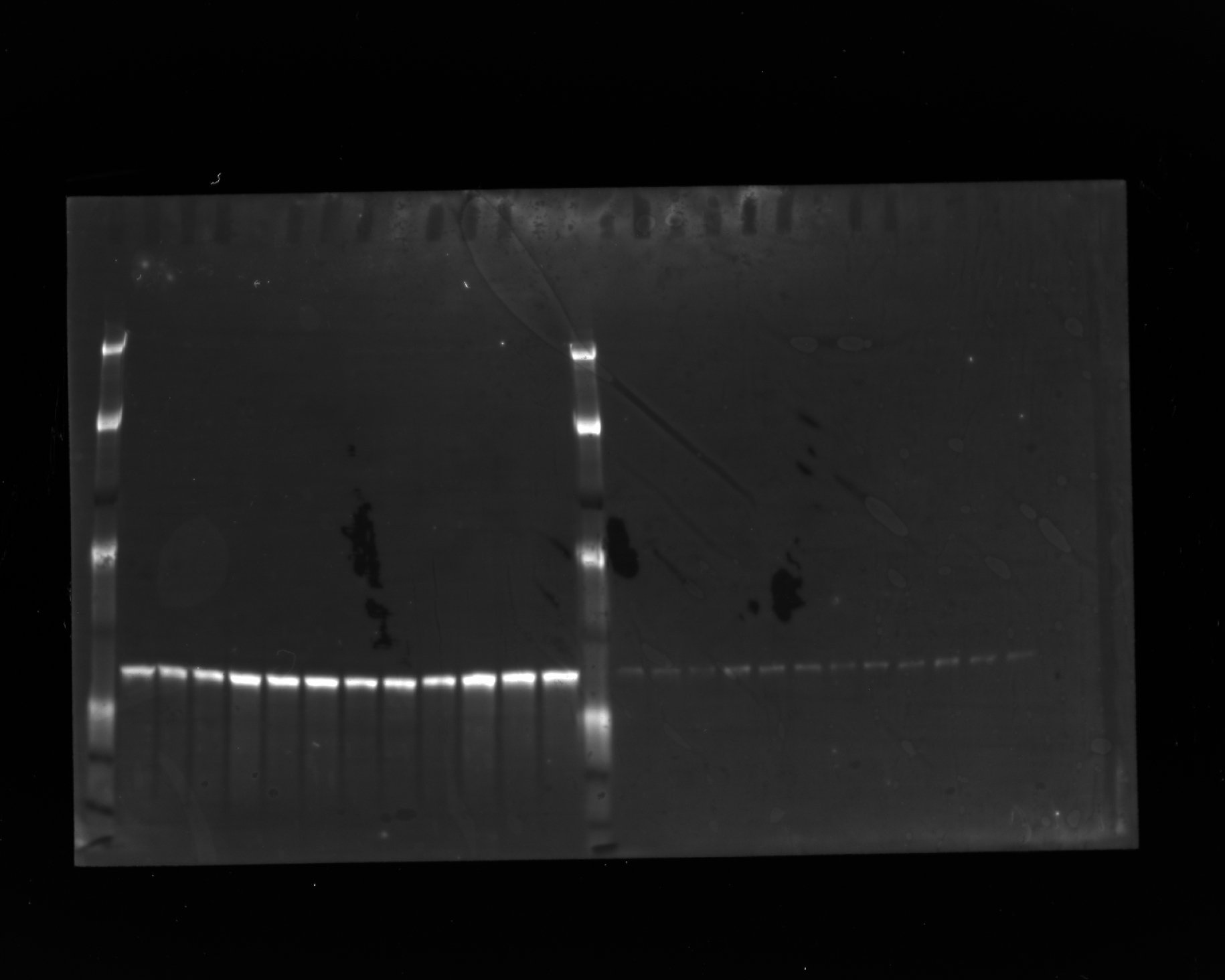

Supplement: Supplementary file 6 — Source data Fig. 2 [file 44318_2024_156_MOESM6_ESM.zip › Figure 2/2P/actin-inverted.jpg]

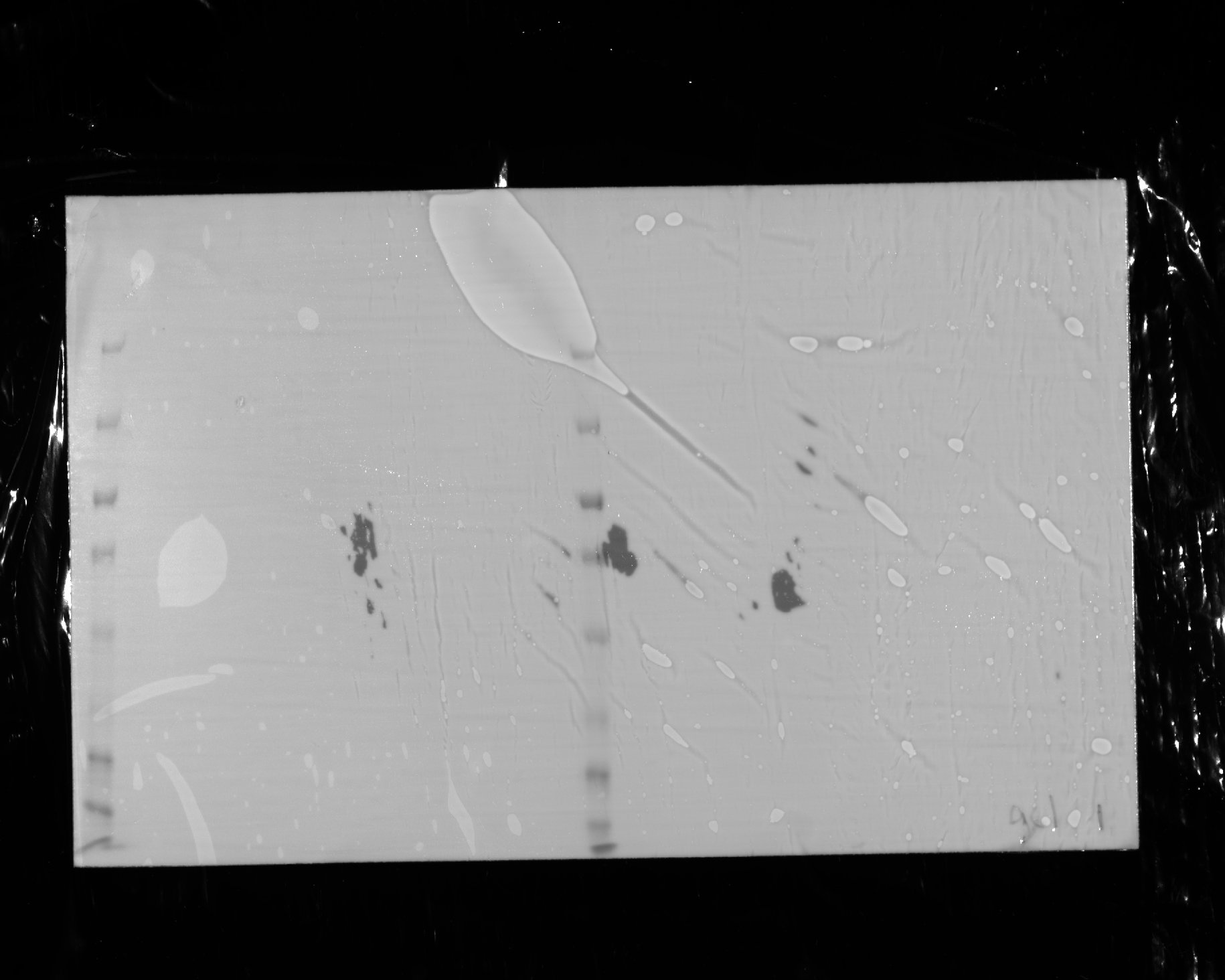

Supplement: Supplementary file 6 — Source data Fig. 2 [file 44318_2024_156_MOESM6_ESM.zip › Figure 2/2P/nup98+actin-white.jpg]

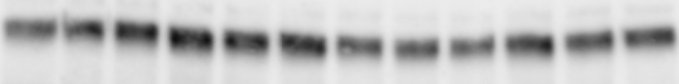

Supplement: Supplementary file 6 — Source data Fig. 2 [file 44318_2024_156_MOESM6_ESM.zip › Figure 2/2P/nup98-gray crop.jpg]

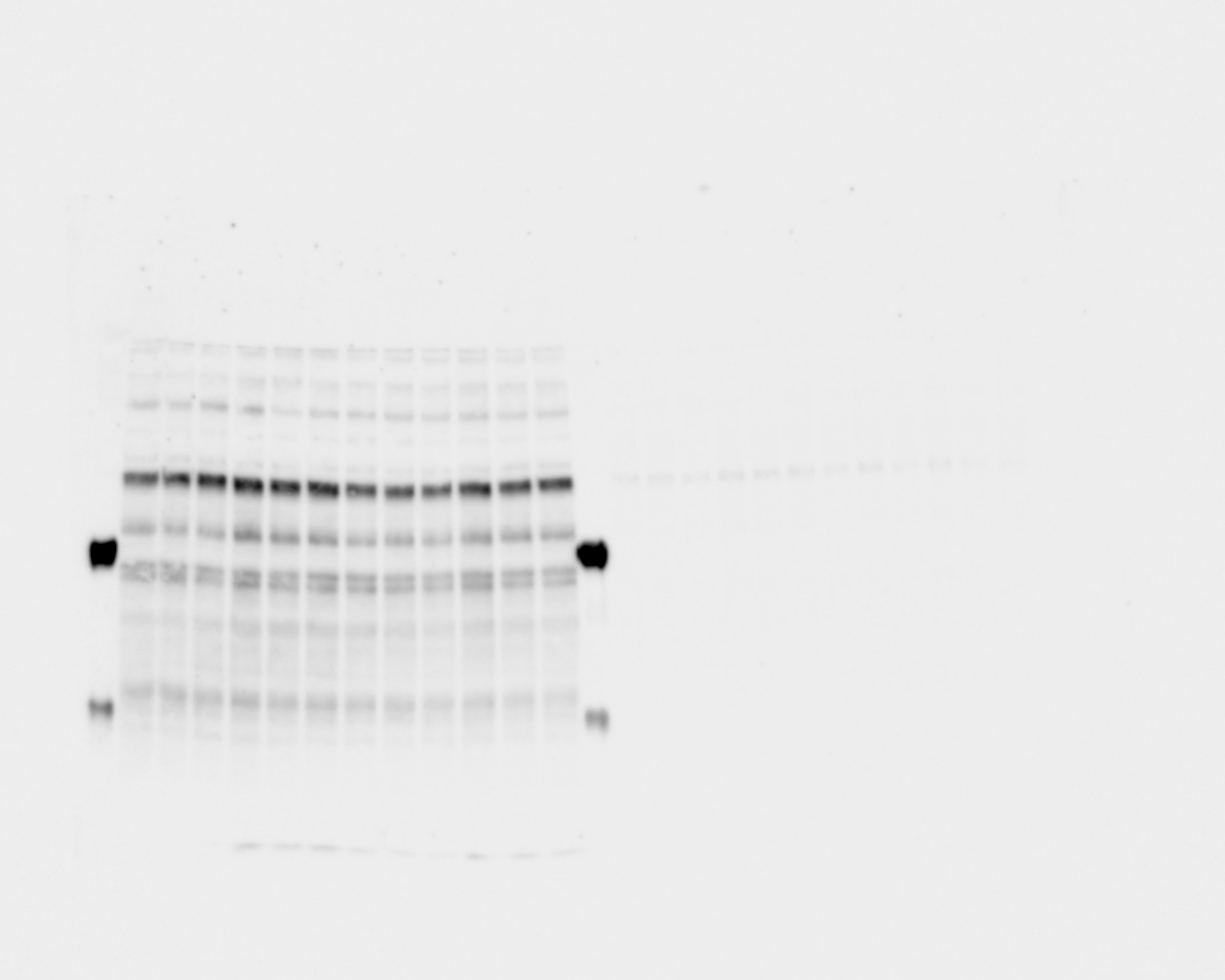

Supplement: Supplementary file 6 — Source data Fig. 2 [file 44318_2024_156_MOESM6_ESM.zip › Figure 2/2P/nup98-gray.jpg]

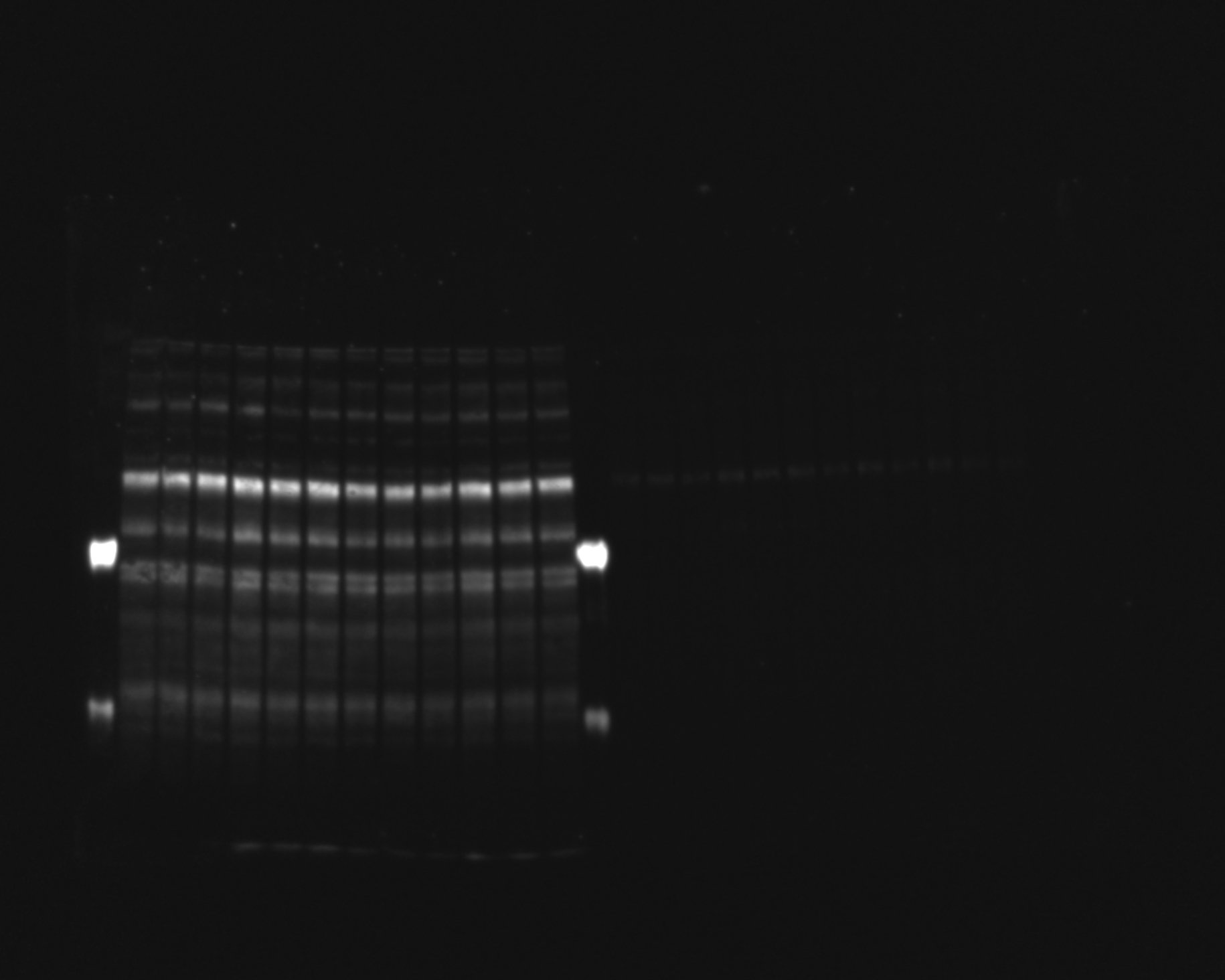

Supplement: Supplementary file 6 — Source data Fig. 2 [file 44318_2024_156_MOESM6_ESM.zip › Figure 2/2P/nup98-inverted.jpg]

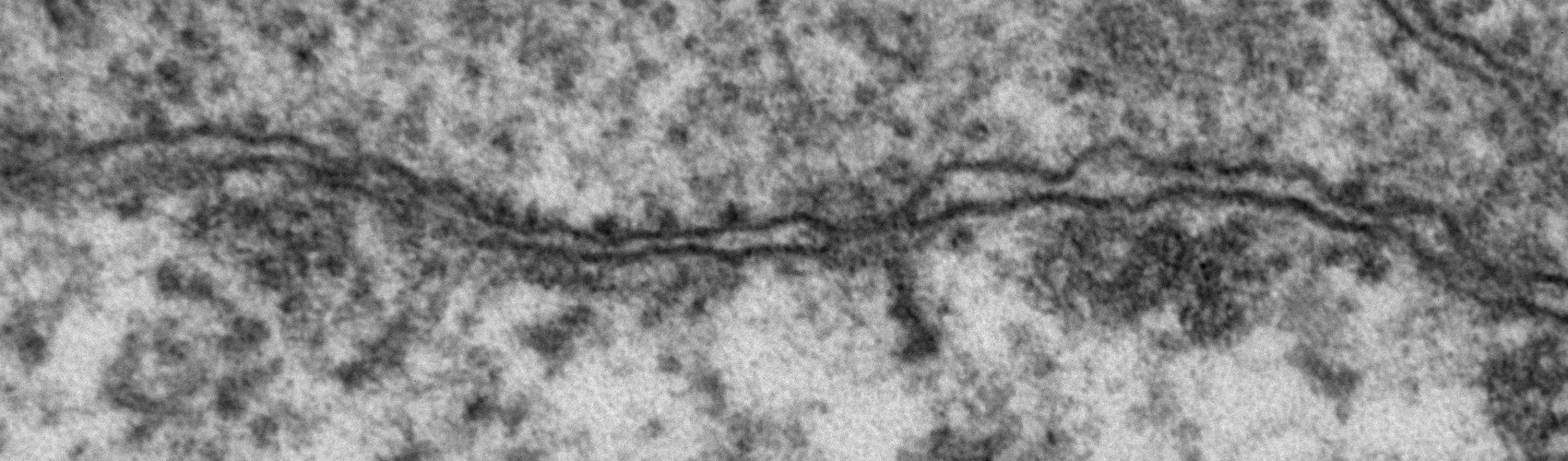

Supplement: Supplementary file 6 — Source data Fig. 2 [file 44318_2024_156_MOESM6_ESM.zip › Figure 2/2T/cropS1 113612 D1_015_16.tif]

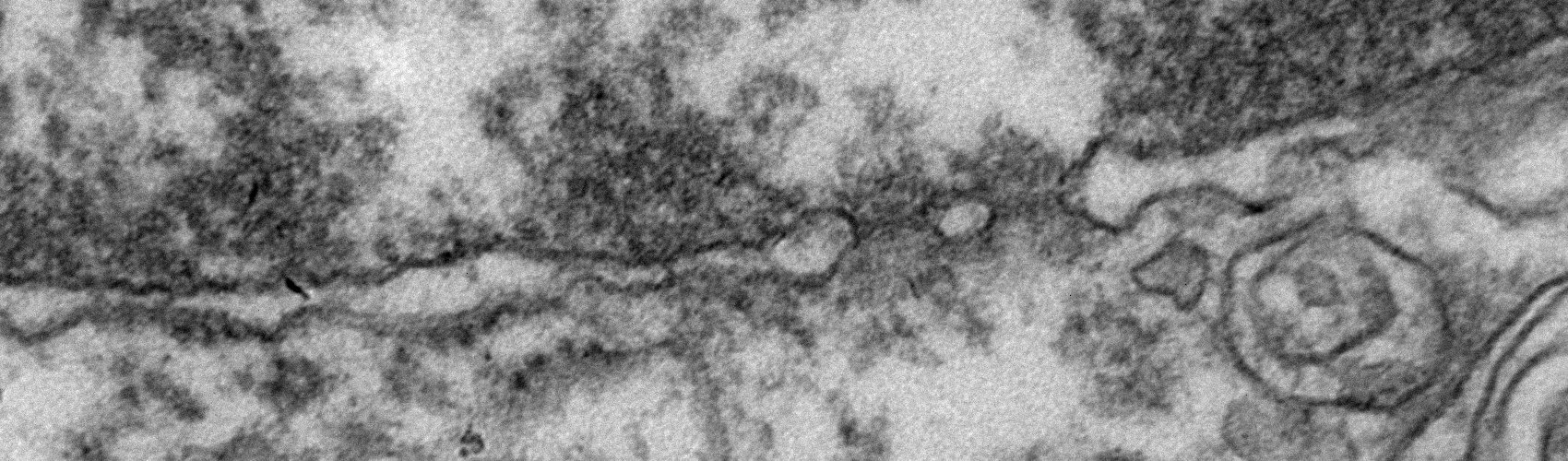

Supplement: Supplementary file 6 — Source data Fig. 2 [file 44318_2024_156_MOESM6_ESM.zip › Figure 2/2T/cropS4 113612 G1_008_16.tif]

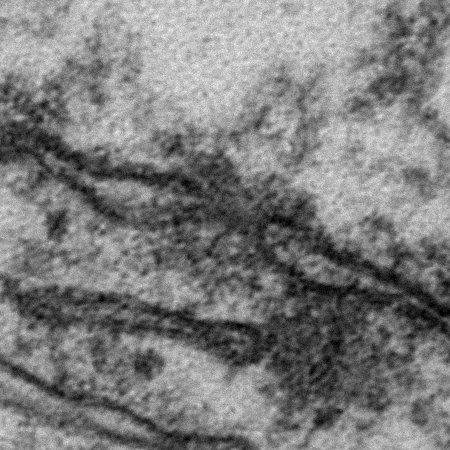

Supplement: Supplementary file 6 — Source data Fig. 2 [file 44318_2024_156_MOESM6_ESM.zip › Figure 2/2T/NPC1-S2 113612 E1_004_16.tif]

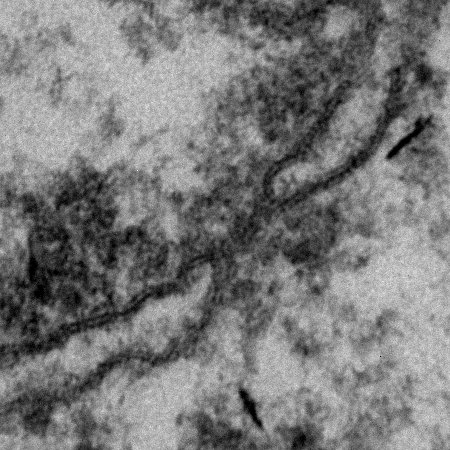

Supplement: Supplementary file 6 — Source data Fig. 2 [file 44318_2024_156_MOESM6_ESM.zip › Figure 2/2T/NPC1-S4-11362-G3_013.tif]

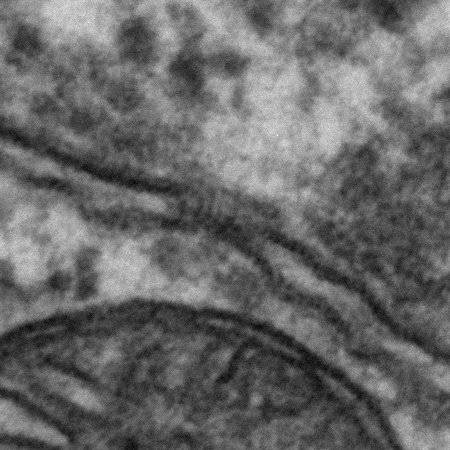

Supplement: Supplementary file 6 — Source data Fig. 2 [file 44318_2024_156_MOESM6_ESM.zip › Figure 2/2T/NPC2-S1 11362 D3_075_16.tif]

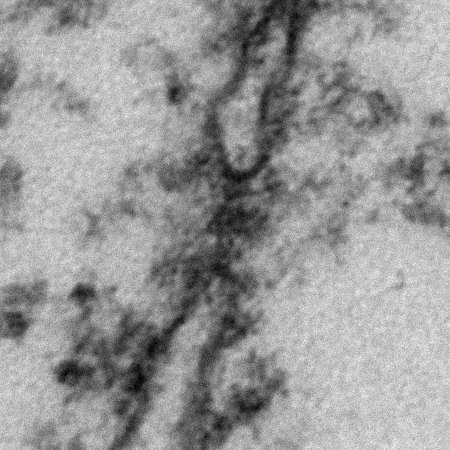

Supplement: Supplementary file 6 — Source data Fig. 2 [file 44318_2024_156_MOESM6_ESM.zip › Figure 2/2T/NPC2-S3 113612 F1_030_16.tif]

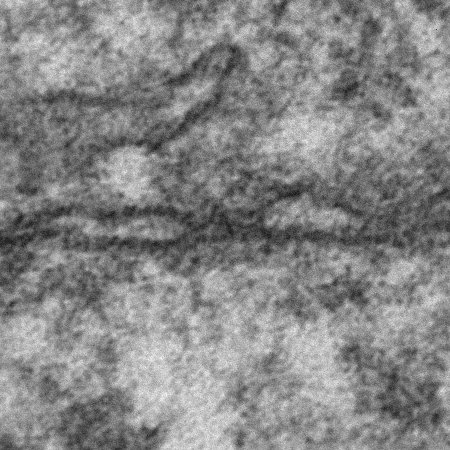

Supplement: Supplementary file 6 — Source data Fig. 2 [file 44318_2024_156_MOESM6_ESM.zip › Figure 2/2T/NPC3-S1 113612 D1_007_16-1.tif]

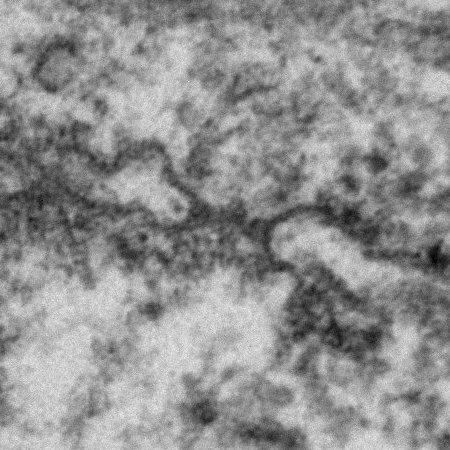

Supplement: Supplementary file 6 — Source data Fig. 2 [file 44318_2024_156_MOESM6_ESM.zip › Figure 2/2T/NPC3-S4-11362-G3_018.tif]

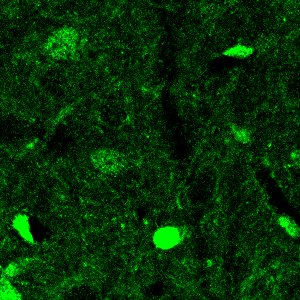

Supplement: Supplementary file 7 — Source data Fig. 3 [file 44318_2024_156_MOESM7_ESM.zip › Figure 3/3A-b''/3a'.jpg]

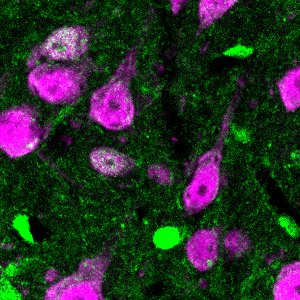

Supplement: Supplementary file 7 — Source data Fig. 3 [file 44318_2024_156_MOESM7_ESM.zip › Figure 3/3A-b''/3a''.jpg]

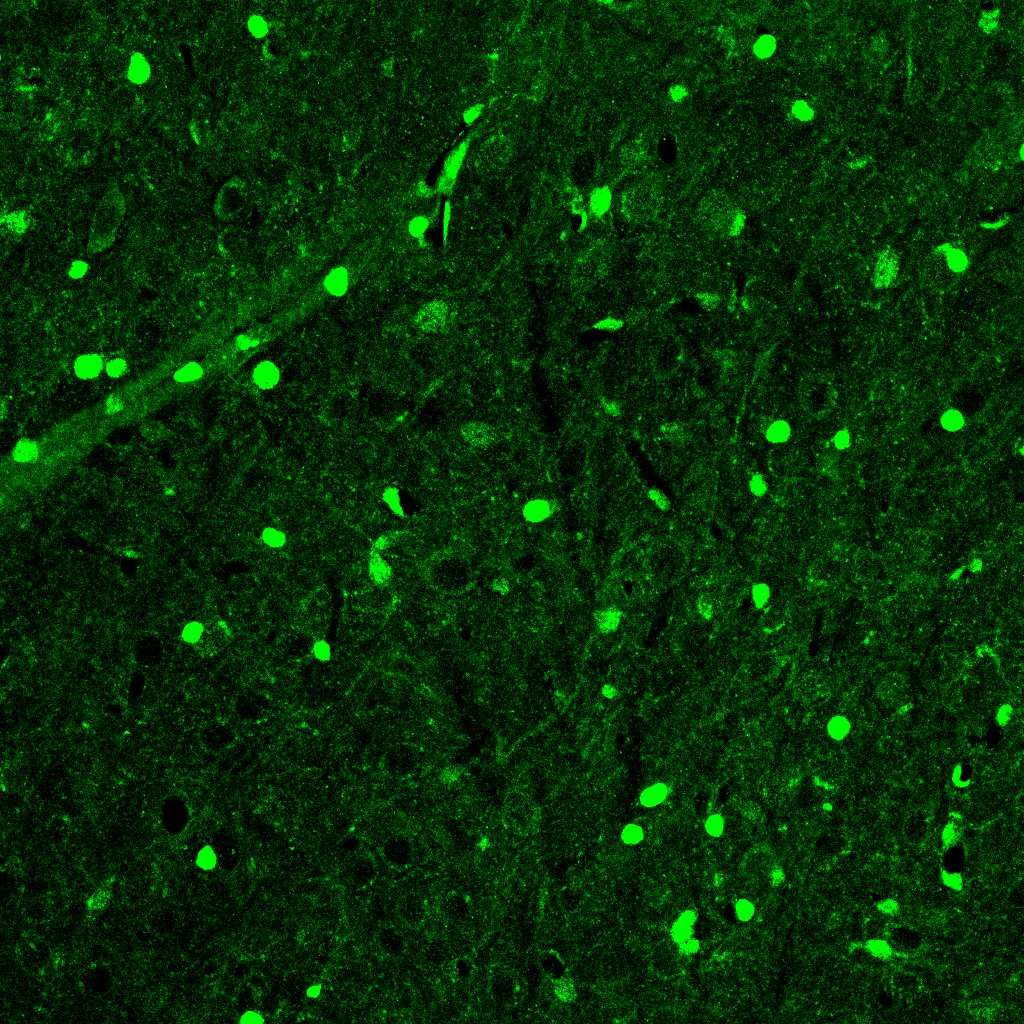

Supplement: Supplementary file 7 — Source data Fig. 3 [file 44318_2024_156_MOESM7_ESM.zip › Figure 3/3A-b''/3A.jpg]

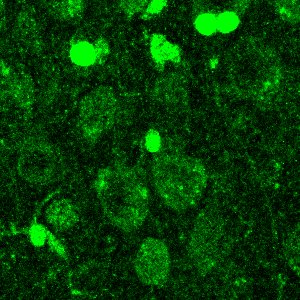

Supplement: Supplementary file 7 — Source data Fig. 3 [file 44318_2024_156_MOESM7_ESM.zip › Figure 3/3A-b''/3b'.jpg]

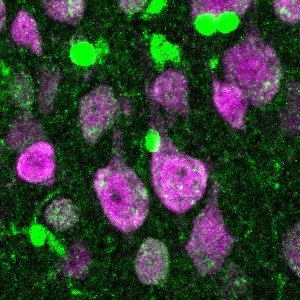

Supplement: Supplementary file 7 — Source data Fig. 3 [file 44318_2024_156_MOESM7_ESM.zip › Figure 3/3A-b''/3b''.jpg]

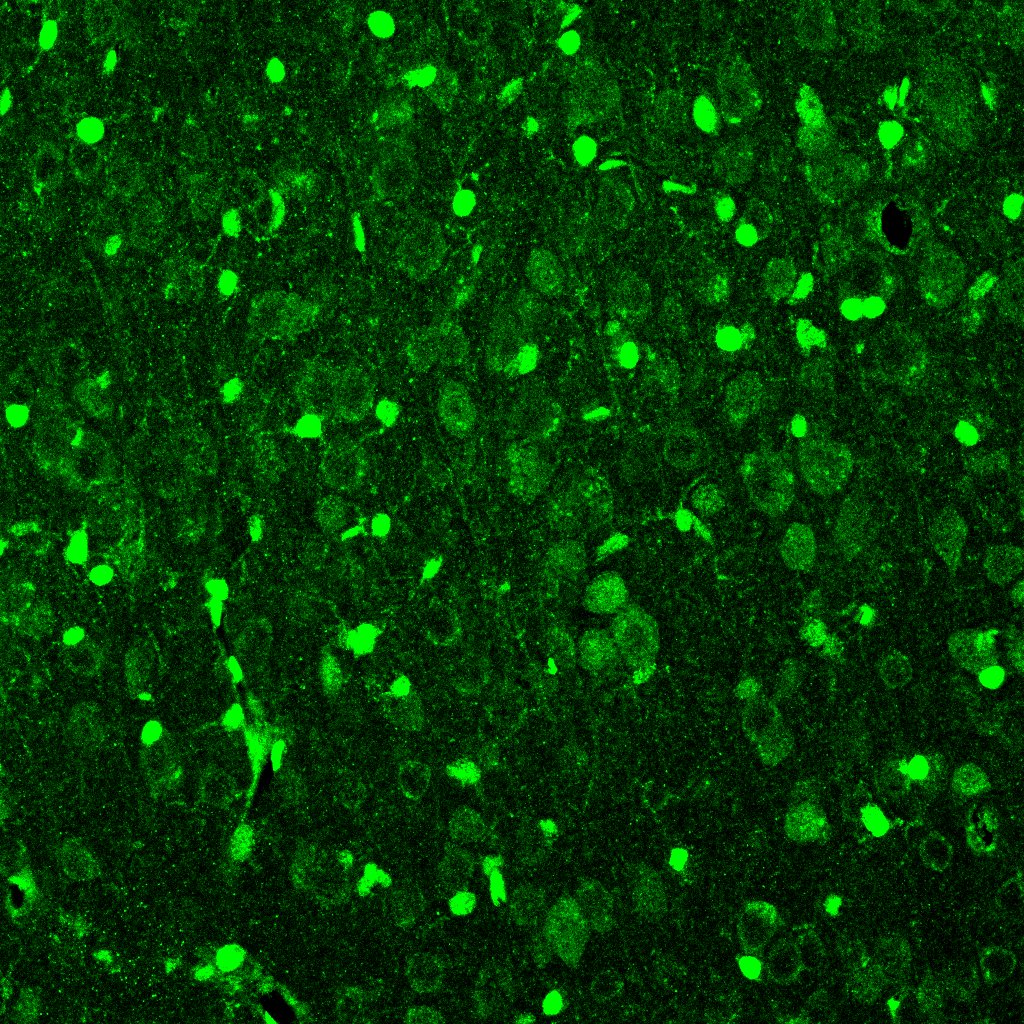

Supplement: Supplementary file 7 — Source data Fig. 3 [file 44318_2024_156_MOESM7_ESM.zip › Figure 3/3A-b''/3B.jpg]

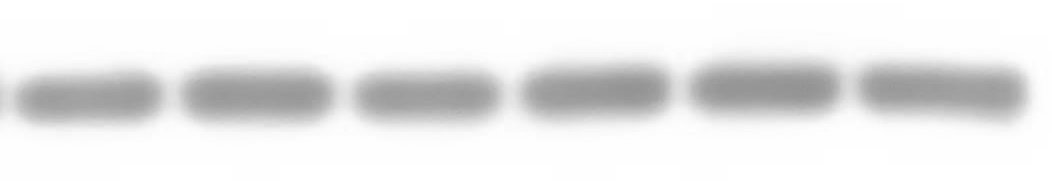

Supplement: Supplementary file 7 — Source data Fig. 3 [file 44318_2024_156_MOESM7_ESM.zip › Figure 3/3D/alpha-tubulin crop.jpg]

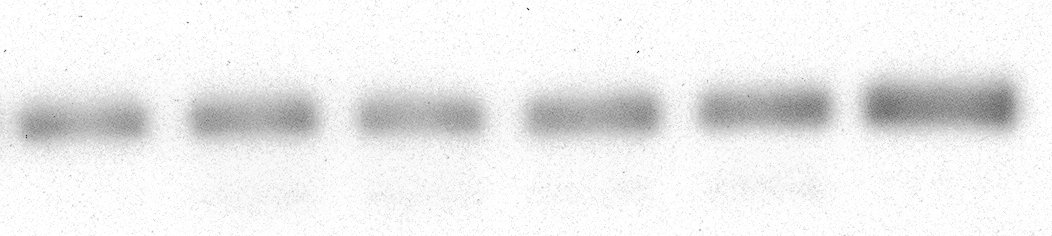

Supplement: Supplementary file 7 — Source data Fig. 3 [file 44318_2024_156_MOESM7_ESM.zip › Figure 3/3D/beta-catenin crop.jpg]

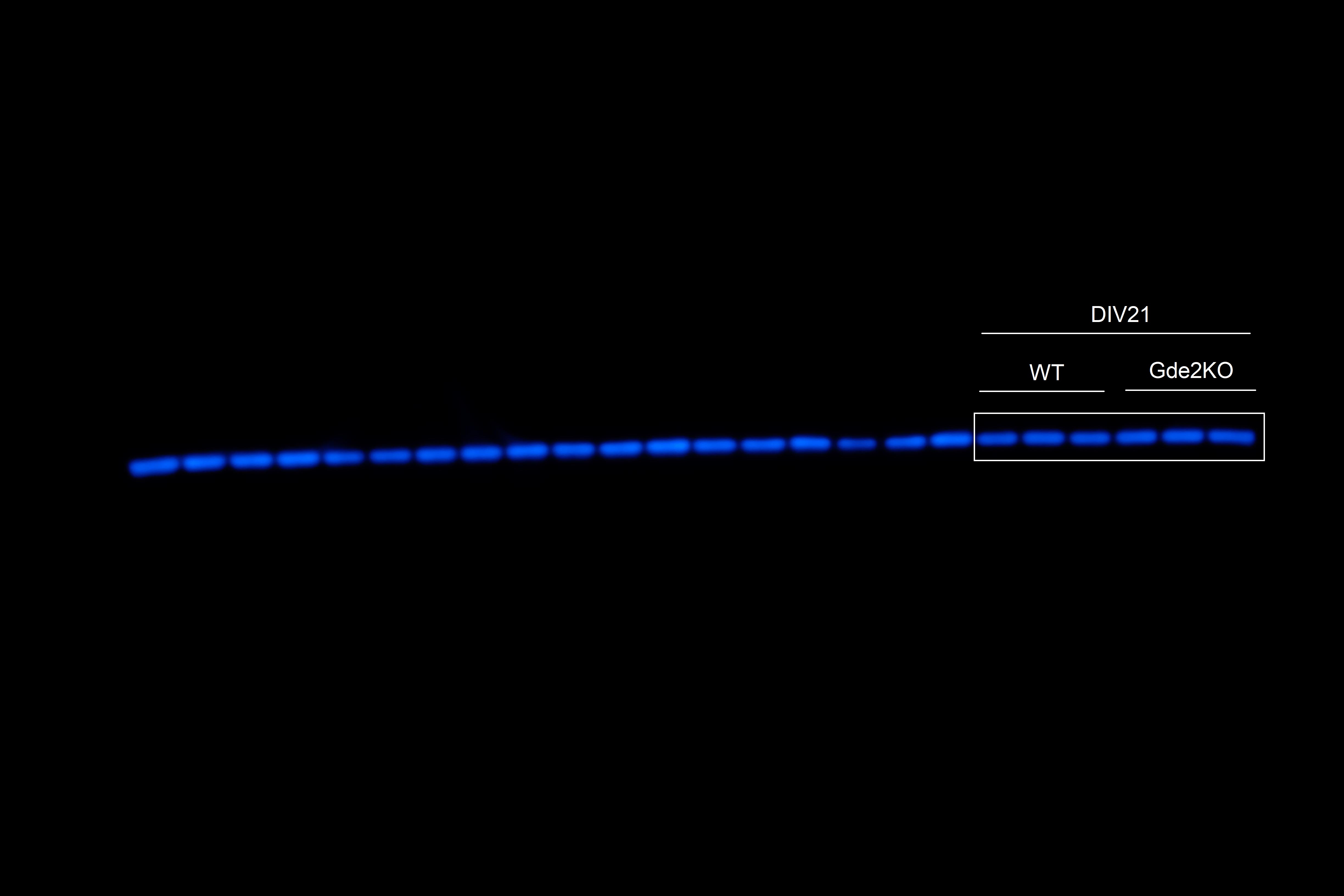

Supplement: Supplementary file 7 — Source data Fig. 3 [file 44318_2024_156_MOESM7_ESM.zip › Figure 3/3D/western alpha-tubulin.JPG]

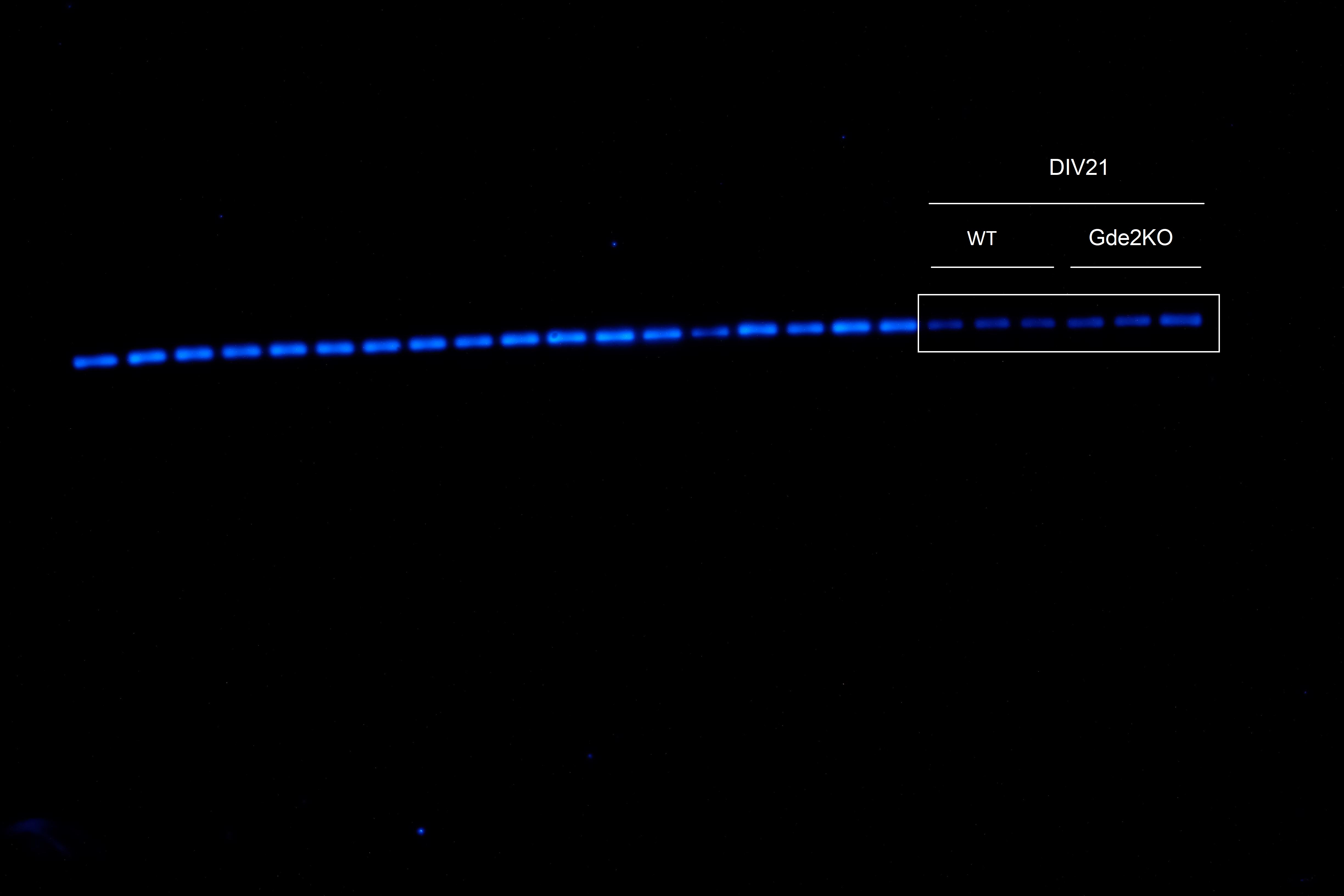

Supplement: Supplementary file 7 — Source data Fig. 3 [file 44318_2024_156_MOESM7_ESM.zip › Figure 3/3D/western beta-catenin.JPG]

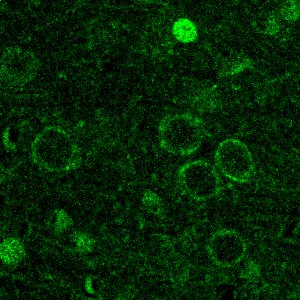

Supplement: Supplementary file 7 — Source data Fig. 3 [file 44318_2024_156_MOESM7_ESM.zip › Figure 3/3F-K/3F.jpg]

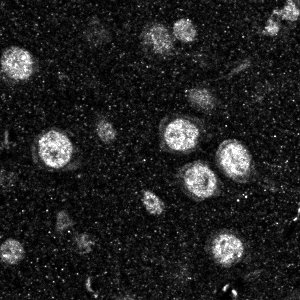

Supplement: Supplementary file 7 — Source data Fig. 3 [file 44318_2024_156_MOESM7_ESM.zip › Figure 3/3F-K/3G.jpg]

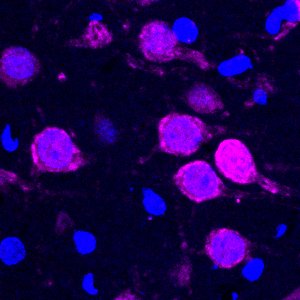

Supplement: Supplementary file 7 — Source data Fig. 3 [file 44318_2024_156_MOESM7_ESM.zip › Figure 3/3F-K/3H.jpg]

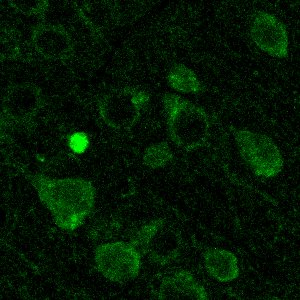

Supplement: Supplementary file 7 — Source data Fig. 3 [file 44318_2024_156_MOESM7_ESM.zip › Figure 3/3F-K/3I.jpg]

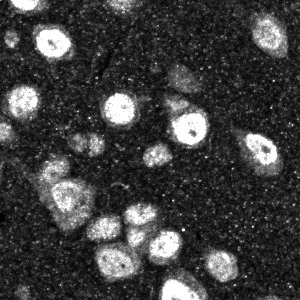

Supplement: Supplementary file 7 — Source data Fig. 3 [file 44318_2024_156_MOESM7_ESM.zip › Figure 3/3F-K/3J.jpg]

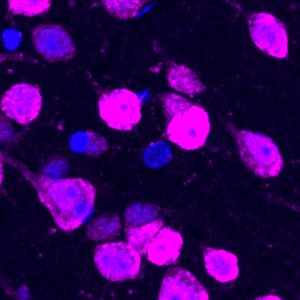

Supplement: Supplementary file 7 — Source data Fig. 3 [file 44318_2024_156_MOESM7_ESM.zip › Figure 3/3F-K/3K.jpg]

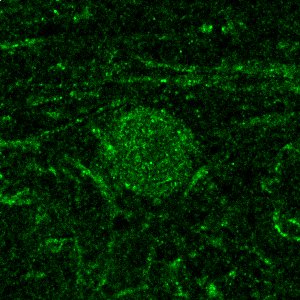

Supplement: Supplementary file 8 — Source data Fig. 4 [file 44318_2024_156_MOESM8_ESM.zip › Figure 4/4A-D/4A.jpg]

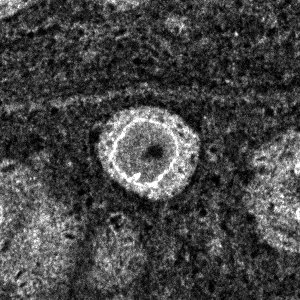

Supplement: Supplementary file 8 — Source data Fig. 4 [file 44318_2024_156_MOESM8_ESM.zip › Figure 4/4A-D/4B.jpg]

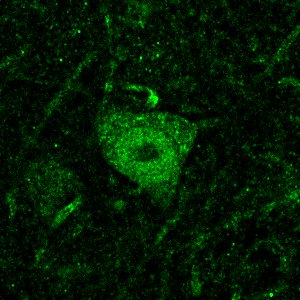

Supplement: Supplementary file 8 — Source data Fig. 4 [file 44318_2024_156_MOESM8_ESM.zip › Figure 4/4A-D/4C.jpg]

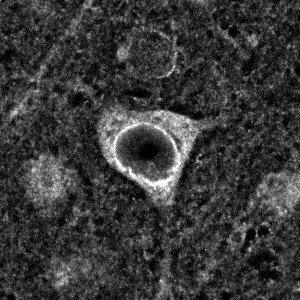

Supplement: Supplementary file 8 — Source data Fig. 4 [file 44318_2024_156_MOESM8_ESM.zip › Figure 4/4A-D/4D.jpg]

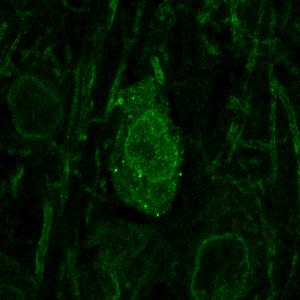

Supplement: Supplementary file 8 — Source data Fig. 4 [file 44318_2024_156_MOESM8_ESM.zip › Figure 4/4E-H/4E.jpg]

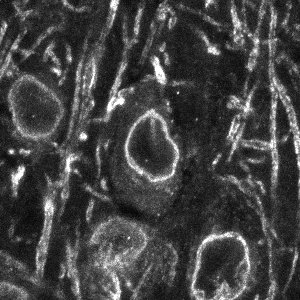

Supplement: Supplementary file 8 — Source data Fig. 4 [file 44318_2024_156_MOESM8_ESM.zip › Figure 4/4E-H/4F.jpg]

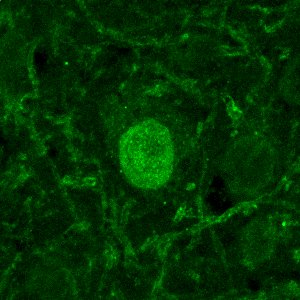

Supplement: Supplementary file 8 — Source data Fig. 4 [file 44318_2024_156_MOESM8_ESM.zip › Figure 4/4E-H/4G.jpg]

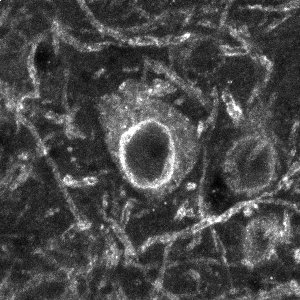

Supplement: Supplementary file 8 — Source data Fig. 4 [file 44318_2024_156_MOESM8_ESM.zip › Figure 4/4E-H/4H.jpg]

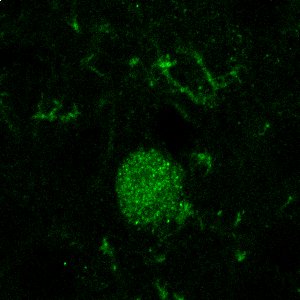

Supplement: Supplementary file 8 — Source data Fig. 4 [file 44318_2024_156_MOESM8_ESM.zip › Figure 4/4I-L/4I.jpg]

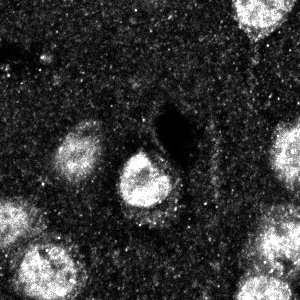

Supplement: Supplementary file 8 — Source data Fig. 4 [file 44318_2024_156_MOESM8_ESM.zip › Figure 4/4I-L/4J.jpg]

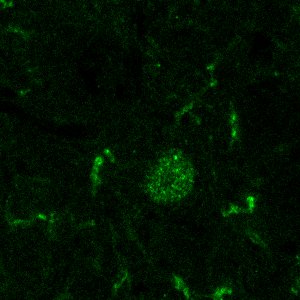

Supplement: Supplementary file 8 — Source data Fig. 4 [file 44318_2024_156_MOESM8_ESM.zip › Figure 4/4I-L/4K.jpg]

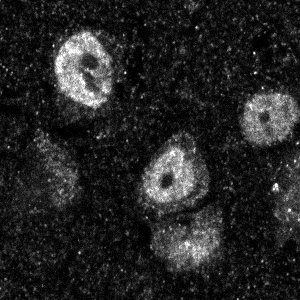

Supplement: Supplementary file 8 — Source data Fig. 4 [file 44318_2024_156_MOESM8_ESM.zip › Figure 4/4I-L/4L.jpg]

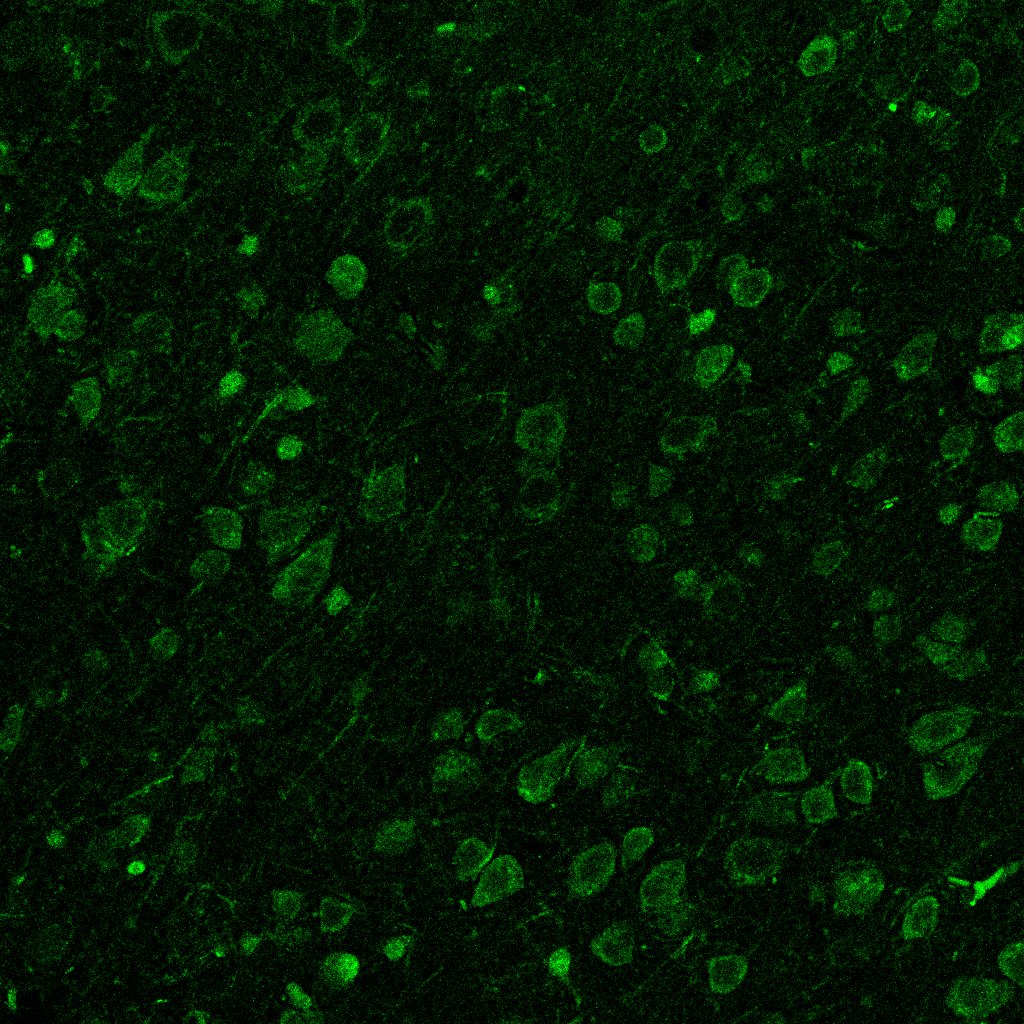

Supplement: Supplementary file 8 — Source data Fig. 4 [file 44318_2024_156_MOESM8_ESM.zip › Figure 4/4Q-R/4Q.jpg]

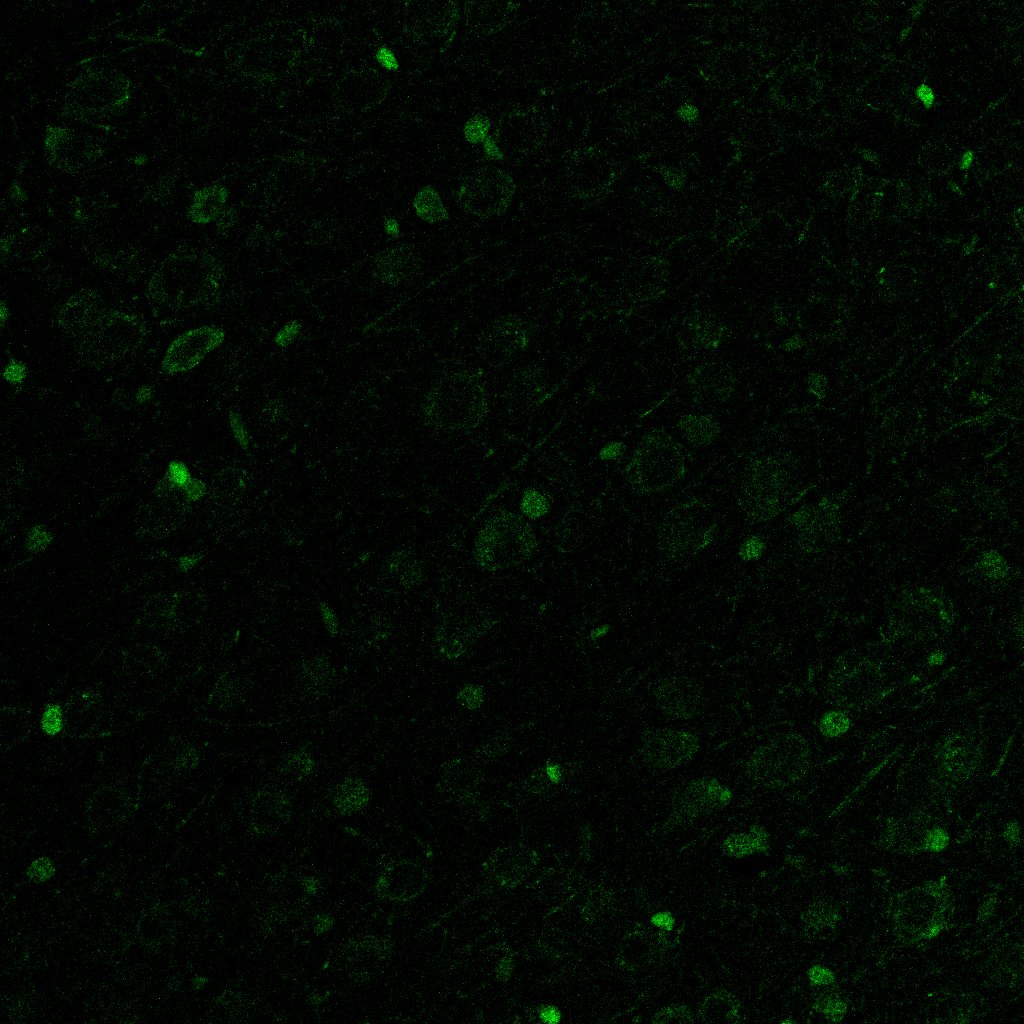

Supplement: Supplementary file 8 — Source data Fig. 4 [file 44318_2024_156_MOESM8_ESM.zip › Figure 4/4Q-R/4R.jpg]

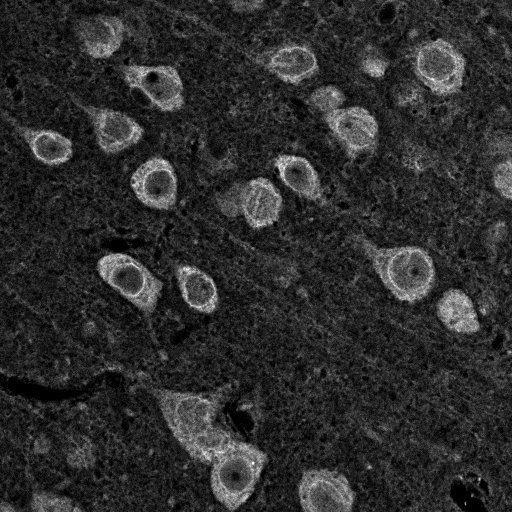

Supplement: Supplementary file 8 — Source data Fig. 4 [file 44318_2024_156_MOESM8_ESM.zip › Figure 4/4U-V/4U.jpg]

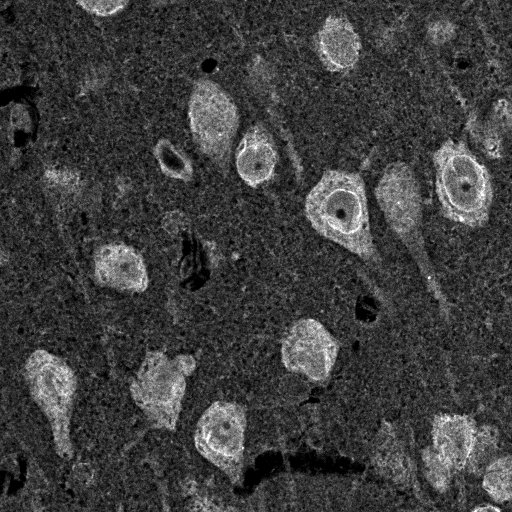

Supplement: Supplementary file 8 — Source data Fig. 4 [file 44318_2024_156_MOESM8_ESM.zip › Figure 4/4U-V/4V.jpg]

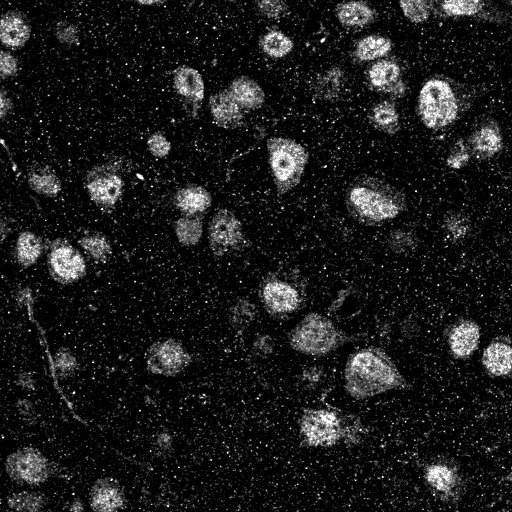

Supplement: Supplementary file 8 — Source data Fig. 4 [file 44318_2024_156_MOESM8_ESM.zip › Figure 4/4W-X/4W.jpg]

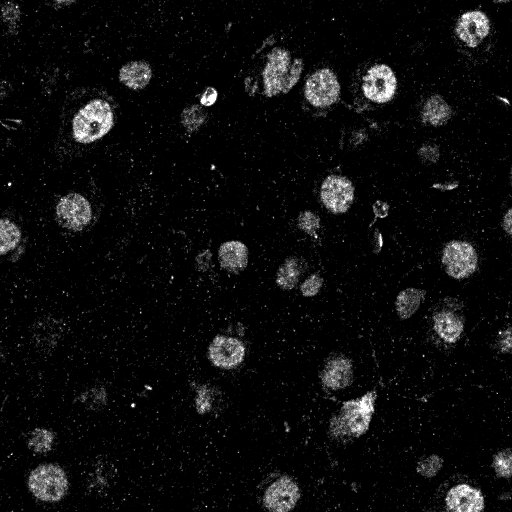

Supplement: Supplementary file 8 — Source data Fig. 4 [file 44318_2024_156_MOESM8_ESM.zip › Figure 4/4W-X/4X.jpg]

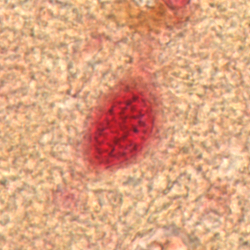

Supplement: Supplementary file 9 — Source data Fig. 5 [file 44318_2024_156_MOESM9_ESM.zip › Figure 5/5A/39_GDE2.TDP43_15_crop.tif]

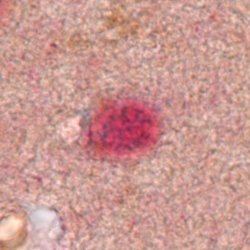

Supplement: Supplementary file 9 — Source data Fig. 5 [file 44318_2024_156_MOESM9_ESM.zip › Figure 5/5A/40_GDE2.TDP43_13-crop.tif]

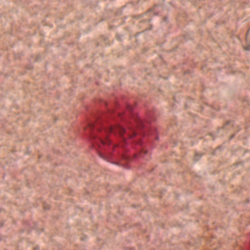

Supplement: Supplementary file 9 — Source data Fig. 5 [file 44318_2024_156_MOESM9_ESM.zip › Figure 5/5A/44_GDE2.TDP43_05-crop.tif]

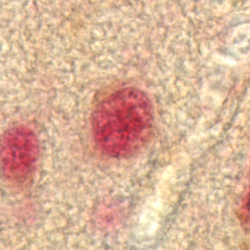

Supplement: Supplementary file 9 — Source data Fig. 5 [file 44318_2024_156_MOESM9_ESM.zip › Figure 5/5A/70_GDE2.TDP43_09-crop.tif]

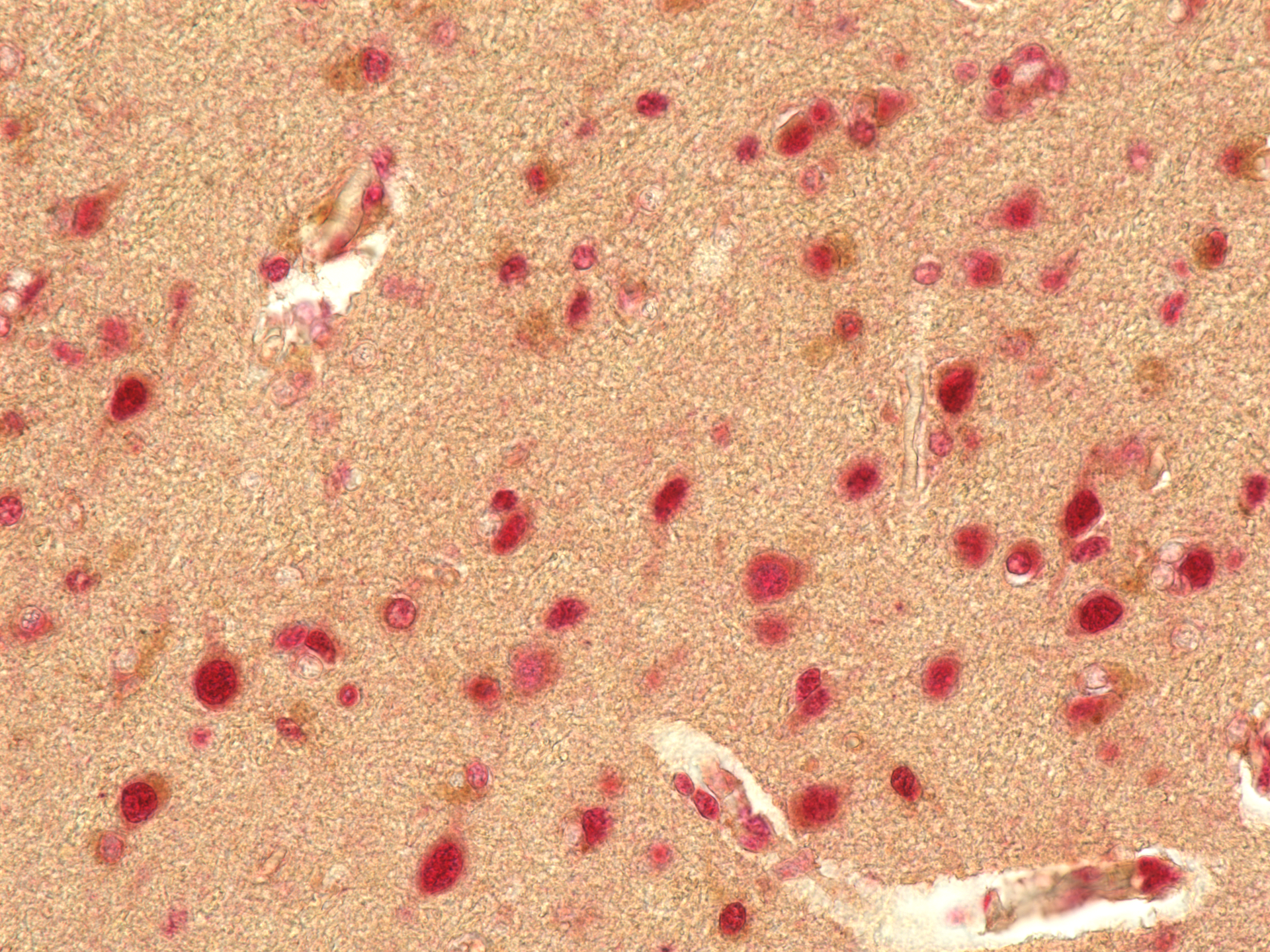

Supplement: Supplementary file 9 — Source data Fig. 5 [file 44318_2024_156_MOESM9_ESM.zip › Figure 5/5A/ctrl_GDE2.TDP43_15.tif]

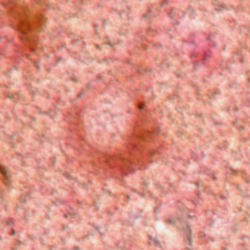

Supplement: Supplementary file 9 — Source data Fig. 5 [file 44318_2024_156_MOESM9_ESM.zip › Figure 5/5B/120_GDE2.TDP43_06-crop.tif]

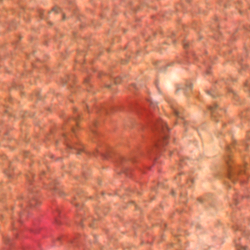

Supplement: Supplementary file 9 — Source data Fig. 5 [file 44318_2024_156_MOESM9_ESM.zip › Figure 5/5B/73_GDE2.TDP43_06_crop.tif]

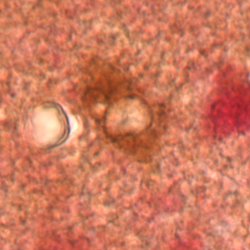

Supplement: Supplementary file 9 — Source data Fig. 5 [file 44318_2024_156_MOESM9_ESM.zip › Figure 5/5B/73_GDE2.TDP43_08-crop.tif]

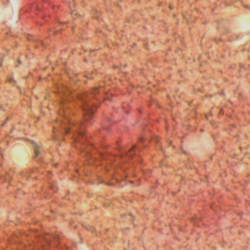

Supplement: Supplementary file 9 — Source data Fig. 5 [file 44318_2024_156_MOESM9_ESM.zip › Figure 5/5B/88-GDE2.TDP43_10-crop.tif]

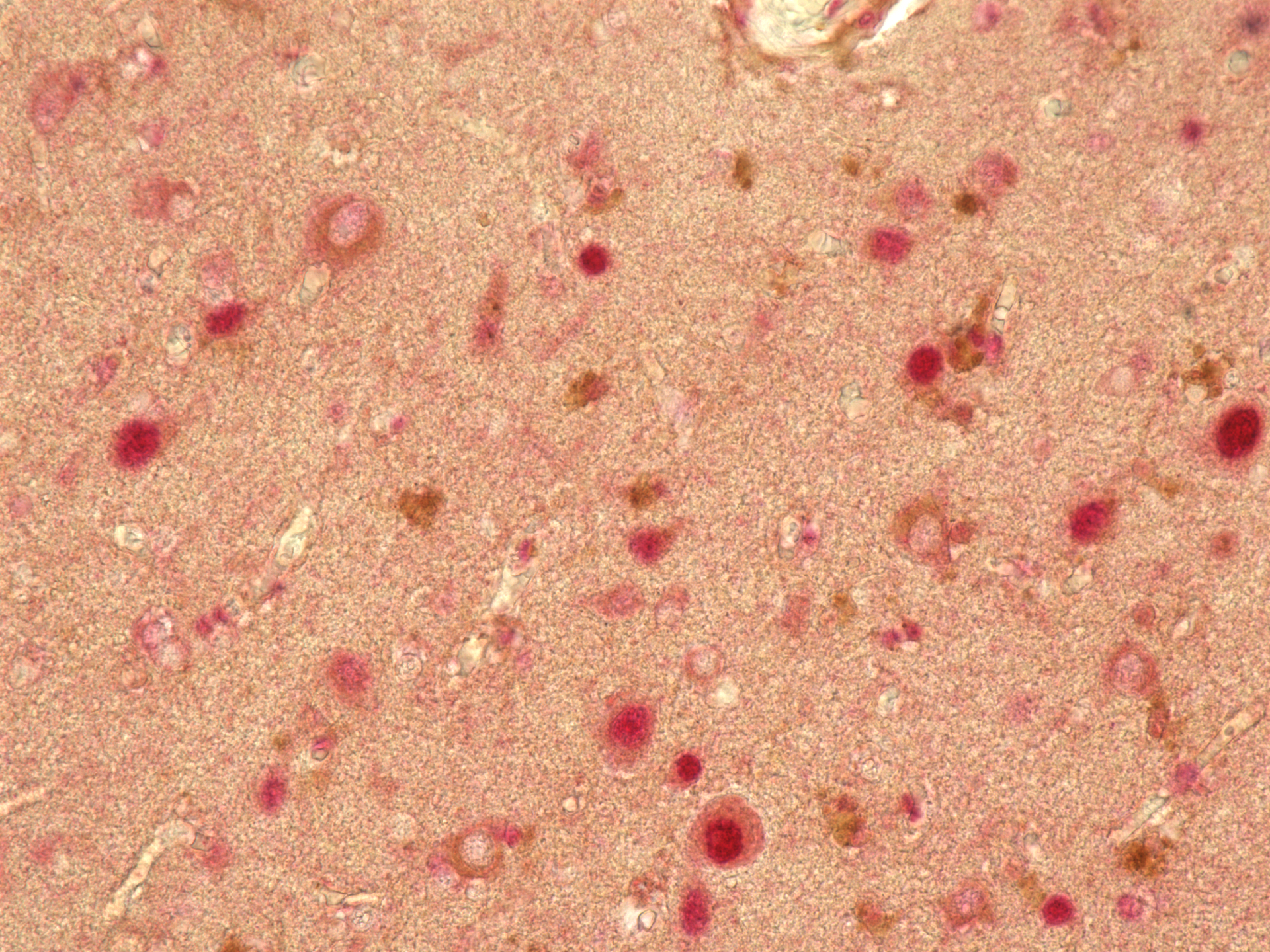

Supplement: Supplementary file 9 — Source data Fig. 5 [file 44318_2024_156_MOESM9_ESM.zip › Figure 5/5B/ALS_GDE2.TDP43_02.tif]

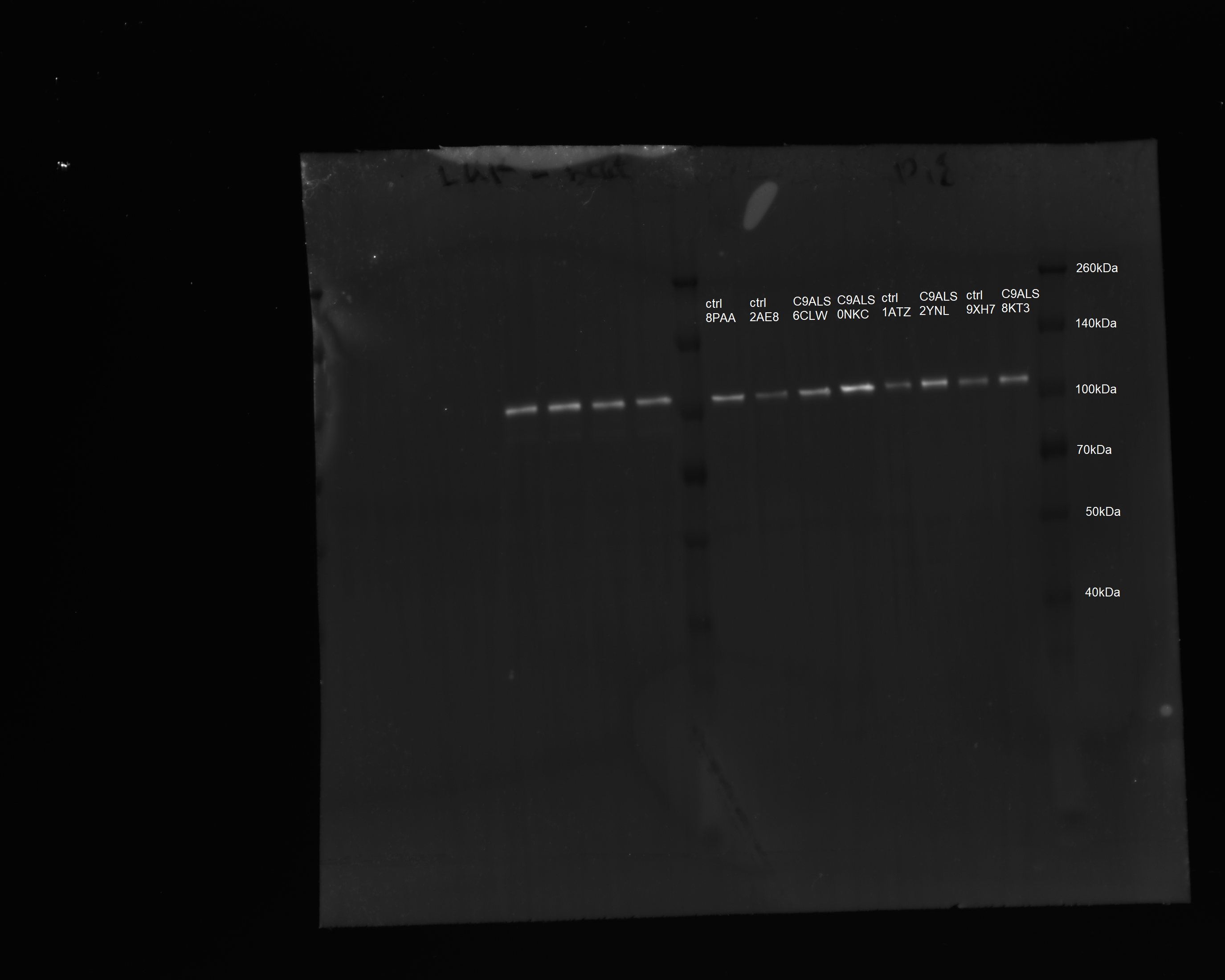

Supplement: Supplementary file 9 — Source data Fig. 5 [file 44318_2024_156_MOESM9_ESM.zip › Figure 5/5F/western non-p-bcat uncropped.tif]

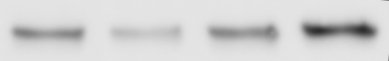

Supplement: Supplementary file 9 — Source data Fig. 5 [file 44318_2024_156_MOESM9_ESM.zip › Figure 5/5F/western non-p-bcat.jpg]

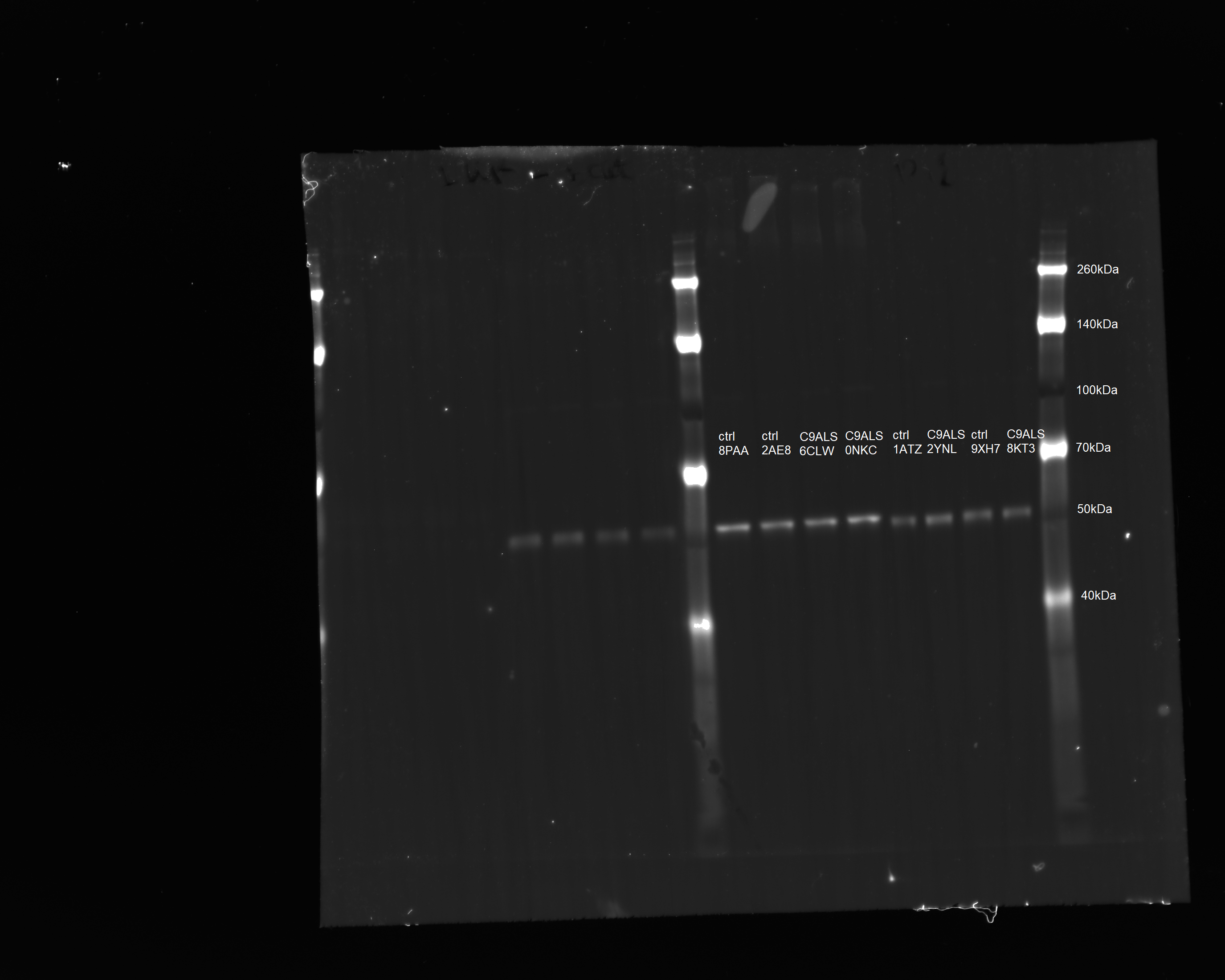

Supplement: Supplementary file 9 — Source data Fig. 5 [file 44318_2024_156_MOESM9_ESM.zip › Figure 5/5F/western Tubulin uncropped.tif]

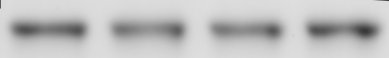

Supplement: Supplementary file 9 — Source data Fig. 5 [file 44318_2024_156_MOESM9_ESM.zip › Figure 5/5F/western Tubulin.jpg]

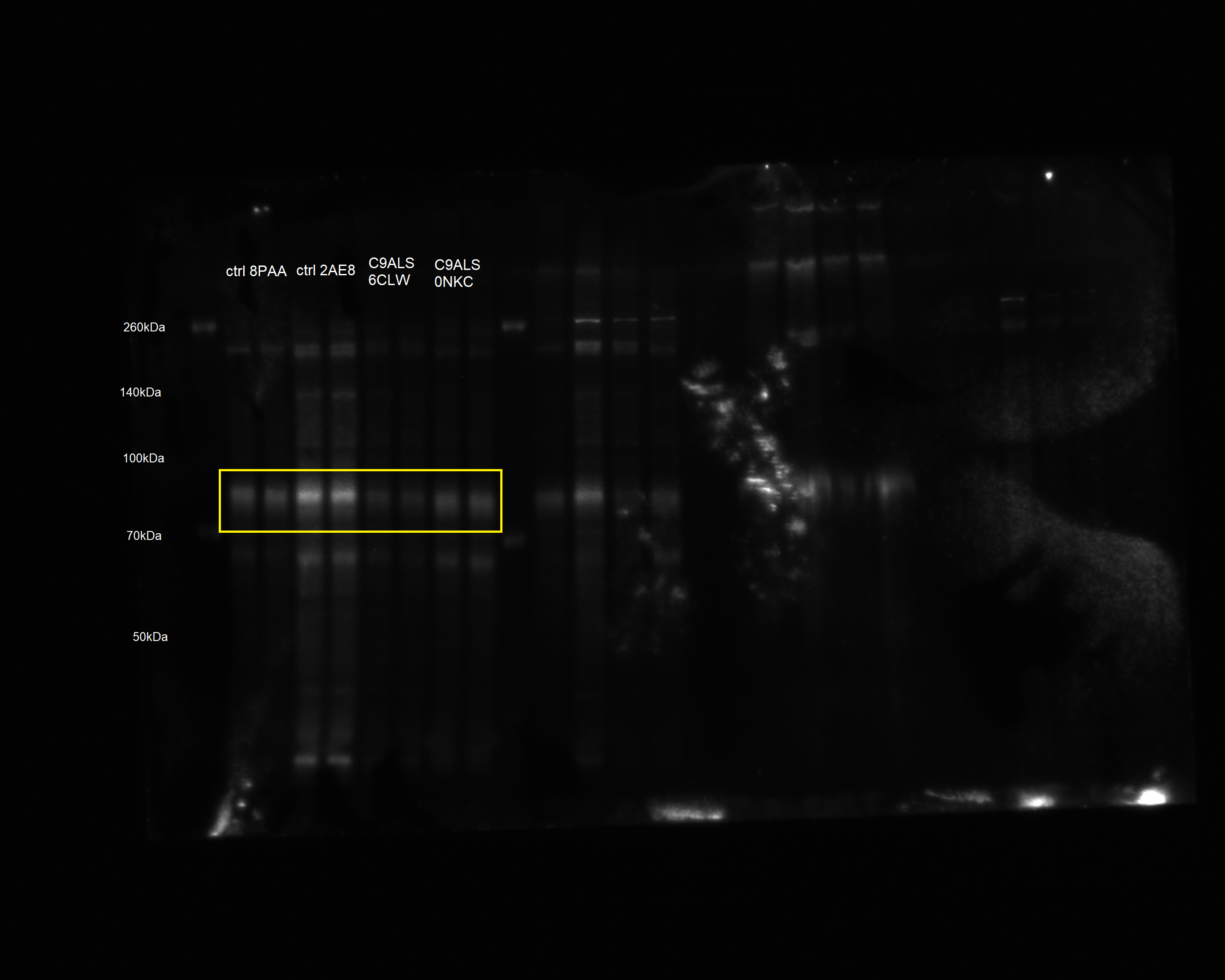

Supplement: Supplementary file 9 — Source data Fig. 5 [file 44318_2024_156_MOESM9_ESM.zip › Figure 5/5H/western GDE2 uncropped.tif]

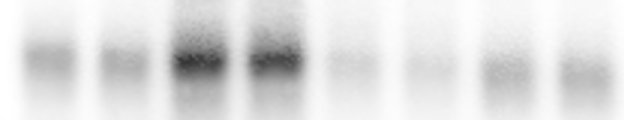

Supplement: Supplementary file 9 — Source data Fig. 5 [file 44318_2024_156_MOESM9_ESM.zip › Figure 5/5H/western GDE2.jpg]

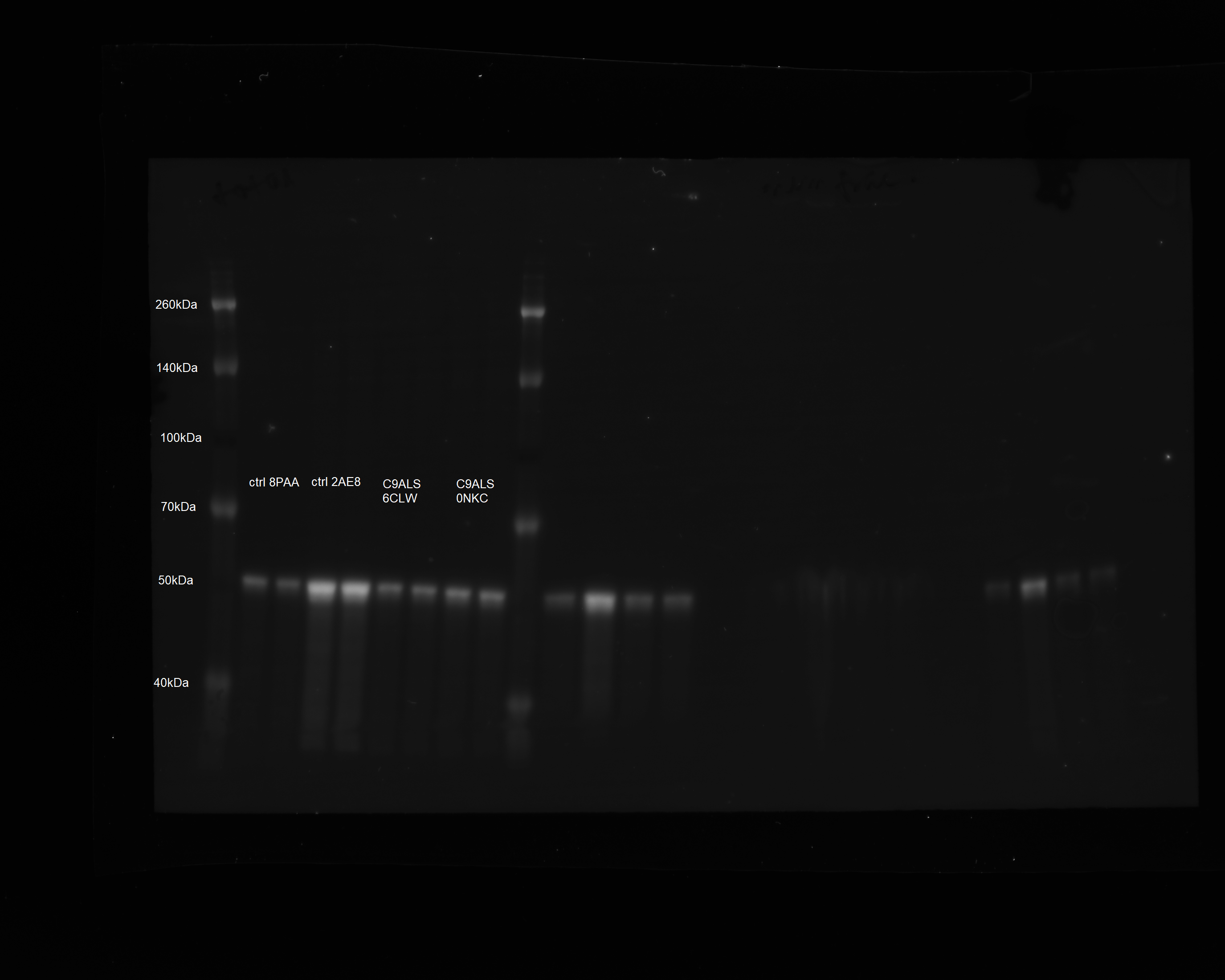

Supplement: Supplementary file 9 — Source data Fig. 5 [file 44318_2024_156_MOESM9_ESM.zip › Figure 5/5H/western Tubulin uncropped.tif]

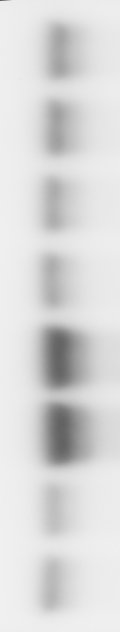

Supplement: Supplementary file 9 — Source data Fig. 5 [file 44318_2024_156_MOESM9_ESM.zip › Figure 5/5H/western Tubulin.jpg]

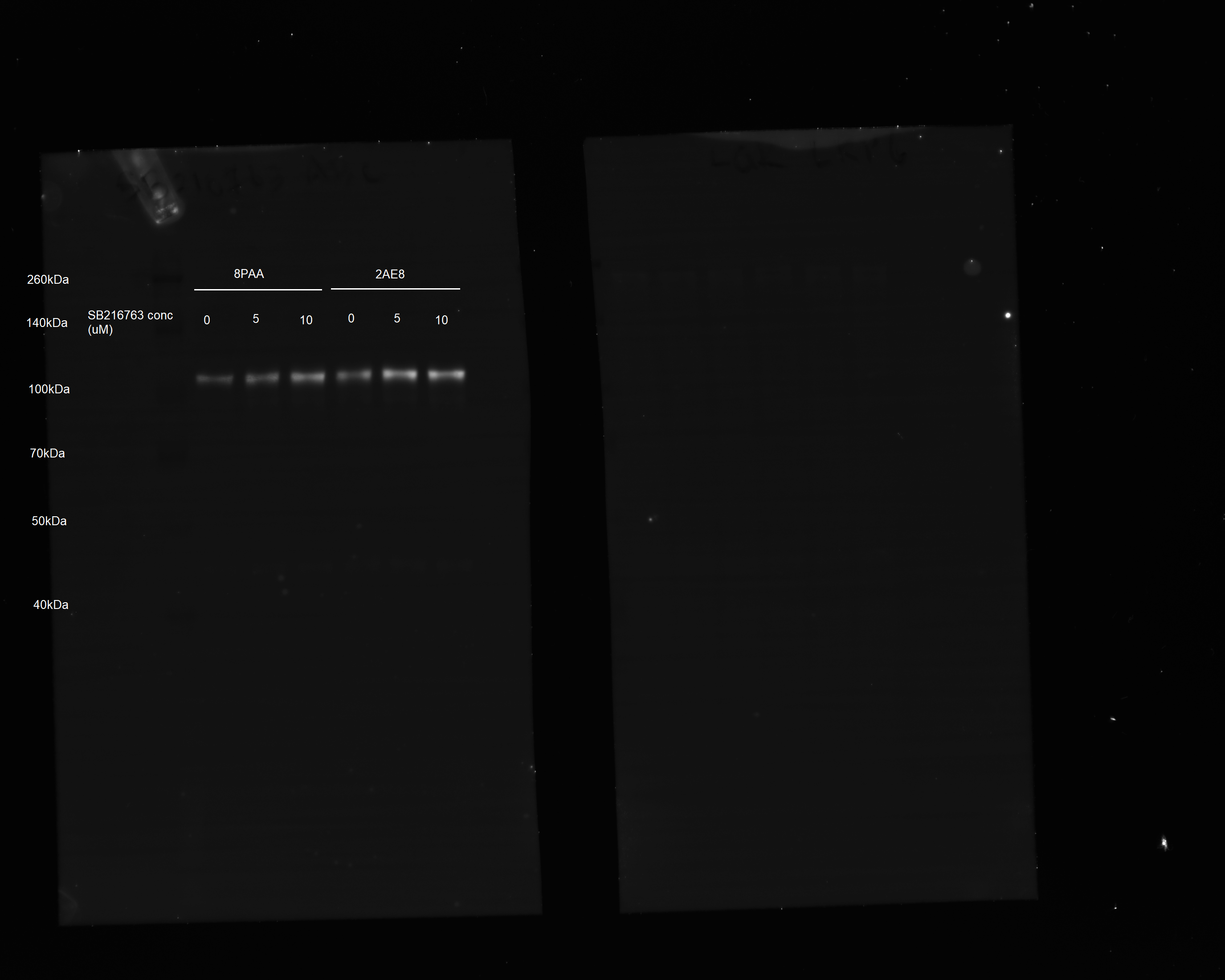

Supplement: Supplementary file 10 — Source data Fig. 6 [file 44318_2024_156_MOESM10_ESM.zip › Figure 6/6A/western non-p-bcat uncropped.tif]

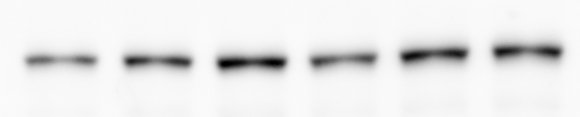

Supplement: Supplementary file 10 — Source data Fig. 6 [file 44318_2024_156_MOESM10_ESM.zip › Figure 6/6A/western non-p-bcat.jpg]

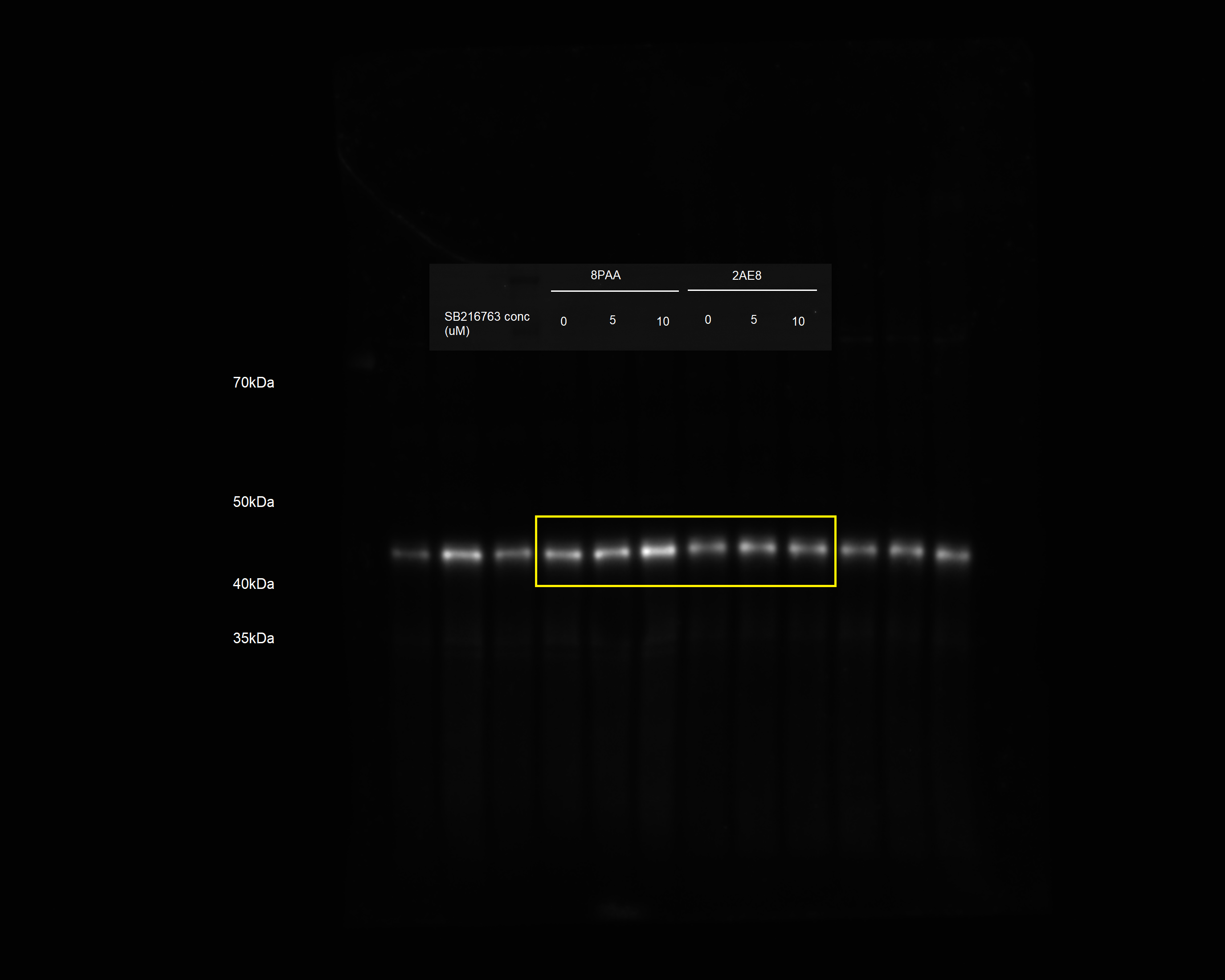

Supplement: Supplementary file 10 — Source data Fig. 6 [file 44318_2024_156_MOESM10_ESM.zip › Figure 6/6A/western TDP-43 uncropped.tif]

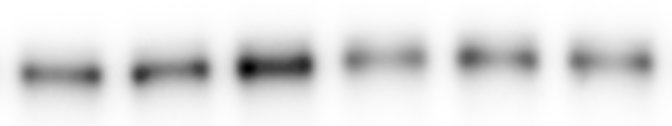

Supplement: Supplementary file 10 — Source data Fig. 6 [file 44318_2024_156_MOESM10_ESM.zip › Figure 6/6A/western TDP43.jpg]

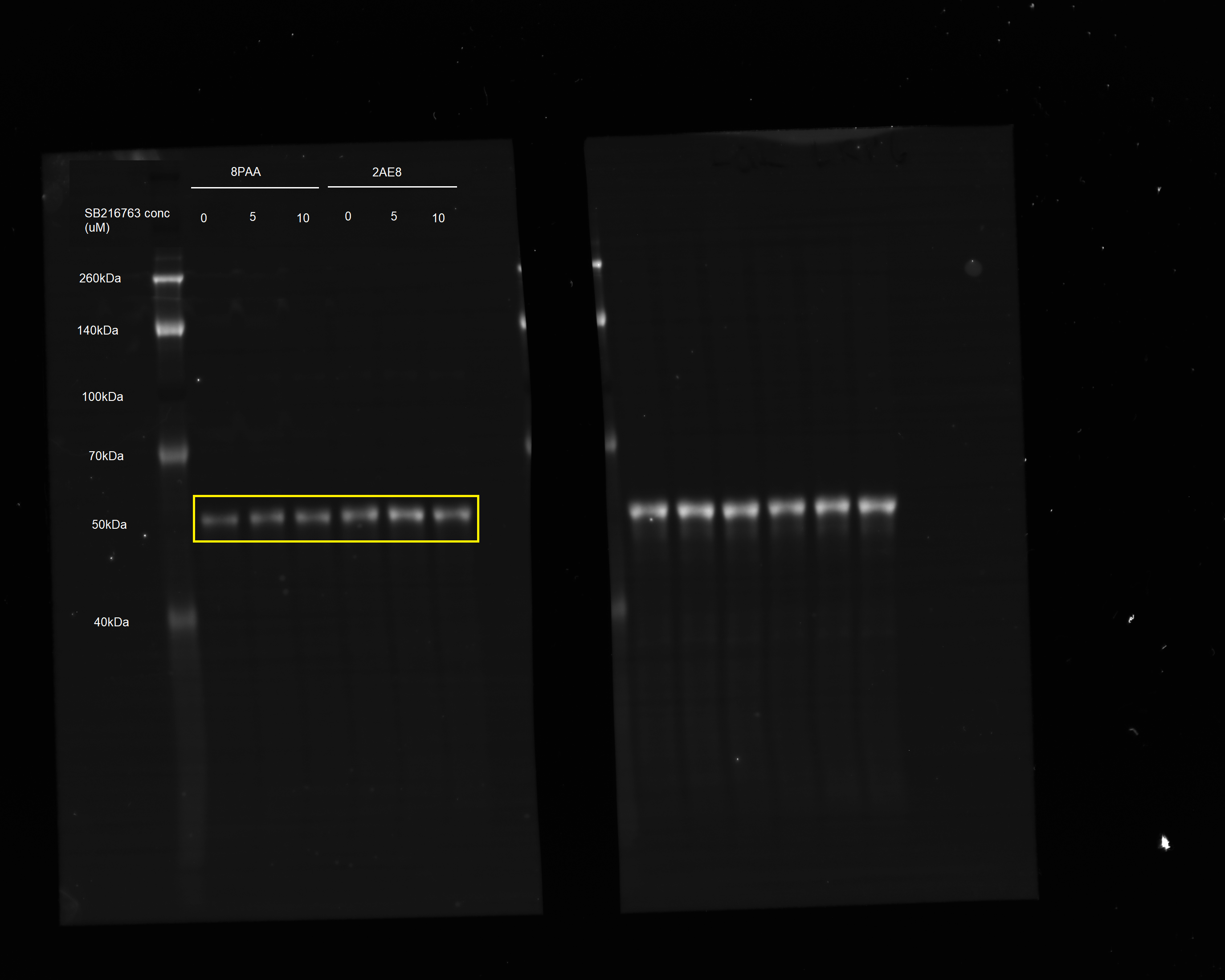

Supplement: Supplementary file 10 — Source data Fig. 6 [file 44318_2024_156_MOESM10_ESM.zip › Figure 6/6A/western Tubulin for bcat uncropped.tif]

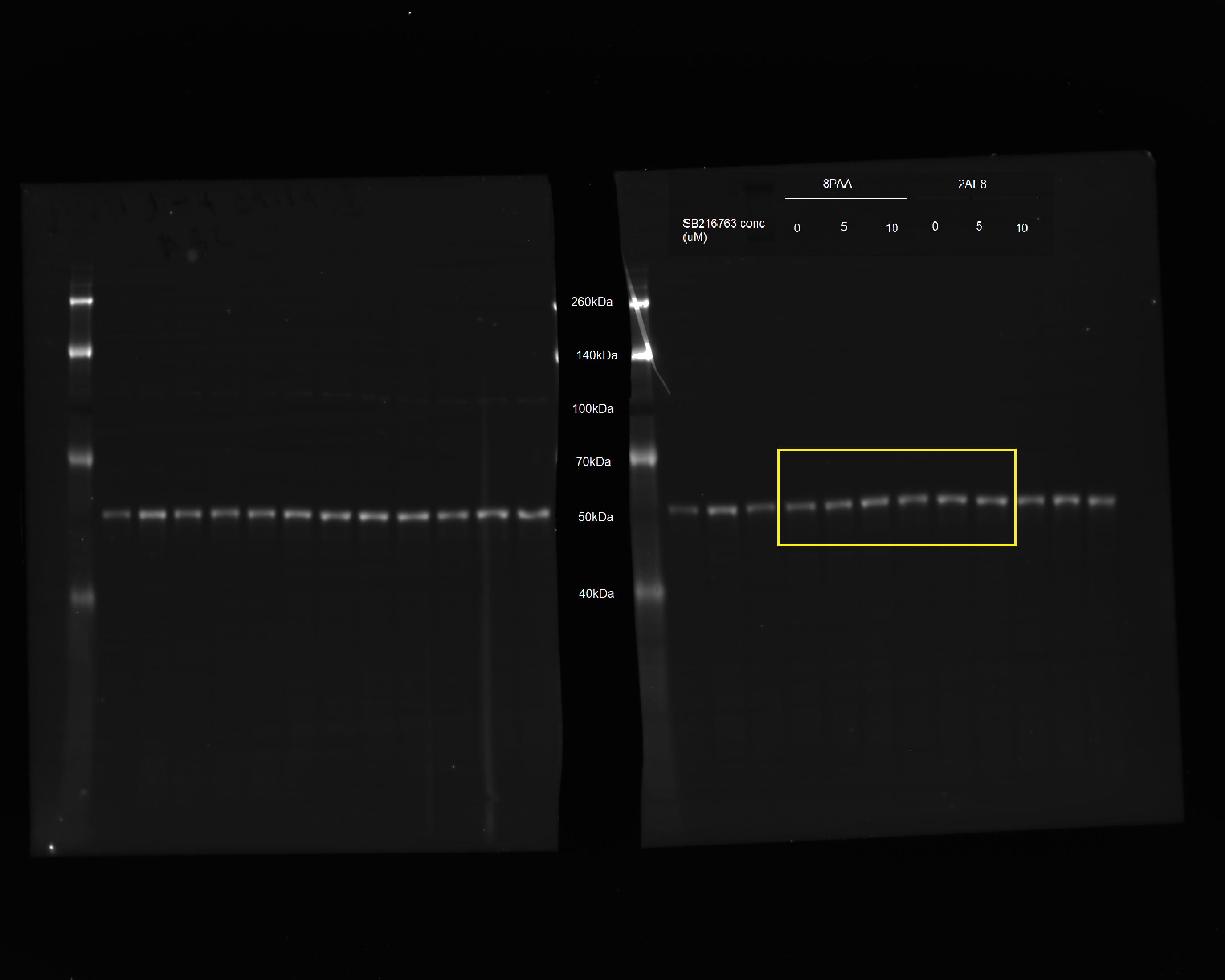

Supplement: Supplementary file 10 — Source data Fig. 6 [file 44318_2024_156_MOESM10_ESM.zip › Figure 6/6A/western Tubulin for TDP-43 uncropped.tif]

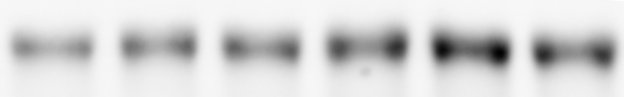

Supplement: Supplementary file 10 — Source data Fig. 6 [file 44318_2024_156_MOESM10_ESM.zip › Figure 6/6A/western Tubulin.jpg]

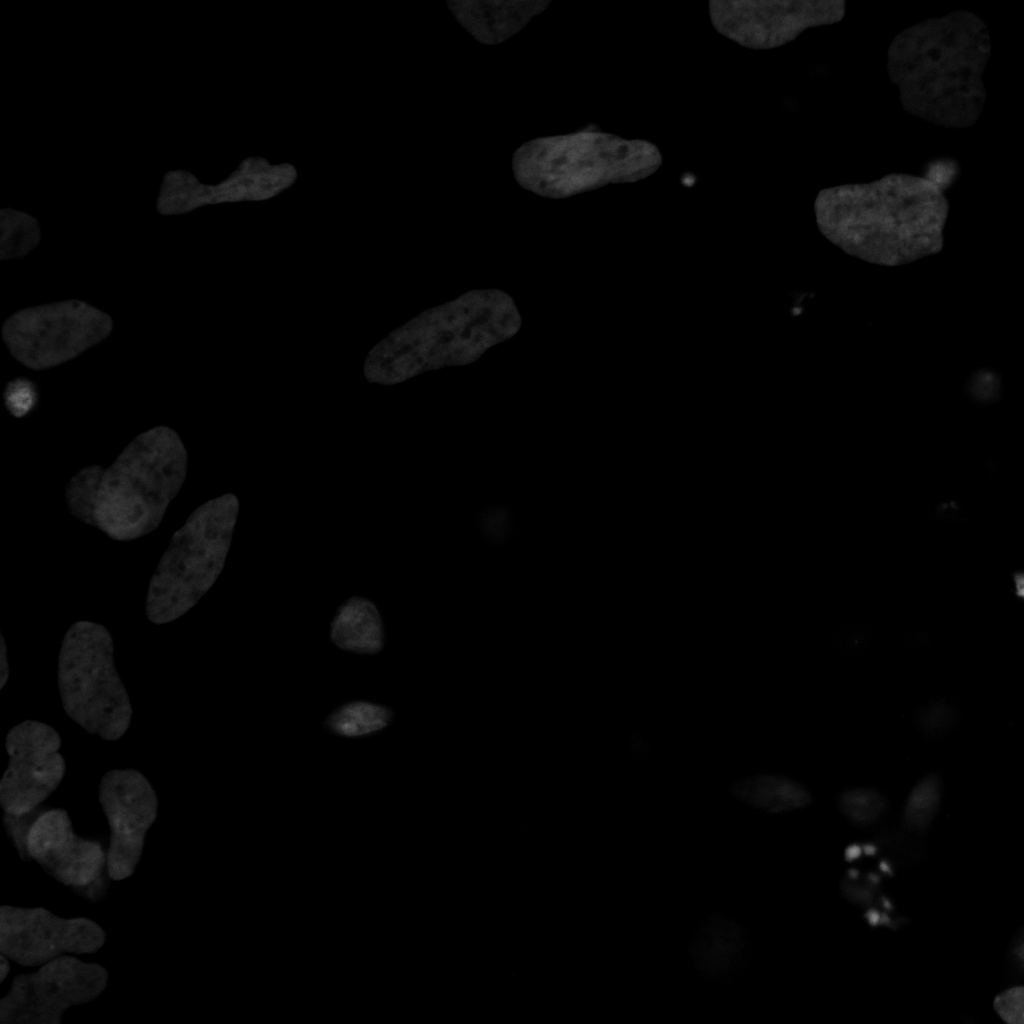

Supplement: Supplementary file 10 — Source data Fig. 6 [file 44318_2024_156_MOESM10_ESM.zip › Figure 6/6E/10uM.tif]

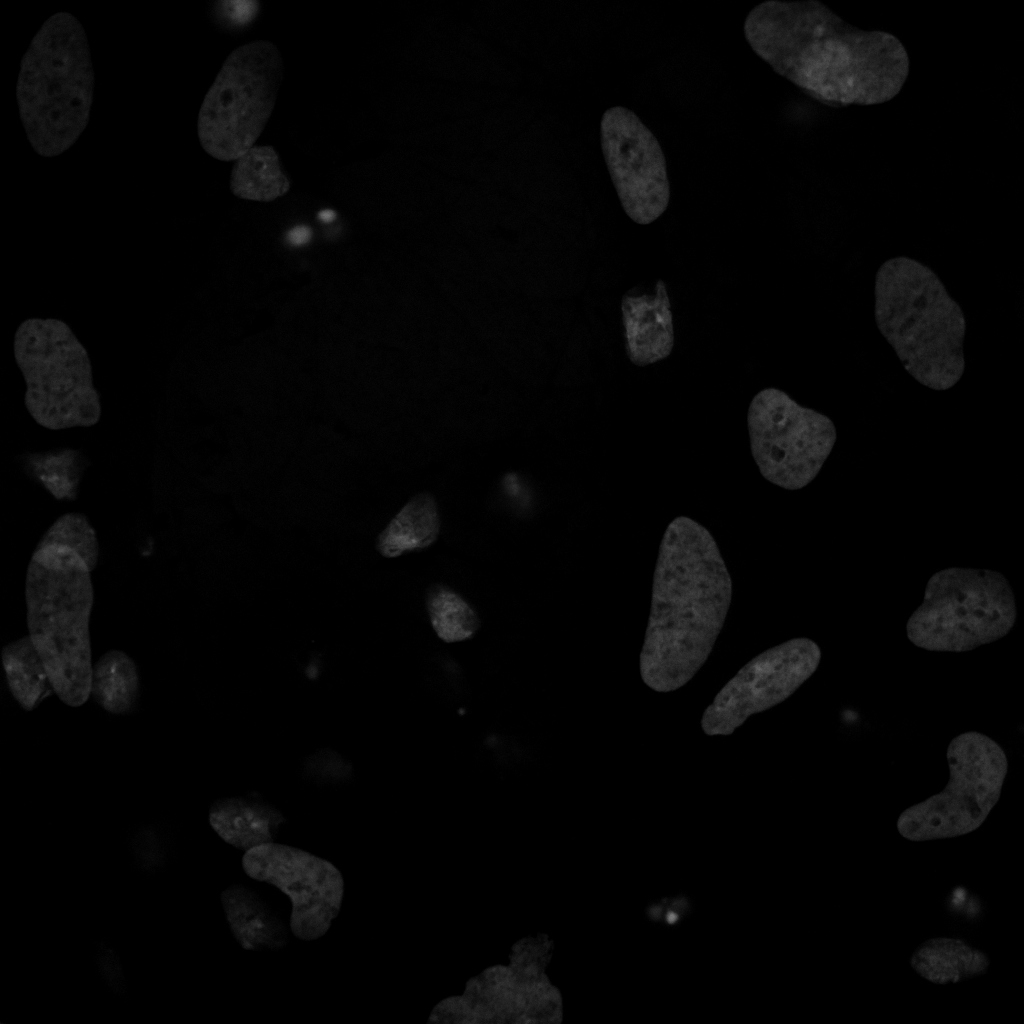

Supplement: Supplementary file 10 — Source data Fig. 6 [file 44318_2024_156_MOESM10_ESM.zip › Figure 6/6E/5uM.tif]

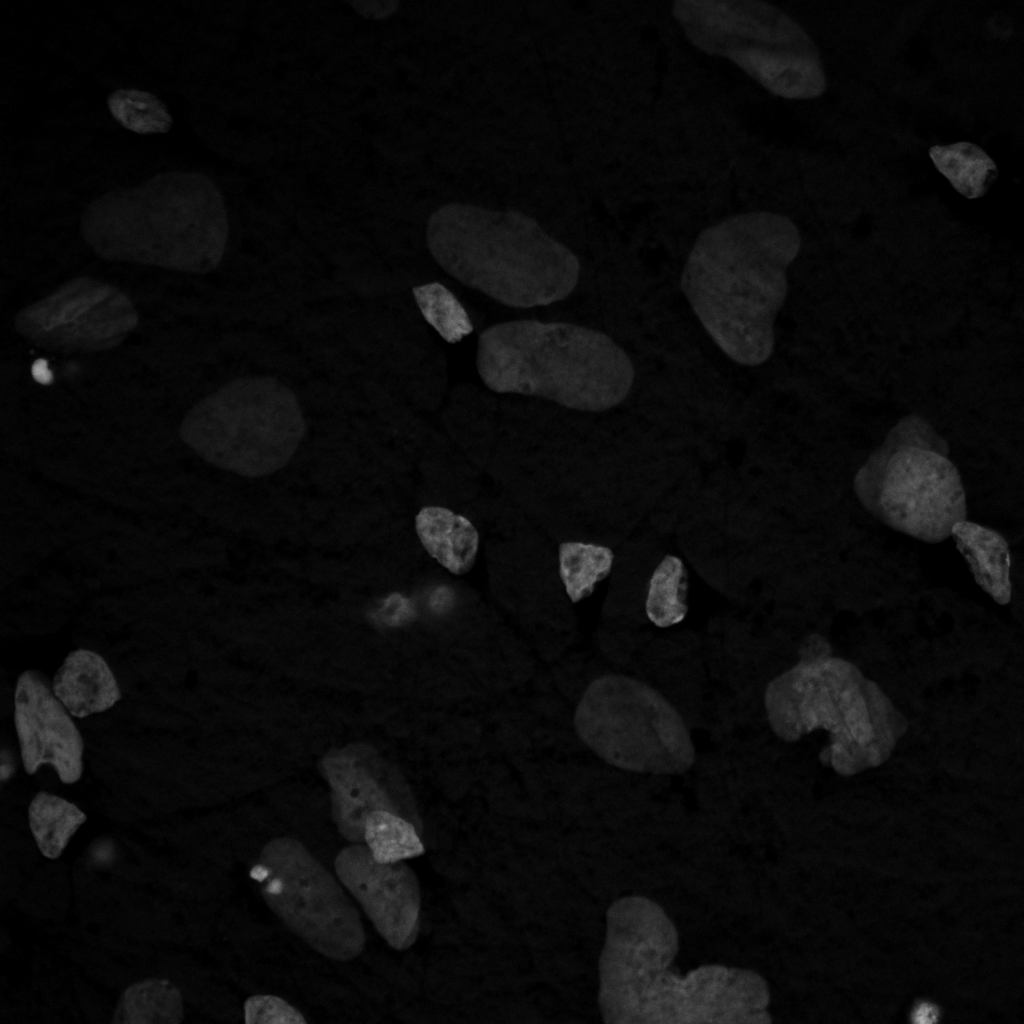

Supplement: Supplementary file 10 — Source data Fig. 6 [file 44318_2024_156_MOESM10_ESM.zip › Figure 6/6E/DMSO.tif]

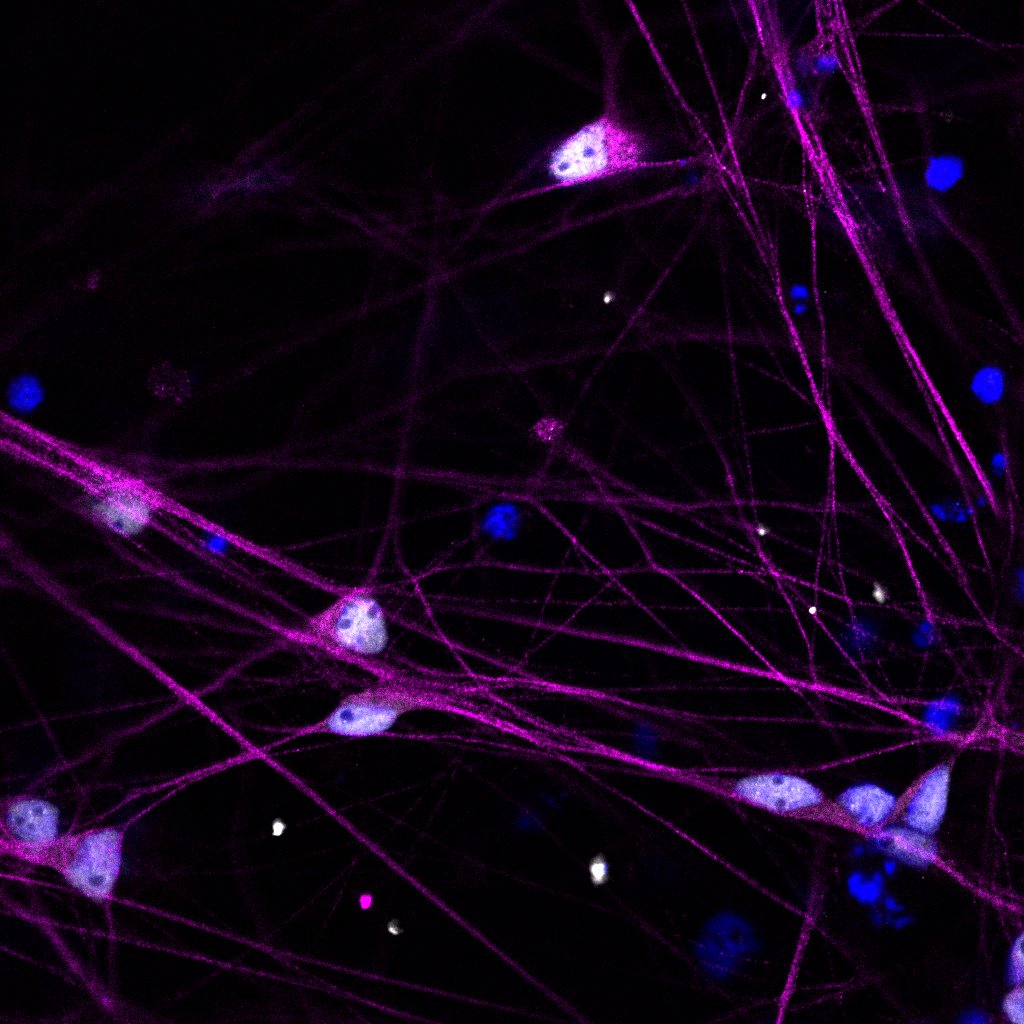

Supplement: Supplementary file 10 — Source data Fig. 6 [file 44318_2024_156_MOESM10_ESM.zip › Figure 6/6E/merge-10uM.jpg]

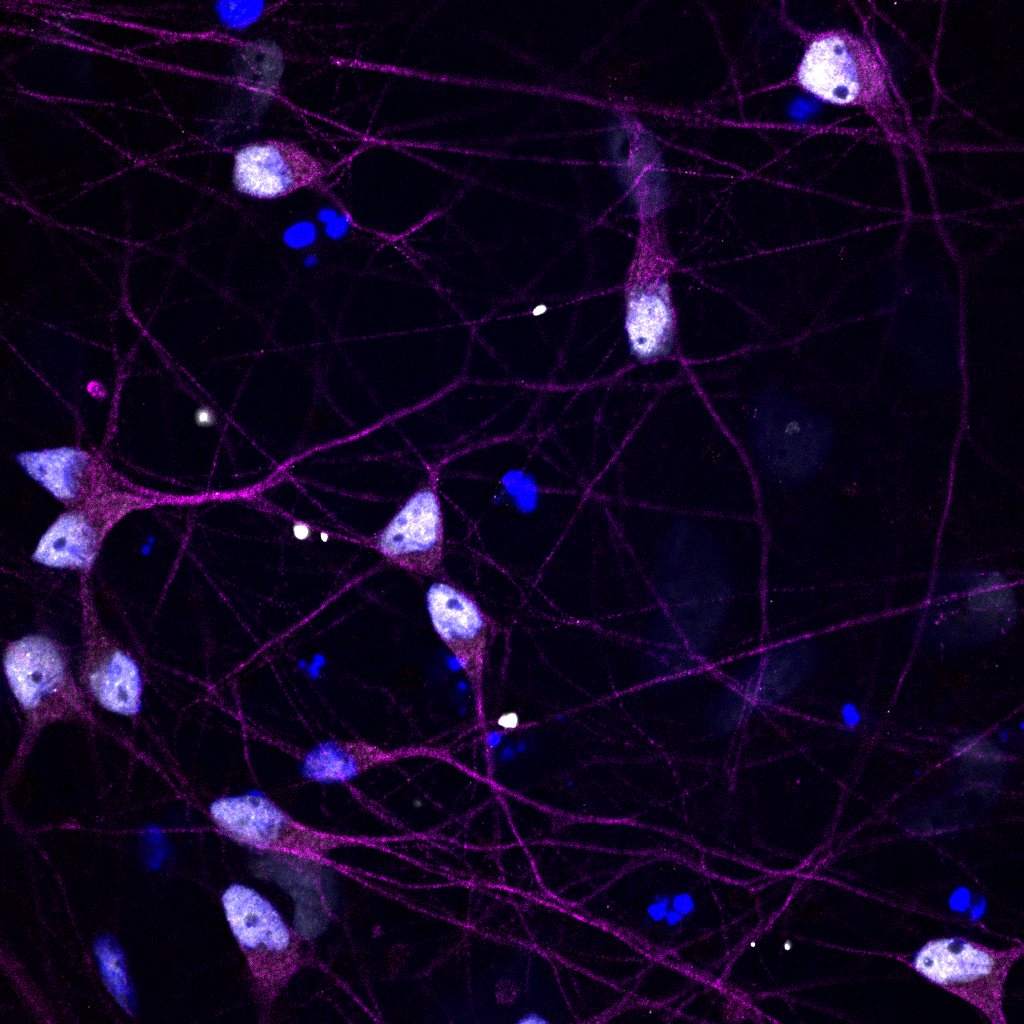

Supplement: Supplementary file 10 — Source data Fig. 6 [file 44318_2024_156_MOESM10_ESM.zip › Figure 6/6E/merge-5uM.jpg]

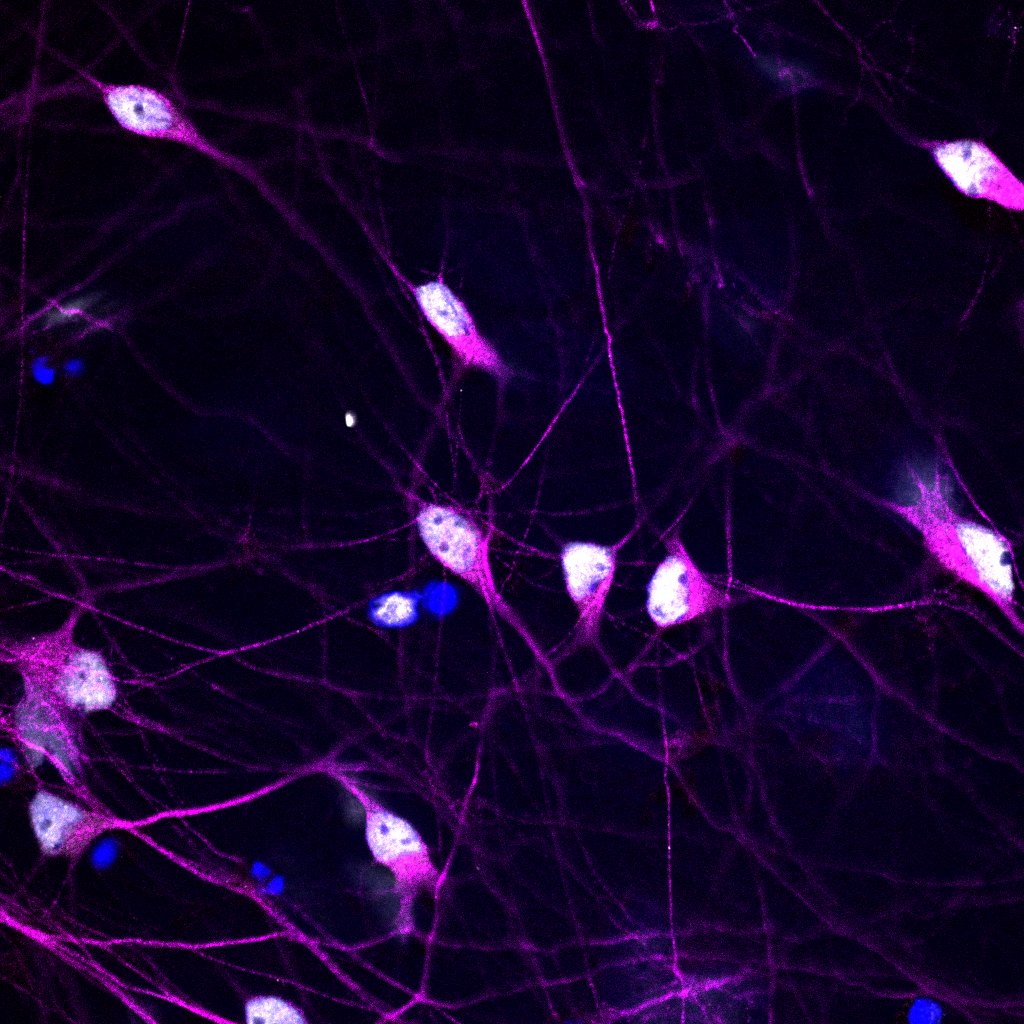

Supplement: Supplementary file 10 — Source data Fig. 6 [file 44318_2024_156_MOESM10_ESM.zip › Figure 6/6E/merge-DMSO.jpg]

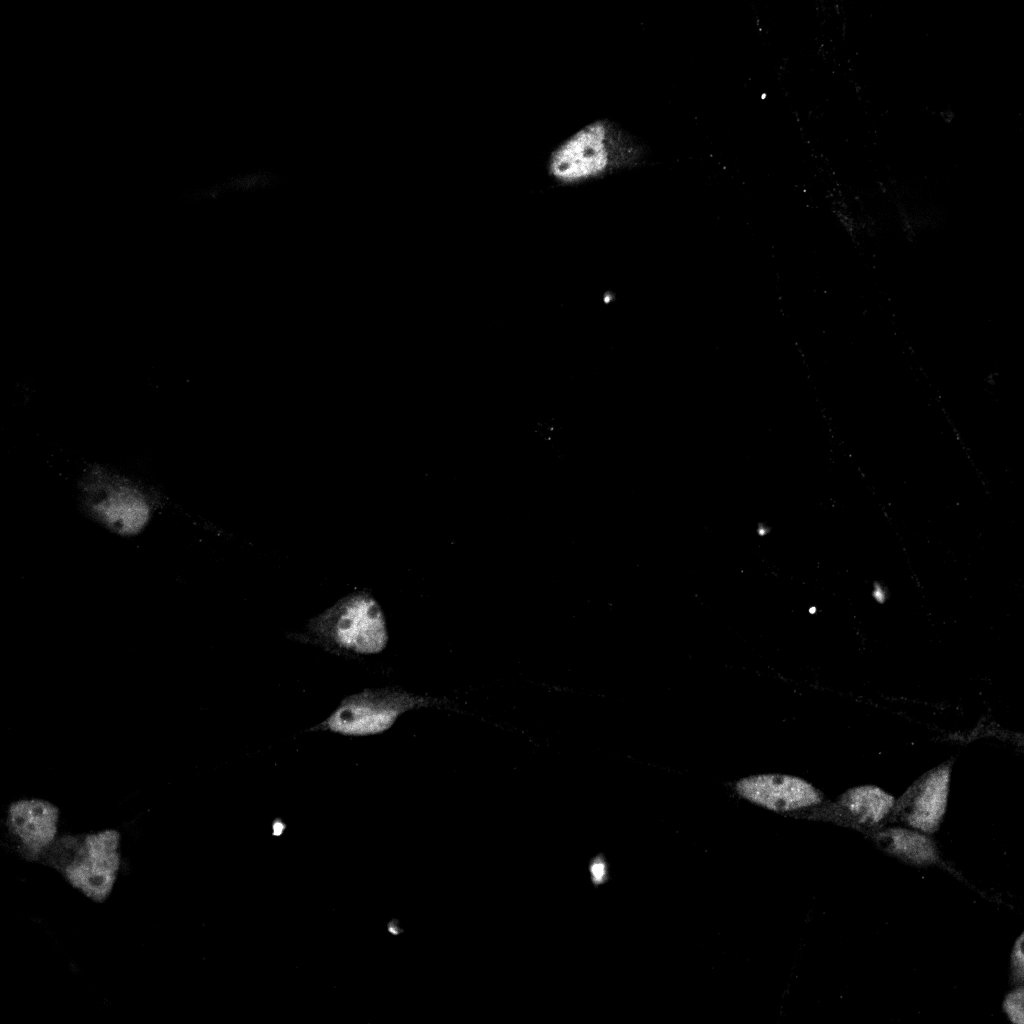

Supplement: Supplementary file 10 — Source data Fig. 6 [file 44318_2024_156_MOESM10_ESM.zip › Figure 6/6E/TDP43-10uM.jpg]

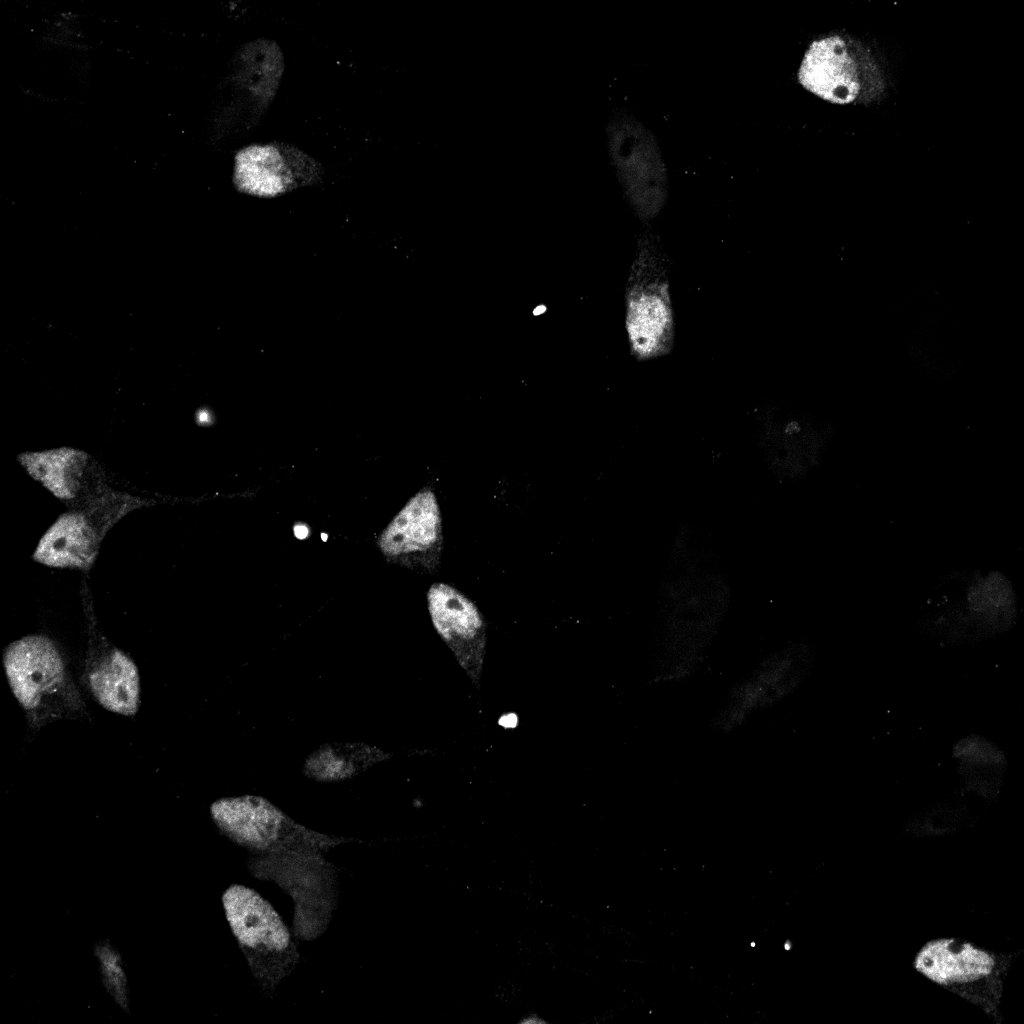

Supplement: Supplementary file 10 — Source data Fig. 6 [file 44318_2024_156_MOESM10_ESM.zip › Figure 6/6E/TDP43-5uM.jpg]

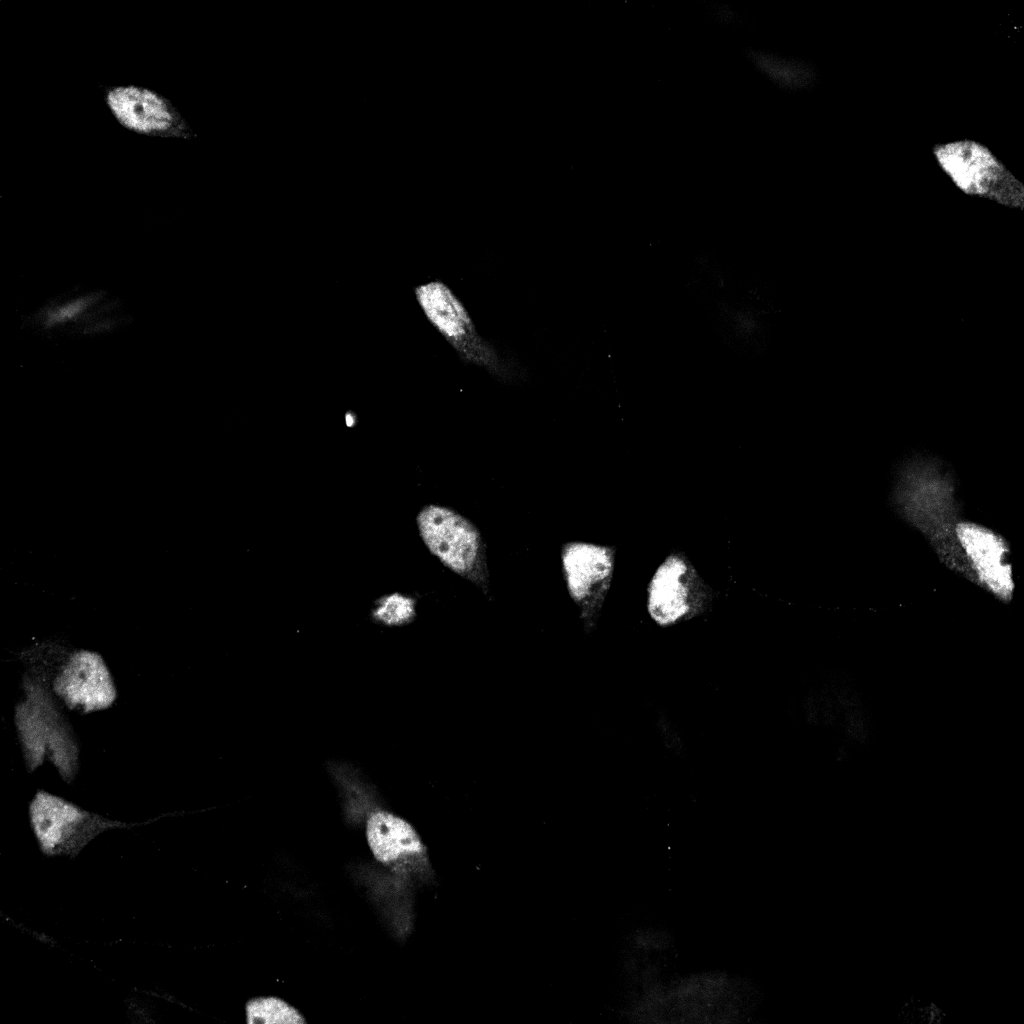

Supplement: Supplementary file 10 — Source data Fig. 6 [file 44318_2024_156_MOESM10_ESM.zip › Figure 6/6E/TDP43-DMSO.jpg]

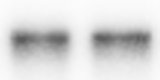

Supplement: Supplementary file 10 — Source data Fig. 6 [file 44318_2024_156_MOESM10_ESM.zip › Figure 6/6G/western LRP6.jpg]

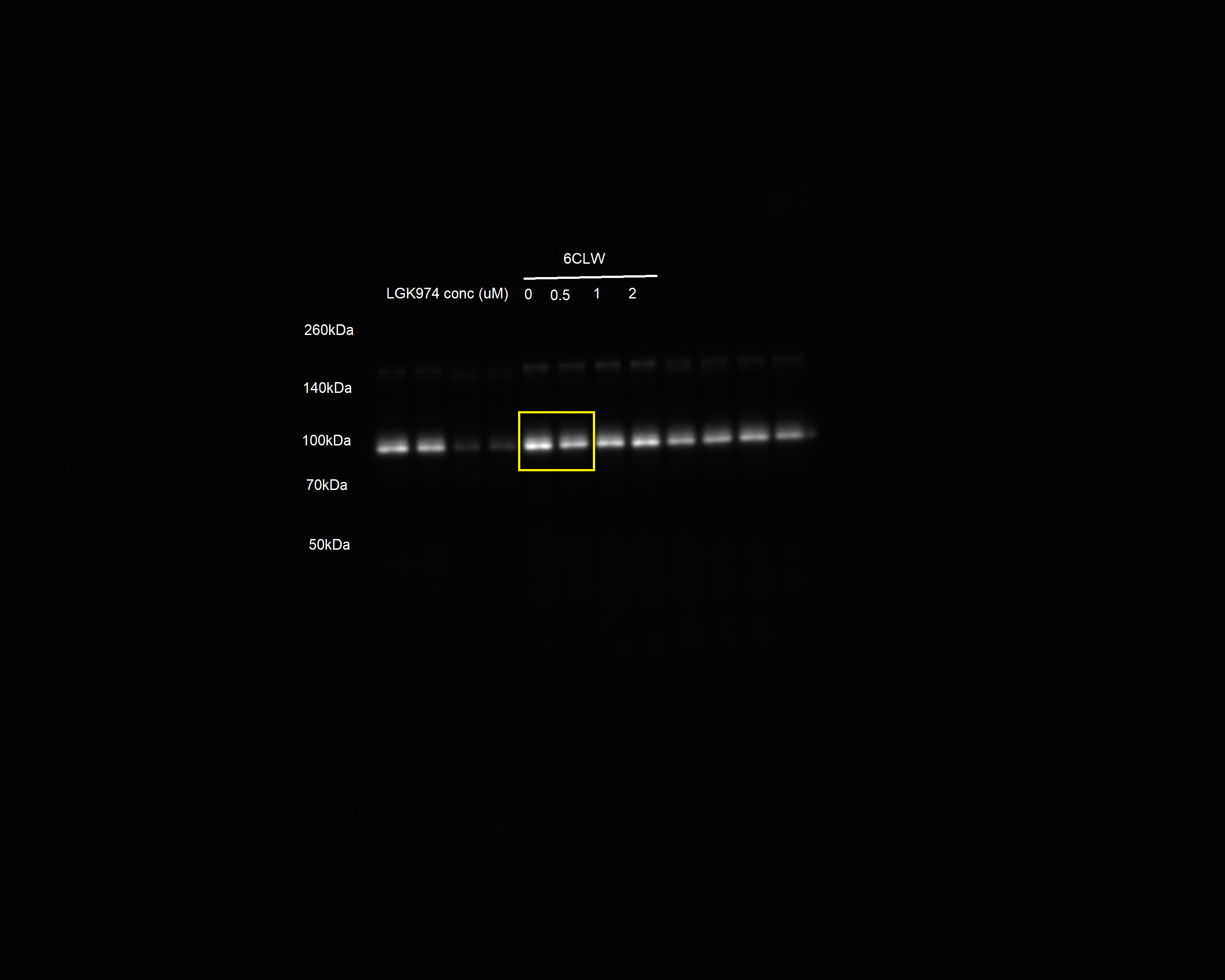

Supplement: Supplementary file 10 — Source data Fig. 6 [file 44318_2024_156_MOESM10_ESM.zip › Figure 6/6G/western non-p-bcat uncropped.tif]

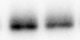

Supplement: Supplementary file 10 — Source data Fig. 6 [file 44318_2024_156_MOESM10_ESM.zip › Figure 6/6G/western non-p-bcat.jpg]

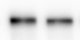

Supplement: Supplementary file 10 — Source data Fig. 6 [file 44318_2024_156_MOESM10_ESM.zip › Figure 6/6G/western pLRP6.jpg]

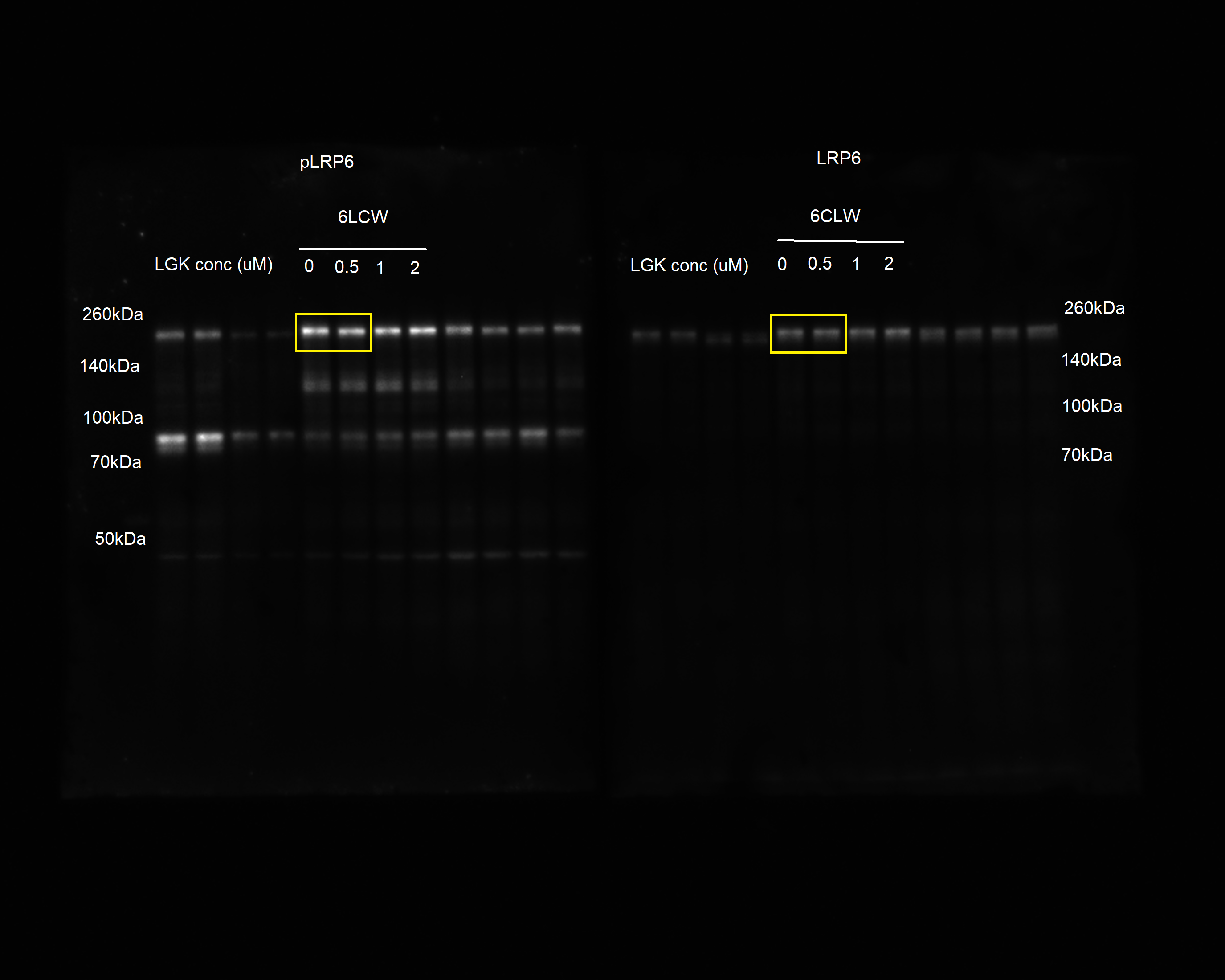

Supplement: Supplementary file 10 — Source data Fig. 6 [file 44318_2024_156_MOESM10_ESM.zip › Figure 6/6G/western pLRP6-LRP6 uncropped.tif]
