# Supplementary figures and images for: Physiological regulation of neuronal Wnt activity is essential for TDP-43 localization and function (part 2 of 2)
Source: EMBO J. 2024 Jun 25;43(16):6. doi: 10.1038/s44318-024-00156-8 (PMC11329687; doi:10.1038/s44318-024-00156-8)

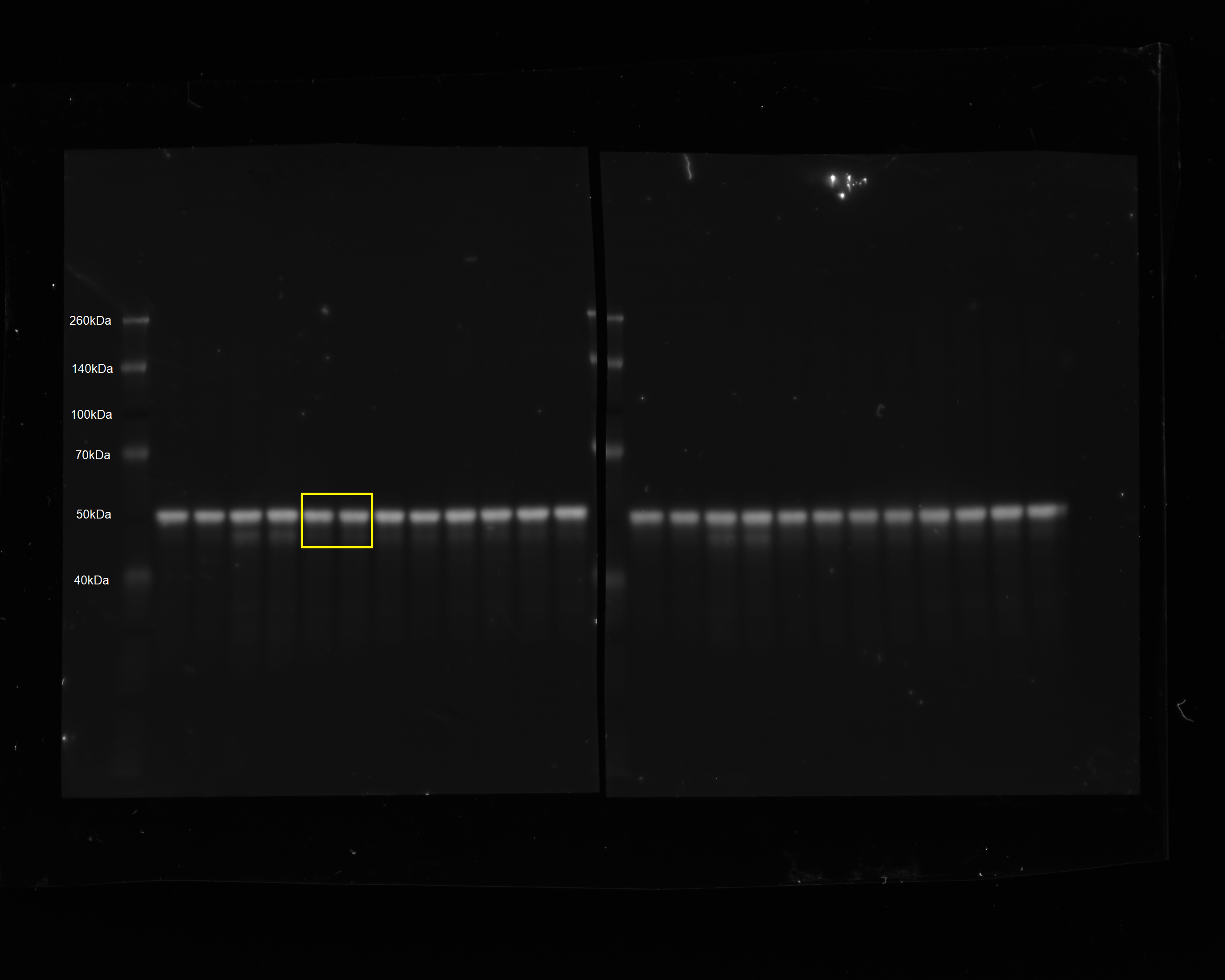

Supplement: Supplementary file 10 — Source data Fig. 6 [file 44318_2024_156_MOESM10_ESM.zip › Figure 6/6G/western Tubulin uncropped.tif]

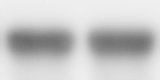

Supplement: Supplementary file 10 — Source data Fig. 6 [file 44318_2024_156_MOESM10_ESM.zip › Figure 6/6G/western Tubulin.jpg]
