# Supplementary material for: Physiological regulation of neuronal Wnt activity is essential for TDP-43 localization and function
Source: EMBO J. 2024 Jun 25;43(16):6. doi: 10.1038/s44318-024-00156-8 (PMC11329687; doi:10.1038/s44318-024-00156-8)
Supplement: Supplementary file 11 — Expanded View Figures [file 44318_2024_156_MOESM11_ESM.pdf]

Expanded View Figures

Figure EV1. GDE2 loss leads to neurodegeneration and neuronal loss.

(**A–H**) Representative images of immunohistochemical staining of cortical sections of 19-month animals. (**d'–d'''**) highlight changes in microglial morphology in *Gde2*KOs. Scale bar: (**A–H**) = 50µm; (**c'–d'''**) = 15 µm. (**I–L**) Graphs quantifying area fraction for astrogliosis (**I**, GFAP, $*P = 0.0147$), microglial activation (**J**, Iba1, $*P = 0.0127$), neuronal numbers (**K**, NeuN, $*P = 0.0495$), and deep-layer neurons (**L**, Ctip2, $*P = 0.0180$). All graphs: mean ± s.e.m., Unpaired *t* test. $n = 5$-11 WT, 5-11 *Gde2*KO.

© The Author(s)

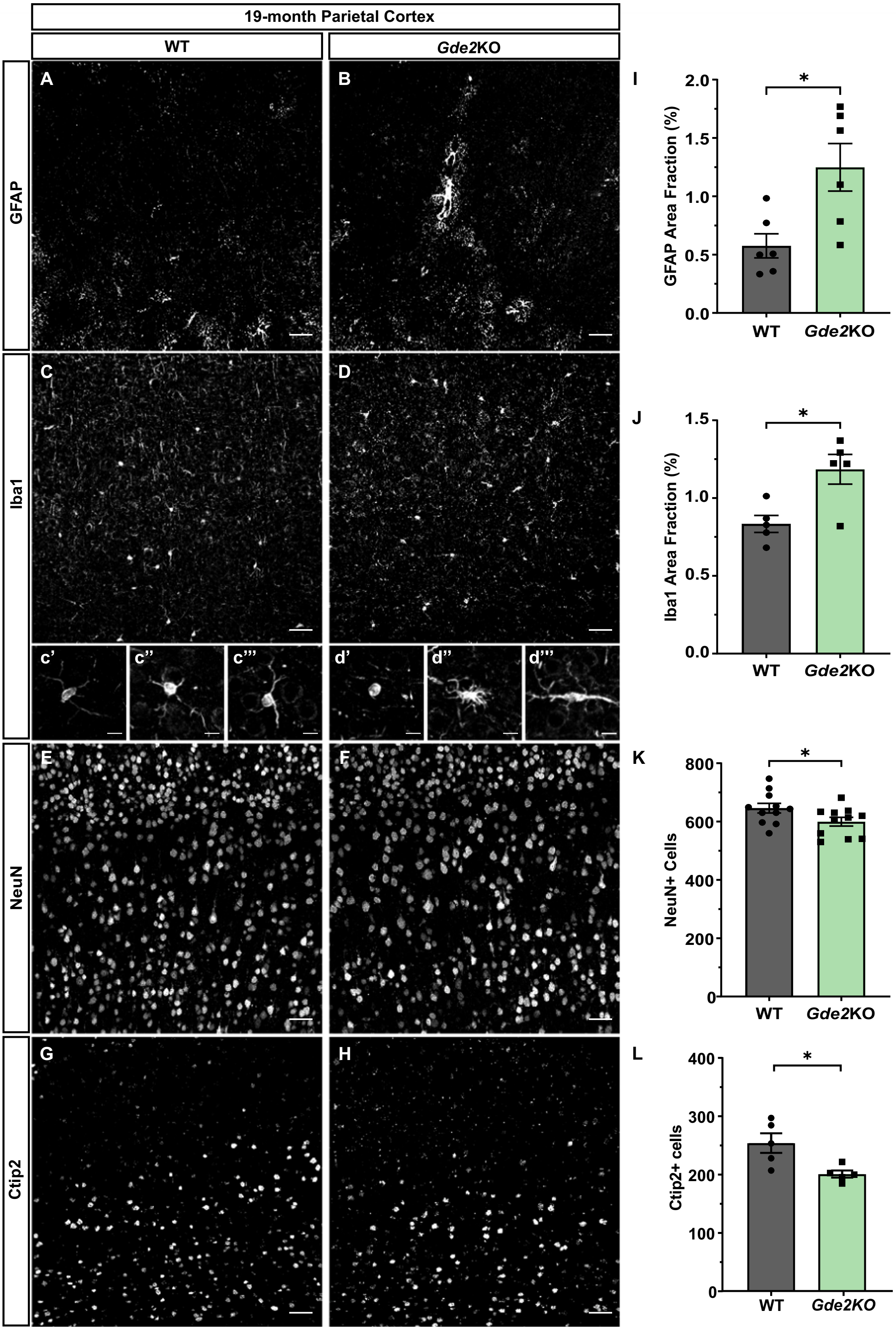
19-month Parietal Cortex
WT
Gde2KO
GFAP
Iba1
NeuN
Ctip2
A
B
C
D
c'
c''
c'''
d'
d''
d'''
E
F
G
H
I
GFAP Area Fraction (%)
2.0
1.5
1.0
0.5
0.0
*
WT
Gde2KO
J
Iba1 Area Fraction (%)
1.5
1.0
0.5
0.0
*
WT
Gde2KO
K
NeuN+ Cells
800
600
400
200
0
*
WT
Gde2KO
L
Ctip2+ cells
400
300
200
100
0
*
WT
Gde2KO

 © The Author(s)

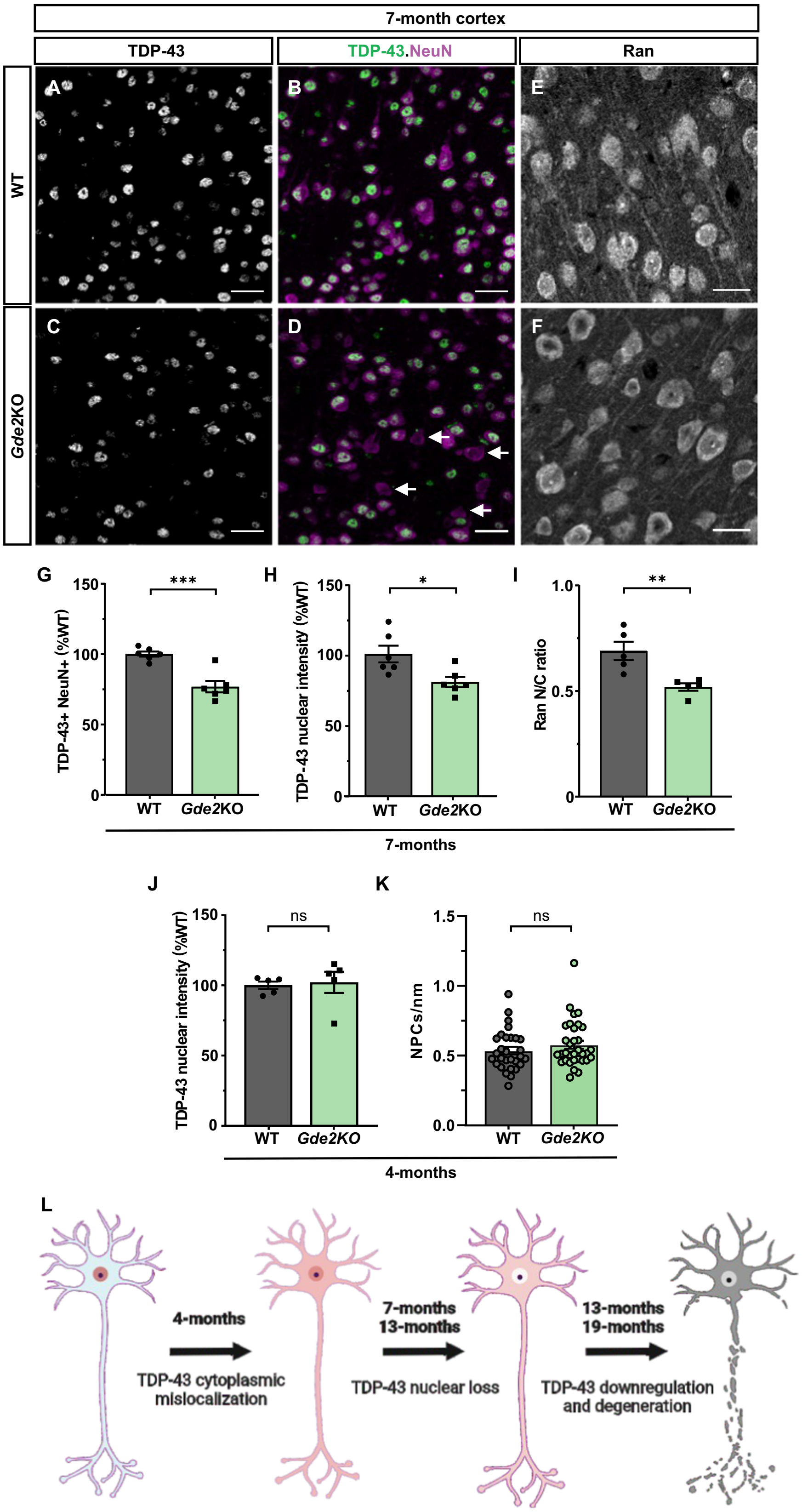
7-month cortex
TDP-43
TDP-43.NeuN
Ran
WT
Gde2KO
A
B
C
D
E
F
G
H
I
TDP-43+ NeuN+ (%WT)
TDP-43 nuclear intensitY (%WT)
Ran N/C ratio
***
*
**
WT
Gde2KO
7-months
J
K
TDP-43 nuclear intensity (%WT)
NPCs/nm
ns
4-months
L
4-months
TDP-43 cytoplasmic mislocalization
7-months
13-months
TDP-43 nuclear loss
13-months
19-months
TDP-43 downregulation and degeneration

Figure EV2. Progression of TDP-43 expression and NPC number in WT and *Gde2*KO animals.

(**A–F**) Representative images of immunohistochemical staining of cortical sections of 7-month WT and *Gde2*KO animals. Arrows (**D**) highlight neurons lacking TDP-43 expression. Scale bar: (**A–F**) = 25 µm. (**G–I**) Graphs quantifying the percentage of neurons expressing TDP-43 (**G**, ***$P = 0.0004$), neuronal TDP-43 nuclear intensity (**H**, *$P = 0.0173$), and Ran N/C ratio (**I**, **$P = 0.0066$) in 7-month old animals. $n = 6$ WT, 6 *Gde2*KO. (**J**) Graph quantifying neuronal TDP-43 nuclear intensity in 4-month animals (**J**, ns $P = 0.7977$). $n = 5$ WT, 5 *Gde2*KO. (**K**) Graph quantifying NPCs per nm from WT and *Gde2*KO TEM micrographs (ns $P = 0.2859$). $n = 29$ WT cells, 33 *Gde2*KO cells, 3 animals/genotype. (**L**) Schematic describing the progression of TDP-43 expression over time in *Gde2*KO mice. All graphs: mean ± s.e.m., Unpaired *t* test. Schematic in (**L**) created with BioRender.com

 © The Author(s)

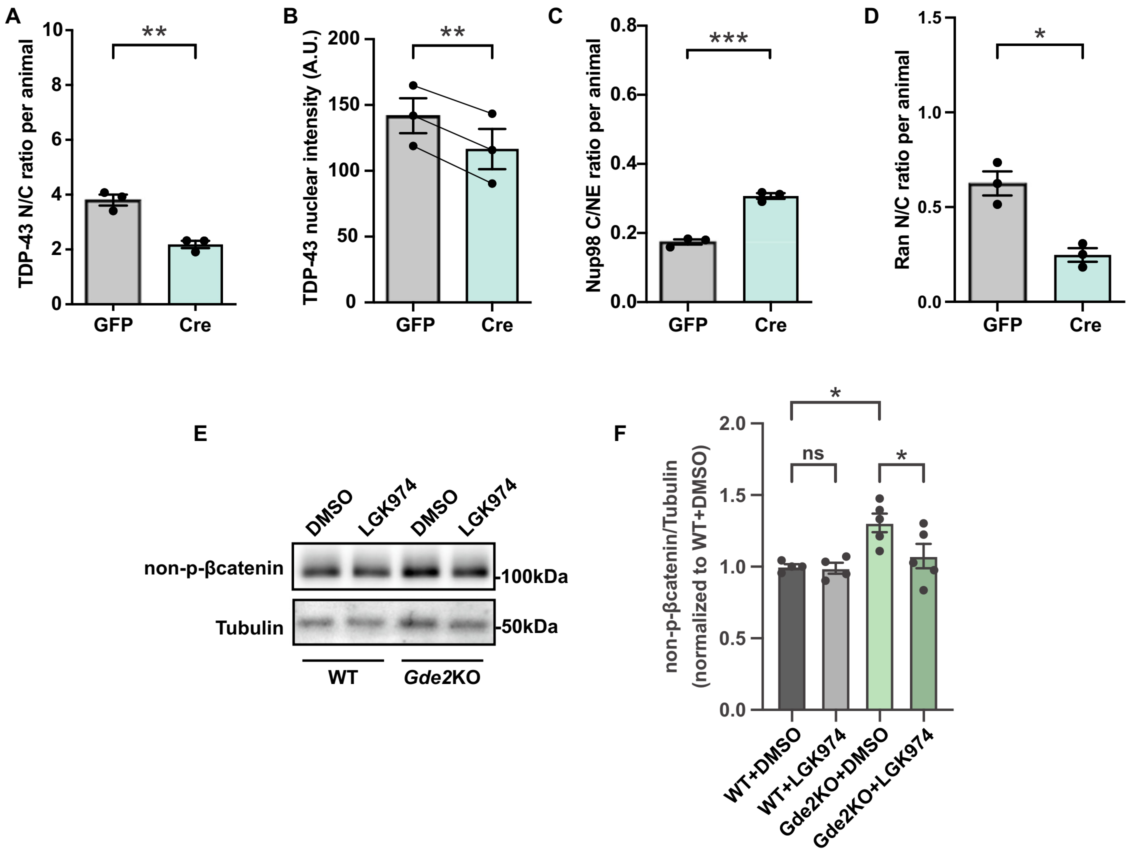

Figure EV3. Stabilized endogenous β-catenin causes TDP-43 nuclear exclusion, changes Nup98 distribution, and disrupts NCT.

(**A–D**) Presentation of data from Fig. 4M–P comparing the number of animals analyzed. *Ctnnb*^{flex3} mice were injected with AAV expressing GFP or Cre.GFP. Graphs quantifying neuronal TDP-43 nuclear/cytoplasmic ratio (**A**, $^{**}P = 0.0041$), TDP-43 nuclear intensity (**B**, $^{**}P = 0.0066$; A.U.= arbitrary units), Nup98 cytoplasmic/nuclear envelope ratio (**C**, $^{***}P = 0.0003$) and Ran nuclear/cytoplasmic ratio (**D**, $^{*}P = 0.0125$). $n = 3$ animals per condition. (**E**) Representative Western blot of primary cortical neurons prepared from WT and *Gde2*KO mice treated with vehicle (DMSO) or LGK974. (**F**) Graph quantifying non-phosphorylated β-catenin in WT and *Gde2*KO cultured cortical neurons treated with DMSO or LGK974 (WT + DMSO/WT + LGK974) ns = 0.9993, (WT + DMSO/*Gde2*KO + DMSO) $^{*}P = 0.0128$, (*Gde2*KO + DMSO/*Gde2*KO + LGK974) $^{*}P = 0.0472$. $n = 4$ WT and 5 *Gde2*KO cultures. All graphs: mean ± s.e.m., (**A**, **C**, **D**): Welch's *t* test; (**B**): paired *t* test. (**F**) One-way ANOVA with Šidák's multiple comparisons test.

© The Author(s)

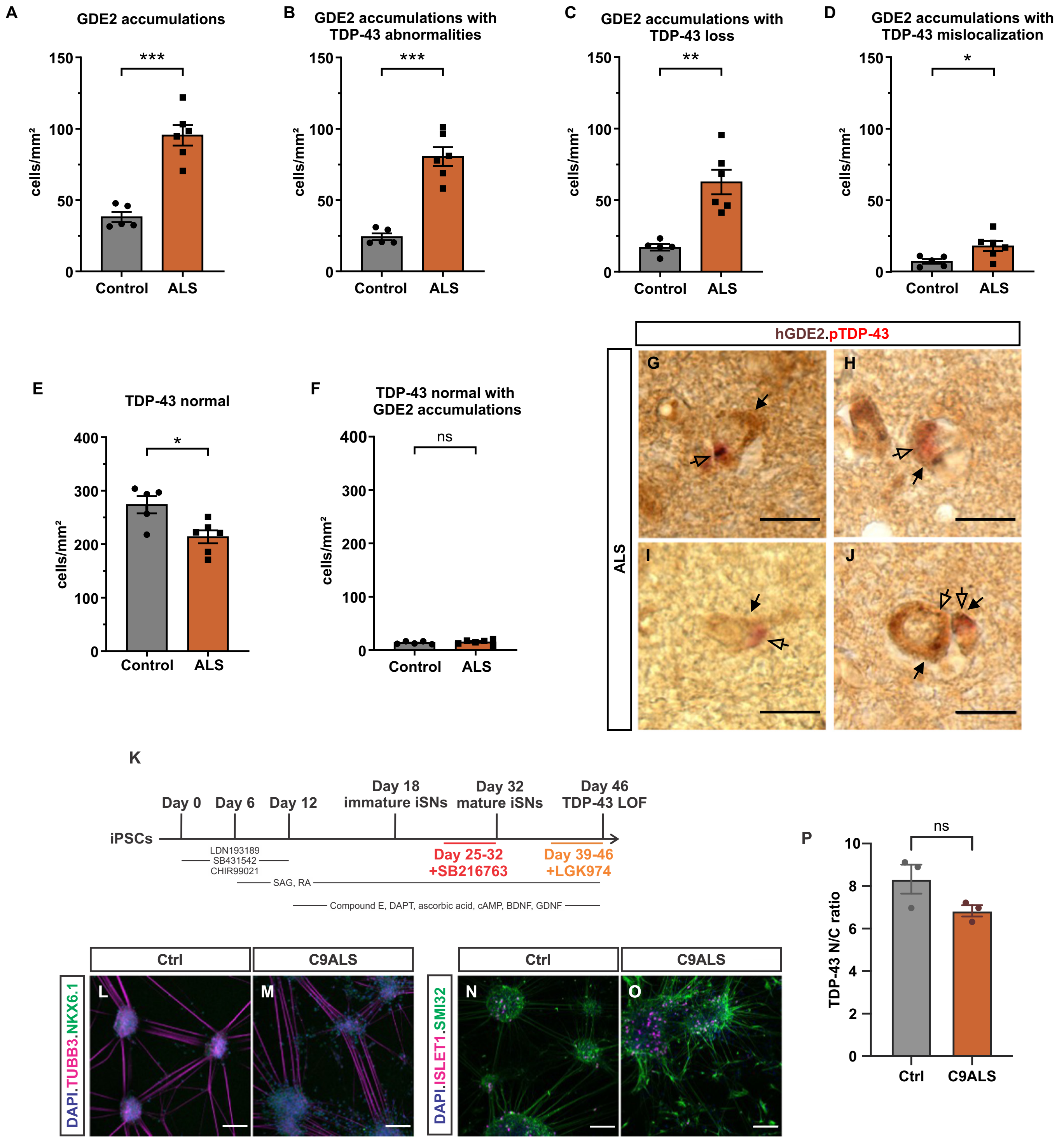

Figure EV4. Analysis of GDE2 and TDP-43 expression in postmortem brain of control and patients with ALS, and differentiation of iSNs.

(**A–F**) Graphs showing comparisons between control individuals and patients with ALS from compiled graphs in Fig. 5A,B. (**A**) ***$P = 0.0002$; (**B**) ***$P = 0.0002$; (**C**) **$P = 0.0025$; (**D**) *$P = 0.0322$; (**E**) *$P = 0.0144$; (**F**) ns $P = 0.6542$. $n = 5$ controls, $n = 6$ patients with ALS. (**G–J**) Representative images of immunohistochemical staining from postmortem ALS patient brains showing exemplar cells with hGDE2 accumulations (arrows) that overlap with pTDP-43 inclusions (open arrows). Scale bar = 15 μm. (**K**) Schematic detailing the differentiation timeline of iSNs from iPSCs. (**L–O**) Representative images of immunocytochemical staining of Day 32 iSNs for the motor neuron and dorsal interneuron marker ISLET1, the progenitor marker Nkx6.1, tubulin (TUBB3), and neurofilament (SMI32). Scale bar = 100 μm. (**P**) Graph comparing TDP-43 N/C ratios in Day 46 Ctrl and C9ALS iSNs. ns $P = 0.1122$, $n = 3$. All graphs: mean ± s.e.m., (**A–C**) Welch's *t* test; (**D–F**, **P**) Unpaired *t* test.

 © The Author(s)

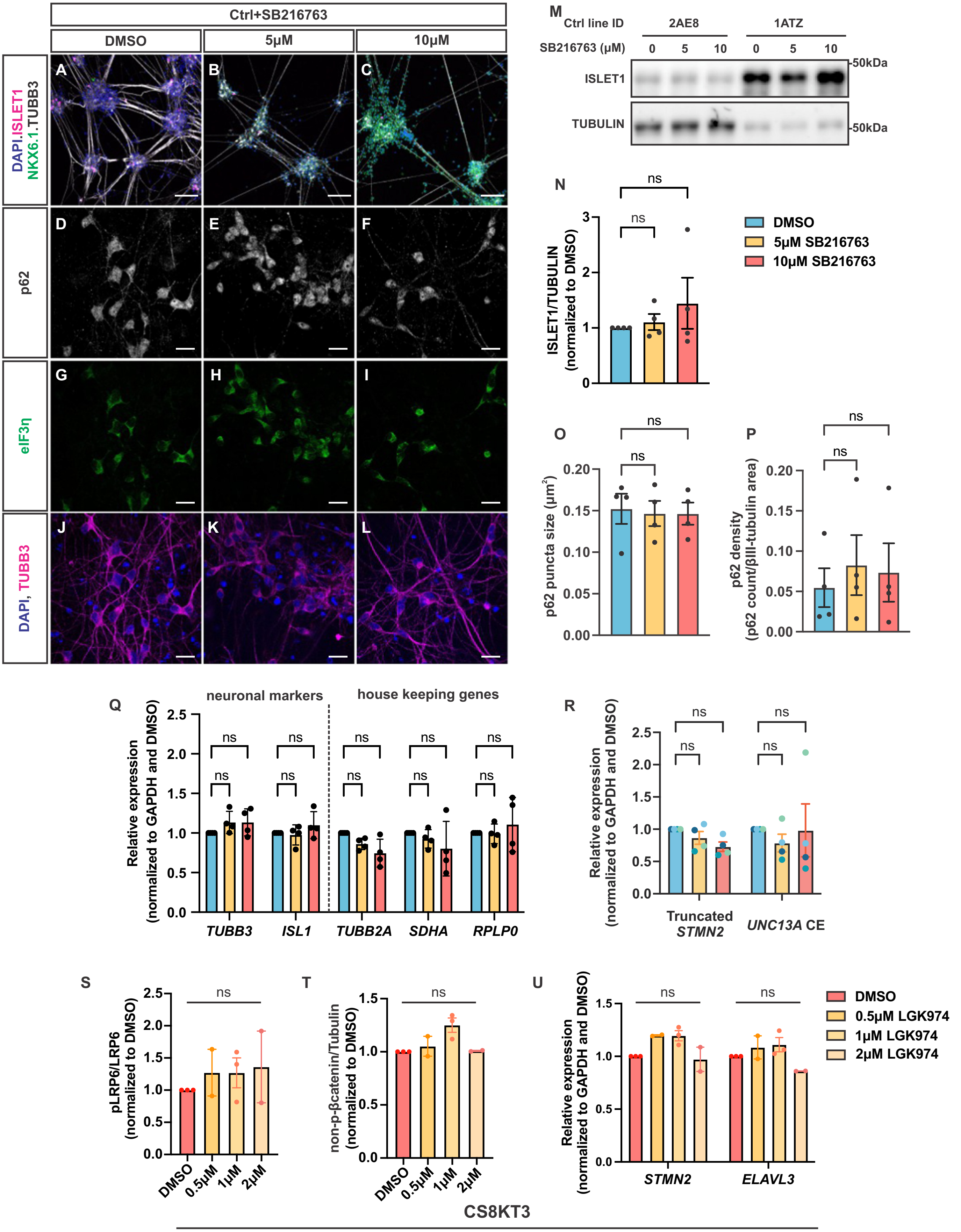
Ctrl+SB216763
DMSO
5μM
10μM
DAPI.ISLET1 NKX6.1.TUBB3
p62
eIF3η
DAPI, TUBB3
A B C D E F G H I J K L
M
Ctrl line ID 2AE8 1ATZ
SB216763 (μM) 0 5 10 0 5 10
ISLET1
TUBULIN
50kDa
N
ISLET1/TUBULIN (normalized to DMSO)
DMSO
5μM SB216763
10μM SB216763
O
p62 puncta size (μm²)
P
p62 density (p62 count/βIII-tubulin area)
Q
neuronal markers
house keeping genes
Relative expression (normalized to GAPDH and DMSO)
TUBB3 ISL1 TUBB2A SDHA RPLP0
R
Truncated STMN2
UNC13A CE
S
pLRP6/LRP6 (normalized to DMSO)
DMSO 0.5μM 1μM 2μM
T
non-p-βcatenin/Tubulin (normalized to DMSO)
U
STMN2 ELAVL3
DMSO
0.5μM LGK974
1μM LGK974
2μM LGK974
CS8KT3
ns

Figure EV5. Wnt activation and inhibition of iSNs.

(A–L) Representative images of immunocytochemical staining of Day 32 Ctrl iSNs treated with DMSO or SB216763 for ISLET1, Nkx6.1, tubulin (TUBB3), neurofilament (SMI32), stress granules (eIF3η) and autophagosome (p62). (**M**) Representative western blot of iSN protein extracts from Day 32 Ctrl lines treated with different concentrations of the GSK3-β inhibitor, SB216763, for 1 week. (**N**) Graph quantifying ISLET1 protein normalized to tubulin when treated with DMSO and 5 μM (ns $P > 0.9999$) or 10 μM of SB216763 (ns $P > 0.9999$), $n = 4$ cell lines. (**O**, **P**) Graphs quantifying autophagosome (p62) size (**O**) and density (**P**) in Ctrl iSNs treated with DMSO or SB216763. p62 size (**O**) (DMSO/5 μM) ns $P = 0.8940$, (DMSO/10 μM) ns $P = 0.8782$; density (**P**) (DMSO/5 μM) ns $P = 0.2495$, (DMSO/10 μM) ns $P = 0.4258$, $n = 4$ cell lines. (**Q**) Graphs quantifying mRNA level of motor neuron markers and other housekeeping genes in Ctrl iSNs treated with DMSO, 5 μM or 10 μM SB216763. *TUBB3* (DMSO/5 μM) ns $P = 0.4068$, (DMSO/10 μM) ns $P = 0.4001$; *ISL1* (DMSO/5 μM) ns $P = 0.9687$, (DMSO/10 μM) ns $P = 0.5995$; *TUBB2A* (DMSO/5 μM) ns $P = 0.3850$, (DMSO/10 μM) ns $P = 0.0584$; *SDHA* (DMSO/5 μM) ns $P = 0.7469$, (DMSO/10 μM) ns $P = 0.1609$; *RPLP0* (DMSO/5 μM) ns $P = 0.9944$, (DMSO/10 μM) ns $P = 0.5546$, $n = 4$ cell lines, 2 differentiations/line. (**R**) Graph quantifying cryptic exon inclusion of *STMN2* (Truncated *STMN2*) and *UNC13A* (*UNC13A* CE) in Ctrl iSNs treated with DMSO, 5 μM or 10 μM SB216763. Truncated *STMN2* (DMSO/5 μM) ns $P = 0.8259$, (DMSO/10 μM) ns $P = 0.4878$; *UNC13A* CE (DMSO/5 μM) ns $P = 0.6283$, (DMSO/10 μM) ns $P = 0.9963$ $n = 4$ cell lines, 2 differentiations/line. (**S**, **T**) Graphs quantifying phosphorylated LRP6 normalized to total LRP6 protein (DMSO/0.5 μM) ns $P > 0.9999$, (DMSO/1 μM) ns $P > 0.9999$, (DMSO/2 μM) ns $P > 0.9999$ (**S**), and non-phosphorylated β-catenin normalized to tubulin (DMSO/0.5 μM) ns $P > 0.9999$, (DMSO/1 μM) ns $P = 0.0609$, (DMSO/2 μM) ns $P > 0.9999$ (**T**) in C9ALS line CS8KT3 treated with different concentrations of the Wnt inhibitor, LGK974. $n = 2$ or 3 differentiations. (**U**) Graphs quantifying mRNA level of TDP-43 target gene expression in C9ALS line CS8KT3 treated with different concentrations of the Wnt inhibitor, LGK974. *STMN2* (DMSO/0.5 μM) ns $P = 0.0843$, (DMSO/1 μM) ns $P = 0.0535$, (DMSO/2 μM) ns $P = 0.9806$; *ELAVL3* (DMSO/0.5 μM) ns $P = 0.6698$, (DMSO/1 μM) ns $P = 0.3824$, (DMSO/2 μM) ns $P = 0.2875$. $n = 2$ or 3 differentiations. All graphs: mean ± s.e.m. (**N**) Friedmen's test with Dunn's multiple comparisons test; (**O**, **P**) RM one-way ANOVA, with Geisser-Greenhouse correction and Dunnett's multiple comparisons test; (**Q**, **R**, **U**) Two-way ANOVA with Dunnett's multiple comparisons test (**Q**, **R**) or Šidák's multiple comparisons test (**U**). (**S**, **T**) Kruskal–Wallis test with Dunn's multiple comparisons test. $n = 4$ cell lines. Scale bar (**A–C**) = 100 μm; (**D–L**) = 20 μm.

 © The Author(s)
